# Synthesis of *meta*-Aminophenol Derivatives via Cu-Catalyzed [1,3]-Rearrangement—Oxa-Michael Addition Cascade Reactions

**DOI:** 10.3390/molecules28104251

**Published:** 2023-05-22

**Authors:** Itaru Nakamura, Mai Tachibana, Riku Konta, Hiroki Tashiro, Masahiro Terada

**Affiliations:** 1 Research and Analytical Center for Giant Molecules, Graduate School of Science, Tohoku University, Sendai 980-8578, Japan; 2 Department of Chemistry, Graduate School of Science, Tohoku University, Sendai 980-8578, Japan

**Keywords:** anilines, cascade reaction, rearrangement, copper catalysts

## Abstract

Cu-catalyzed reactions of *N*-alkoxy-2-methylanilines and alcohols in the presence of catalytic amounts of IPrCuBr and AgSbF_6_ afforded the corresponding *meta*-aminophenol derivatives in good to high yields. These reactions proceed via a [1,3]-rearrangement, in which the alkoxy group migrates from the nitrogen atom to the methyl-substituted ortho position, followed by an oxa-Michael reaction of the resulting *ortho*-quinol imine intermediate.

## 1. Introduction

**Figure 1:**
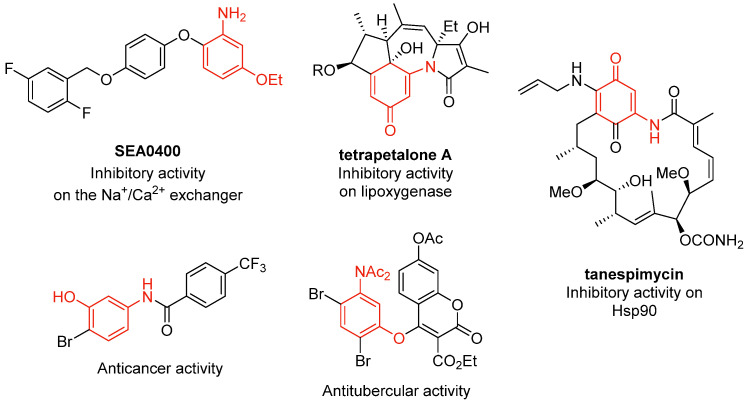
*meta*-aminophenol derivatives.

**Figure 2:**
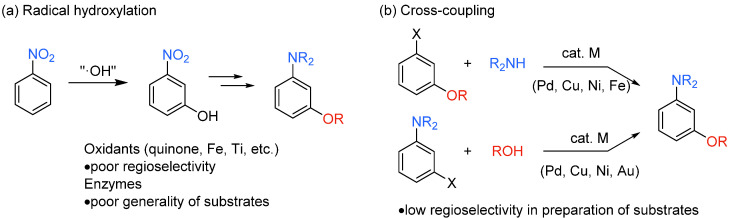
Conventional methods for the synthesis of *meta*-aminophenol derivatives.

*meta*-Aminophenol derivatives are frequently used in pharmaceutical science (Figure 1) [1,2,3,4,5]. For example, SEA0400 exhibits inhibitory activity on the Na^+^/Ca^2+^ exchanger [1], while tetrapetalone A [2] and tenaspimycin [3] inhibit lipoxygenase and Hsp90, respectively. However, performing the synthesis of *meta*-aminophenol scaffolds in an efficient and selective manner has been challenging because electrophilic substitution reactions, the most common approach to the functionalization of anilines and phenols, exhibit ortho/para preference. The radical hydroxylation of nitrobenzene, which occurs at the meta position, has significant drawbacks in terms of regioselectivity, reaction efficiency, and functional group compatibility (Figure 2a) [6,7,8]. The enzymatic oxidation of nitrobenzene can synthesize *meta*-nitrophenol with excellent site-selectivity, although this method suffers from the poor generality of substrates [9]. In this regard, transition metal-catalyzed cross-coupling reactions using *meta*-haloanilines and *meta*-halophenols are among the most frequently employed methods (Figure 2b) [10,11,12,13,14,15]. For example, Buchwald–Hartwig amination reactions of *meta*-halophenol derivatives using palladium catalysts [10,11,12,13] and Ullman-type reactions using copper catalysts [14,15] have been frequently used for the synthesis of *meta*-aminophenol derivatives. Catalytic C–O bond-forming reactions using *meta*-haloanilines have also been employed as an alternative approach to cross-coupling reactions by employing various transition metal catalysts, such as palladium [16,17], copper [18,19], nickel [20], and gold [21]. However, these reactions are also inherently associated with the selectivity issue in *meta*-haloaniline/phenol preparations. Although other approaches to functionalized *meta*-aminophenol derivatives have been recently reported [22], the development of efficient and robust methods for the synthesis of multiply substituted *meta*-aminophenol derivatives is a pressing issue.

**Figure 3:**
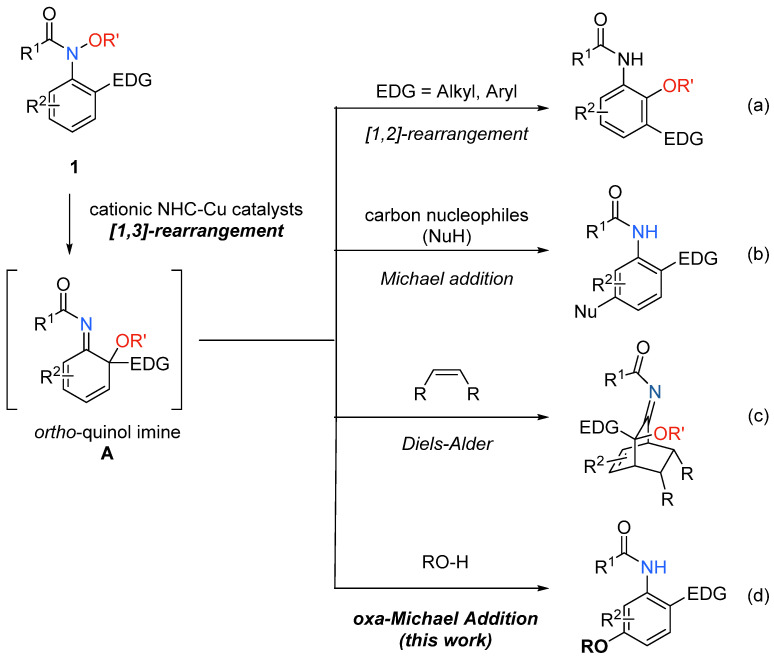
Cu-catalyzed [1,3]-alkoxy rearrangement: (**a**) domino [1,3]/[1,2] rearrangement, (**b**) [1,3]-rearrangement—Michael addition, (**c**) [1,3]-rearrangement—Diels-Alder reaction, and (**d**) [1,3]-rearrangement—oxa-Michael addition (this work).

**Figure 4:**
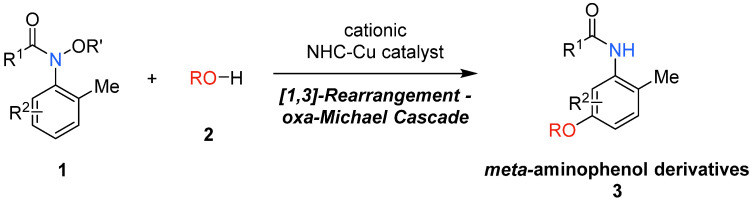
Cu-catalyzed [1,3]-rearrangement—oxa-Michael addition cascade reaction.

Against this backdrop, we designed cascade reactions, including the Cu-catalyzed [1,3]-rearrangement, as a potential approach to overcome issues related to selectivity (Figure 3) [23,24,25,26,27,28,29,30,31,32]. We have recently reported that *N*-heterocyclic carbene (NHC)-ligated cationic copper catalysts efficiently promote the [1,3]-rearrangement of *N*-alkoxyanilines [23,24,25,26,27]. Specifically, *N*-alkoxyanilines **1** having an electron-donating group (EDG) at the ortho position selectively generated functionalized *ortho*-quinol imine intermediates **A** via a [1,3]-rearrangement of the alkoxy group to the EDG-substituted ortho position [25,26,27,28]. *ortho*-quinol imine intermediates **A** underwent favorable transformations, including a [1,2]-rearrangement (Figure 3a) [25]; a Michael addition with carbon nucleophiles such as *N*-methylindole, 1,3,5-trimethoxybenzene, and dimethyl malonate (Figure 3b) [26]; and a Diels–Alder reaction with electron-rich and electron-deficient olefins [27]. It should be emphasized that the Cu-catalyzed [1,3]-rearrangement can generate functionalized quinol imine intermediates **A**, which had been inaccessible via conventional methods, such as thermally induced rearrangement [33] and oxidation [34]. Accordingly, we envisioned that the reactions with alcohols as oxygen nucleophiles would give functionalized *meta*-aminophenol derivatives in a selective manner through an oxa-Michael addition of generated *ortho*-quinol imine intermediates **A** (Figure 3d) [35,36]. Here, we report the Cu-catalyzed [1,3]-rearrangement—oxa-Michael addition—aromatization cascade reactions of *N*-alkoxyanilines **1** and alcohol nucleophiles **2**, which afforded *meta*-aminophenol derivatives **3** in good-to-acceptable yields (Figure 4).

## 2. Results and Discussion

**Scheme 1:**
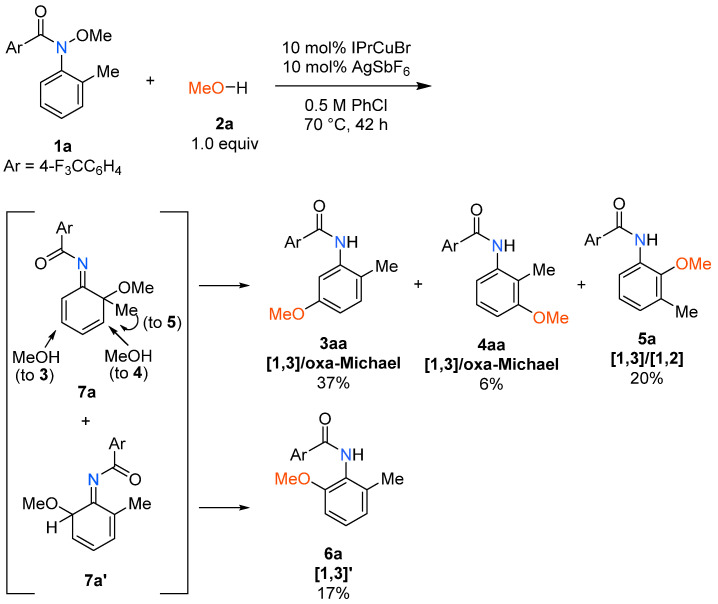
Cu-catalyzed reaction of **1a** and methanol **2a**.

Initially, the reaction of *N*-methoxy-2-methylaniline **1a** having a *p*-trifluoromethylbenzoyl group on the nitrogen atom and one equivalent of methanol **2a** in the presence of catalytic amounts of IPrCuBr [IPr: *N*,*N*′-bis(2,6-diisopropylphenyl)-imidazol-2-ylidene, 10 mol%] and AgSbF_6_ (10 mol%) in chlorobenzene at 70 °C afforded a mixture of the following four regioisomers: desired 6-methyl-3-anisidine **3aa**; 2-methyl-3-anisidine **4aa**, which was derived from the oxa-Michael addition to the meta position next to the *ortho*-methyl group; 3-methyl-2-anisidine **5a**, which was derived from domino [1,3]/[1,2]-rearrangement reactions [25]; and 6-methyl-2-anisidine **6a**, which was derived from the [1,3]-rearrangement reactions of the methoxy group to the unsubstituted ortho position (namely, [1,3]′-rearrangement) to generate *ortho*-quinol imine intermediate **7a′** (Scheme 1). Attempts to optimize the reaction conditions to selectively synthesize **3aa** were unsuccessful.

To reduce the number of regioisomers prior to the optimization of reaction conditions, we preliminary screened several starting materials **1**. To our delight, the reaction of substrate **1b** having a fluorine atom at the para position of the aniline ring at 70 °C generated two regioisomers, **3ba** and **6b**. Among the NHC ligands examined, IPr exhibited the best reactivity and product selectivity (Table 1, entry 1), whereas the use of IMes [1,3-Bis(2,4,6-trimethylphenyl)-1,3-dihydro-2*H*-imidazol-2-ylidene] and SIPr [1,3-bis(2,6-diisopropylphenyl)imidazolidine] resulted in low catalytic activity (entries 2 and 3). The reactivity was significantly affected by counteranions; less coordinative counteranions such as hexafluoroantimonate were effective for the present transformations. On the other hand, when AgBF_4_ and AgNTf_2_ were used instead of AgSbF_6_, **3ba** was formed in low chemical yields along with a considerable amount of recovered **1b** (entries 4 and 5). The reaction in 1,2-dichloroethane (DCE) proceeded quickly to afford product **3ba** in the best yield among the solvents examined (entries 6–9). Neither the chemical yield nor the product selectivity was improved when the temperature of the reaction of **1b** and **2a** was changed from 70 °C to 60 °C and 80 °C, respectively (entries 10 and 11). The reaction in the absence of either Cu or Ag did not yield desired product **3ba**; *N*-methoxyaniline **1b** was quantitatively recovered (entries 12 and 13).

The protecting group on the nitrogen atom significantly affected the product selectivity (Table 2, entries 1–4); desired *meta*-anisidines **3** were obtained with better product selectivity with carbamate-type protecting groups (entries 2–4) than with the amide-type protecting groups (Table 1 entry 6, and Table 2, entry 1). The use of one equivalent of methanol **2a** was effective, whereas the use of a large excess (five equivalents) of methanol **2a** diminished the catalytic activity (entry 2 versus entry 6). It should be noted that the reaction using half an equivalent of methanol at 80 °C afforded **3da** in the 71% yield, suggesting that the methoxy group eliminated from substrate **1d** participated in the reaction as a nucleophile (entry 8). Finally, the chemical yield was improved by increasing the scale from 0.2 mmol to 0.5 mmol (entry 9, Appendix A).

**Scheme 2:**
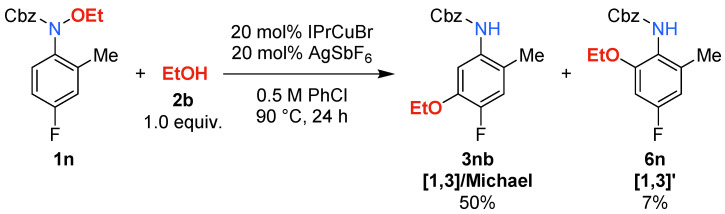
Cu-catalyzed reaction of *N*-ethoxyaniline **1n** and ethanol **2b**.

The optimized conditions (Table 2, entry 9) were applied to the reactions of various *N*-methoxyanilines **1**, as summarized in Table 3. The reactions of substrates **1g** and **1h**, having a methyl and a phenyl group, respectively, at the para position, proceeded at 70 °C, affording corresponding *meta*-anisidines **3ga** and **3ha** in good yields (entries 1 and 2). Substrate **1j**, having a bromo group, was converted into desired product **3ja** when the loading amount of the Cu catalyst was increased (20 mol%, entry 7). The chemical yield of **3ia**, which has a chloro group at the para position, was slightly improved when the reaction was performed at 90 °C using chlorobenzene instead of DCE as the solvent (entries 3–5). An iodo group (**1k**) was tolerated under the present reaction conditions, affording multi-substituted *meta*-aminophenol derivative **3ka** in an acceptable yield (entry 8). 3-Anisidine **3la**, which has an alkynyl group at the para position of the nitrogen atom, could also be generated (entry 9). In contrast, substrate **1m** having a methoxycarbonyl group at the para position, exclusively afforded domino-rearrangement byproduct **5m** (entry 10). The reaction of *N*-ethoxyaniline **1n** and ethanol **2b** afforded corresponding 3-ethoxyaniline **3nb** in a good yield (Scheme 2).

The reaction of *N*-methoxyaniline **1d** and one equivalent of 2-phenethyl alcohol **2c** afforded 3-phenylethoxyaniline **3dc** in a 51% yield, along with **3da**, which was produced through the oxa-Michael addition of methanol derived from **1d**, in a 14% yield (Table 4, entry 1). The use of two equivalents of **2c** did not improve the product selectivity (entry 2). The reaction of **1d** and allyl alcohol **2d** also afforded 3-allyloxyaniline **3dd** as the major product (entry 3). These results suggest that substrates **1** react more preferentially with external alcohols **2c** and **2d** than with methanol **2a** derived from **1**, although the product selectivity depends on the structure of **2**. When *tert*-butanol (**2e**) and phenol (**2f**) were employed as an alcohol nucleophile, the reactions gave a mixture of unidentified products (entries 4 and 5).

**Figure 5:**
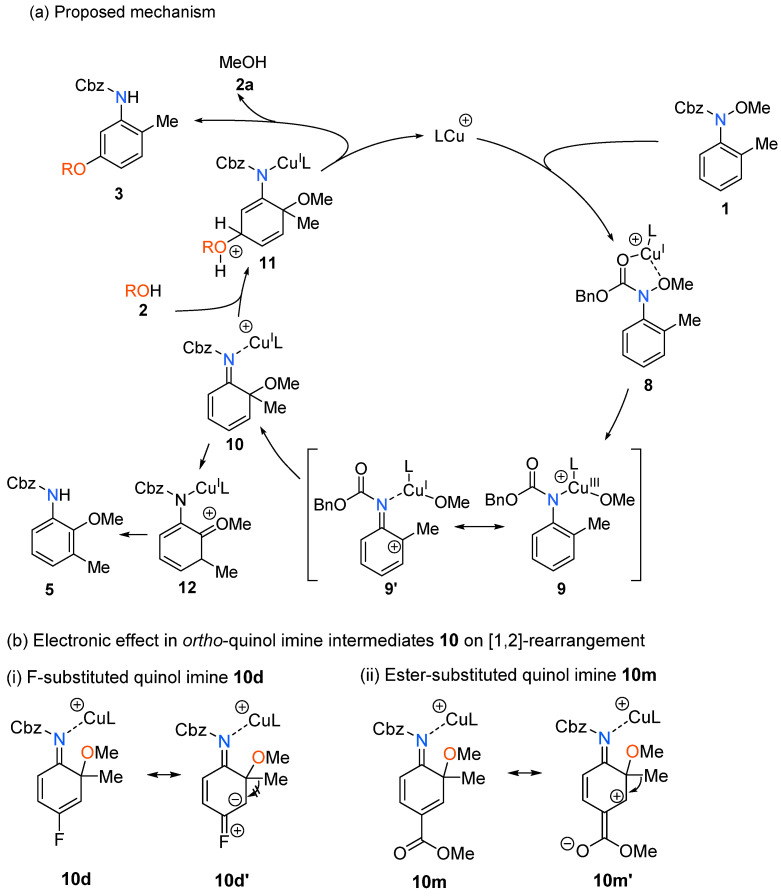

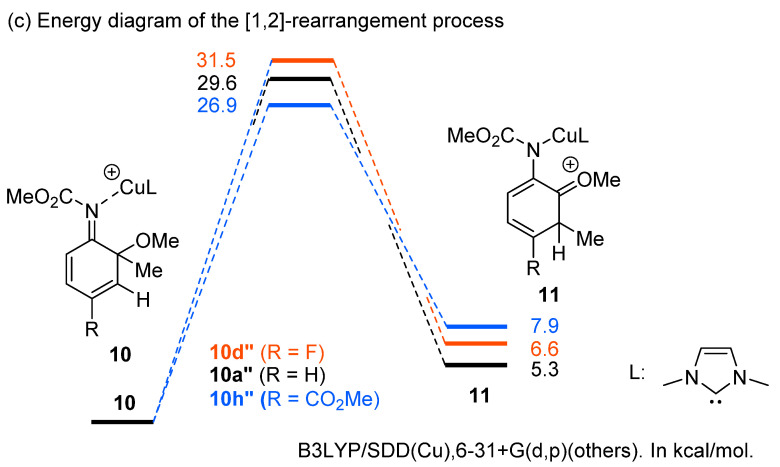
Mechanistic studies.

A proposed mechanism for the Cu-catalyzed reactions of *N*-methoxyanilines **1** and alcohol nucleophiles **2** is illustrated in Figure 5a. The cationic copper catalyst coordinates to **1** to form chelate complex **8**, and this is followed by an oxidative addition of the N–O bond to the Cu(I) catalyst to form Cu(III) complex **9** [29]. Because of the contribution of canonical form **9′**, a C–O bond is formed at the methyl-substituted ortho position, generating *ortho*-quinol imine intermediates **10** that coordinate to the cationic Cu catalyst. Electrophilically activated *ortho*-quinol imine intermediates **10** undergo nucleophilic addition of **2** to form Cu enamide species **11**. Finally, proton transfer and elimination of methanol **2a** give products **3** along with the regenerated cationic Cu catalyst. Byproducts **5** are formed via a Cu-catalyzed [1,2]-rearrangement of *ortho*-quinol imine intermediates **12** [25]. The product selectivity of the present reaction system was greatly influenced by the para substituent; a fluorine atom (**1d**) was effective in suppressing the formation of the domino [1,3]/[1,2]-rearrangement product (**5**, Table 2, entry 9). This is possibly because the mesomeric electron-donating effect of the fluorine atom increases the electron density of the carbon next to the quaternary carbon in *ortho*-quinol imine intermediates **10d**/**10d′**, decelerating the [1,2]-rearrangement of the methyl group (Figure 5(bi)). In contrast, when an ester group was present, the selective formation of the domino rearrangement product (**5m**) occurred even in the presence of methanol (Table 3, entry 10), presumably because of the undesired [1,2]-rearrangement reaction facilitated by the electron-withdrawing effect (Figure 5(bii)). In addition, because substrate **1m** reacted with stronger carbon nucleophiles, such as 1,3,5-trimethoxybenzene, via [1,3]-rearrangement/Michael addition [26], the low nucleophilicity of alcohols **2** also causes the selective formation of byproduct **5m**. Our preliminary DFT calculations for the [1,2]-rearrangement of *para*-substituted *ortho*-quinol imine intermediates **10** were in good agreement with these experimental results (Figure 5c). The reaction of *N*-methoxyaniline **1d** and alcohol **2c** afforded **3dc** (51%) in a higher yield than **3da** (14%) derived from the reaction of **1d** and generated methanol **2a** (Table 4, entry 1). Because the product selectivity is dependent on the structure of external alcohols **2c** and **2d**, the elimination of methanol **2a** from enamide intermediates **11** may be mediated by external alcohol **2**, affecting the concentration of methanol **2a**. Further investigations to understand the reactivity of key intermediates **10** are under way in our laboratory.

## 3. Materials and Methods

The general procedure for the Cu-catalyzed reactions of *N*-methoxyaniline **1d** and methanol **2a** is as follows: DCE (1.0 mL) and methanol **2a** (20.3 μL, 0.5 mmol) were added to a mixture of IPrCuBr (26.6 mg, 0.05 mmol), AgSbF_6_ (24.0 mg, 0.05 mmol), and **1d** (144.8 mg, 0.5 mmol) under an argon atmosphere, and the mixture was stirred at 70 °C for 48 h. After complete consumption of **1d**, as monitored via TLC, the mixture was passed through a pad of silica gel with ethyl acetate (50 mL). The solvents were removed in vacuo, and the crude product was purified via silica gel column chromatography using hexane/ethyl acetate (20/1) as an eluent to afford **3da** (123.3 mg, 0.426 mmol, 85% yield) in an analytically pure form (Supplementary Materials).

## 4. Conclusions

In conclusion, we have developed a new approach to prepare *meta*-aminophenol derivatives via Cu-catalyzed cascade reactions involving [1,3]-rearrangement, oxa-Michael addition, and aromatization in an efficient manner. Because a variety of functional groups are tolerated in this transformation, the present method is potentially useful for synthesizing a new class of *meta*-aminophenol derivatives.

## Figures and Tables

**Table 1:** Optimization of reaction conditions.

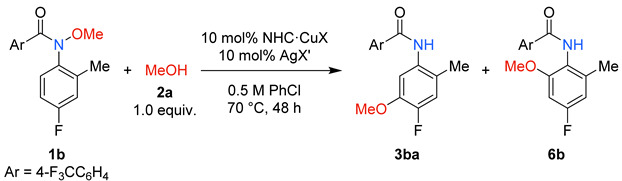							
**Entry**	**NHC·CuX**	**AgX′**	**Solvent**	**Temp. (°C)**	**3ba (%) ^a^**	**6b (%) ^a^**	**1b (%) ^a^**
1	IPrCuBr	AgSbF_6_	PhCl	70	70	28	<1
2	IMesCuCl	AgSbF_6_	PhCl	70	21	3	76
3	SIPrCuCl	AgSbF_6_	PhCl	70	13	5	47
4	IPrCuBr	AgBF_4_	PhCl	70	43	13	37
5	IPrCuBr	AgNTf_2_	PhCl	70	<10	<15	- ^b^
6	IPrCuBr	AgSbF_6_	DCE	70 ^c^	76	22	<2
7	IPrCuBr	AgSbF_6_	toluene	70	63	18	11
8	IPrCuBr	AgSbF_6_	1,4-dioxane	70	59	19	7
9	IPrCuBr	AgSbF_6_	EtOAc	70	29	12	38
10	IPrCuBr	AgSbF_6_	DCE	60 ^d^	<78	18	3
11	IPrCuBr	AgSbF_6_	DCE	80 ^e^	75	22	6
12	IPrCuBr	-	DCE	70	<1	<1	99
13	-	AgSbF_6_	DCE	70	<1	<1	92

^a 1^H NMR yields using CH_2_Br_2_ as the internal standard. ^b^ The yield was not determined, but a considerable amount of **1b** was recovered; ^c^ 17 h; ^d^ 40 h; ^e^ 10 h.

**Table 2:** Optimization of the protecting group of **1** and the molar amount of methanol **2a**.

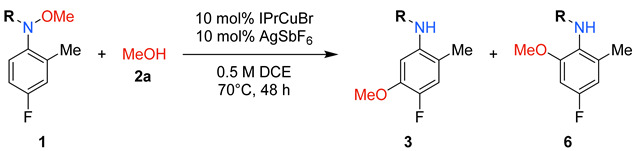						
**Entry**	**1**	**R**	**2a (Equiv)**	**3 (%) ^a^**	**6 (%) ^a^**	**1 (%) ^a^**
1 ^b^	**1c**	Bz	1	**3ca** (73)	**6c** (12)	<1
2	**1d**	Cbz	1	**3da** (72)	<1	<1
3	**1e**	Troc	1	**3ea** (54)	<1	27
4	**1f**	Alloc	1	**3fa** (26)	**6f** (6)	32
5	**1d**	Cbz	2	**3da** (48)	**6d** (4)	46
6	**1d**	Cbz	5	**3da** (18)	**6d** (3)	29
7	**1d**	Cbz	0.5	**3da** (50)	**6d** (3)	<31
8 ^d^	**1d**	Cbz	0.5	**3da** (71)	**6d** (9)	<11
9 ^e^	**1d**	Cbz	1	**3da** (85) ^c^	<1	<1

^a 1^H NMR yields using CH_2_Br_2_ as the internal standard. The reactions were conducted on a 0.2 mmol scale; ^b^ 17 h. ^c^ Isolated yield. ^d^ At 80 °C. ^e^ The reaction was conducted at a 0.5 mmol scale.

**Table 3:** Cu-catalyzed reactions of *N*-methoxyanilines **1g-m** and methanol **2a** ^a^.

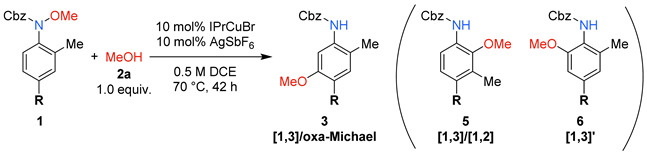						
**Entry**	**1**	**R**	**Temp (°C)**	**3 (%) ^b^**	**1 (%) ^c^**	**Byproduct (%) ^c^**
1 ^d,e^	**1g**	Me	70	**3ga** (64)	<1	**5g** (<3)
2 ^e^	**1h**	Ph	70	**3ha** (45)	<1	**5h** (28), **6h** (27)
3	**1i**	Cl	70	**3ia** (23)	36	
4 ^f^	**1i**	Cl	70	**3ia** (30)	19	
5 ^e,f,g^	**1i**	Cl	90 ^c^	**3ia** (61)	<1	
6	**1j**	Br	70	**3ja** (40)	32	
7 ^f^	**1j**	Br	70	**3ja** (60)	<1	
8 ^f^	**1k**	I	70	**3ka** (42)	6	**5k** (8)
9 ^g,h^	**1l**	-CºCPh	90	**3la** (28)	<1	
10 ^g,i^	**1m**	CO_2_Me	90	<1	9	**5m** (80)

^a^ The reaction of **1** (0.5 mmol) and **2a** (0.5 mmol) was carried out in the presence of IPrCuBr (10 mol%) and AgSbF_6_ (10 mol%) in DCE at 70 °C for 18 h. ^b^ Isolated yields. ^c 1^H NMR yields using CH_2_Br_2_ as the internal standard. ^d^ The reaction was conducted at a 2.0 mmol scale; ^e^ 18 h; ^f^ 20 mol% IPrCuBr and 20 mol% AgSbF_6_ were used. ^g^ Chlorobenzene was used, instead of DCE; ^h^ 48 h; ^i^ 24 h.

**Table 4:** Cu-catalyzed reactions of *N*-methoxyanilines **1d** and alcohols **2**.

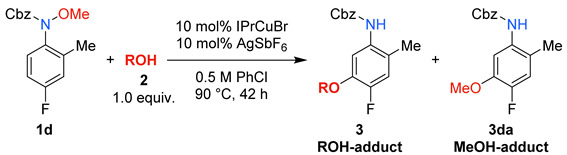				
**Entry**	**2**	**ROH**	**3 (%) ^a^**	**3da (%) ^a^**
1	**2c**	PhCH_2_CH_2_OH	**3dc** (51)	14
2	**2c** ^b^	PhCH_2_CH_2_OH	**3dc** (51)	7
3	**2d**	H_2_C=CHCH_2_OH	**3dd** (35)	23
4	**2e**	*t*BuOH	- ^c^	- ^c^
5	**2f**	PhOH	- ^c^	- ^c^

^a^ The reaction of **1d** (0.5 mmol) and **2** (0.5 mmol) was carried out in the presence of IPrCuBr (10 mol%) and AgSbF_6_ (10 mol%) in PhCl at 90 °C for 42 h. ^b^ Two equivalents of **2c** were used. ^c^ A mixture of unidentified products was obtained.

## Data Availability

The data presented in this study are available in this article.

## Supplementary Materials

The following supporting information can be downloaded at: https://www.mdpi.com/article/10.3390/molecules28104251/s1, SI (pdf). All experimental data, detailed experimental procedures, and ^1^H NMR, ^13^C NMR, ^19^F NMR spectral are available online.

## Sample Availability

Samples of the compounds **3aa-ma**, **3dc**, and **3dd** are available from the authors.

## Appendix A

It is assumed that the larger reaction scale reduces influence of trace water on the reaction system, which can cause deactivation of the Cu catalysts and byproduct formation.
