# Supplementary material for: Synthesis of meta-Aminophenol Derivatives via Cu-Catalyzed [1,3]-Rearrangement—Oxa-Michael Addition Cascade Reactions
Source: Molecules. 2023 May 22;28(10):4251. doi: 10.3390/molecules28104251 (PMC10221852; doi:10.3390/molecules28104251)
Supplement: Supplementary file 1 [file molecules-28-04251-s001.zip › molecules-2397302-supplementary.pdf]

Synthesis of *meta*-Aminophenol Derivatives by Cu-catalyzed [1,3]-Rearrangement - oxa-Michael Addition Cascade Reactions

Itaru Nakamura,[1,*] Mai Tachibana,[2] Riku Konta,[2] Hiroki Tashiro,[2] and Masahiro Terada[2]

[1]Research and Analytical Center for Giant molecules, Graduate School of Science Tohoku University, Sendai 980-8578, Japan

[2]Department of Chemistry, Graduate School of Science, Tohoku University, Sendai 980-8578, Japan

Supporting Information

1. General information	S2
2. General procedure	S3
3. Computational studies	S4
4. Analytical data	S16
5. NMR charts	S28

1.General information

^{1}H and ^{13}C NMR spectra were recorded on JEOL JNM-ECS400 (600 MHz for ^{1}H and 100 MHz for ^{13}C) spectrometer. Chemical shifts are reported in ppm relative to Me_4Si (for ^{1}H, δ 0.00), and $CDCl_3$ (for ^{13}C, δ 77.00). ^{1}H NMR data are reported as follows: chemical shift, integration, multiplicity (s = singlet, d = doublet, t = triplet, q = quartet, sext = sextet, br = broad, m = multiplet) and coupling constants (Hz). Infrared (IR) spectra were recorded on a JASCO FT/IR- 4100 spectrometer. High-resolution mass spectra analysis was performed on a Bruker Daltonics APEX III FT-ICR-MS spectrometer and Bruker Daltonics solariX FT-ICR-MS spectrometer at the Instrumental Analysis Center for Chemistry, Graduate School of Science, Tohoku University. Flash column chromatography was performed on silica gel 60N (Merck 40-63 μm or Kanto 40-50 μm). Analytical thin layer chromatography (TLC) was performed on Merck precoated TLC plates (silica gel 60 F254). All reactions were carried out under argon atmosphere.

Materials

Anhydrous PhCl and 1,2-dichloroethane (DCE) were purchased from Aldrich and used as received. IPrCuBr was prepared in accordance with the literature method.[1] Substrates **1** were synthesized in accordance with the literature method.[1] $AgSbF_6$ was purchased from Aldrich.

[1] Nakamura, I.; Jo, T.; Ishida, Y.; Tashiro, H.; Terada, M. *Org. Lett.* **2017**, *19*, 3059.

2.General procedure

General procedure for Cu-catalyzed reaction of **1** and alcohol **2**

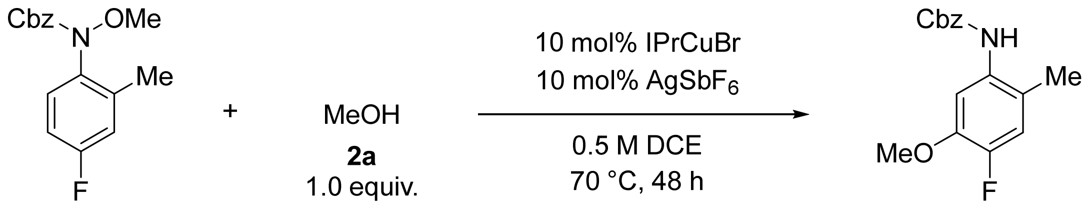

1d **2a** 1.0 equiv. **3da** **[1,3]/Michael**

To a mixture of **1d** (144.8 mg, 0.5 mmol), IPrCuBr (26.6 mg, 0.05 mmol), $AgSbF_6$ (24.0 mg, 0.05 mmol), and methanol (**2a**) (20.3 μL, 0.5 mmol) in a pressure vial was added DCE (1.0 mL) under argon atmosphere. After stirring at 70 °C for 48 h, the reaction mixture was passed through a short pad of silica gel with EtOAc (50 mL). Then, the solvent was evaporated in vacuo. The crude mixture was purified by silica gel flash column chromatography using hexane/EtOAc (20/1) as eluent to give **3da** (0.426 mmol 123.3 mg, 85%) in an analytically pure form.

3. Computational studies

3-1 General

Computations were carried out using B3LYP as implemented in the GAUSSIAN 09 software package.[1] Copper atom was described with the SDD basis set[2] with the associated effective core potential and other atoms were described with 6-31G+(d,p) basis in the geometry optimization and frequency calculations. Transition states were found by a Berny algorithm after scanning potential energy surface from reactants. All transition states were verified by the intrinsic reaction coordinate (IRC) method.

[1.] Gaussian 09, Revision C.01,
M. J. Frisch, G. W. Trucks, H. B. Schlegel, G. E. Scuseria, M. A. Robb, J. R. Cheeseman, G. Scalmani, V. Barone, B. Mennucci, G. A. Petersson, H. Nakatsuji, M. Caricato, X. Li, H. P. Hratchian, A. F. Izmaylov, J. Bloino, G. Zheng, J. L. Sonnenberg, M. Hada, M. Ehara, K. Toyota, R. Fukuda, J. Hasegawa, M. Ishida, T. Nakajima, Y. Honda, O. Kitao, H. Nakai, T. Vreven, J. A. Montgomery, Jr., J. E. Peralta, F. Ogliaro, M. Bearpark, J. J. Heyd, E. Brothers, K. N. Kudin, V. N. Staroverov, T. Keith, R. Kobayashi, J. Normand, K. Raghavachari, A. Rendell, J. C. Burant, S. S. Iyengar, J. Tomasi, M. Cossi, N. Rega, J. M. Millam, M. Klene, J. E. Knox, J. B. Cross, V. Bakken, C. Adamo, J. Jaramillo, R. Gomperts, R. E. Stratmann, O. Yazyev, A. J. Austin, R. Cammi, C. Pomelli, J. W. Ochterski, R. L. Martin, K. Morokuma, V. G. Zakrzewski, G. A. Voth, P. Salvador, J. J. Dannenberg, S. Dapprich, A. D. Daniels, O. Farkas, J. B. Foresman, J. V. Ortiz, J. Cioslowski, and D. J. Fox, Gaussian, Inc., Wallingford CT, 2010.
2. A. Bergner, M. Dolg, W. Kuechle, H. Stoll, H. Preuss, *Mol. Phys.*, 1993, **80**, 1431.

3-2. Calculated geometries and energies

Figure S1. Reaction coordinate for Cu-catalyzed [1,2]-rearrangement of *ortho*-quinol imine intermediate **10** of **1ba** at the level of B3LYP/SDD(Cu),6-31+(d,p)(others).

(c) energy diagram of the [1,2]-rearrangement process

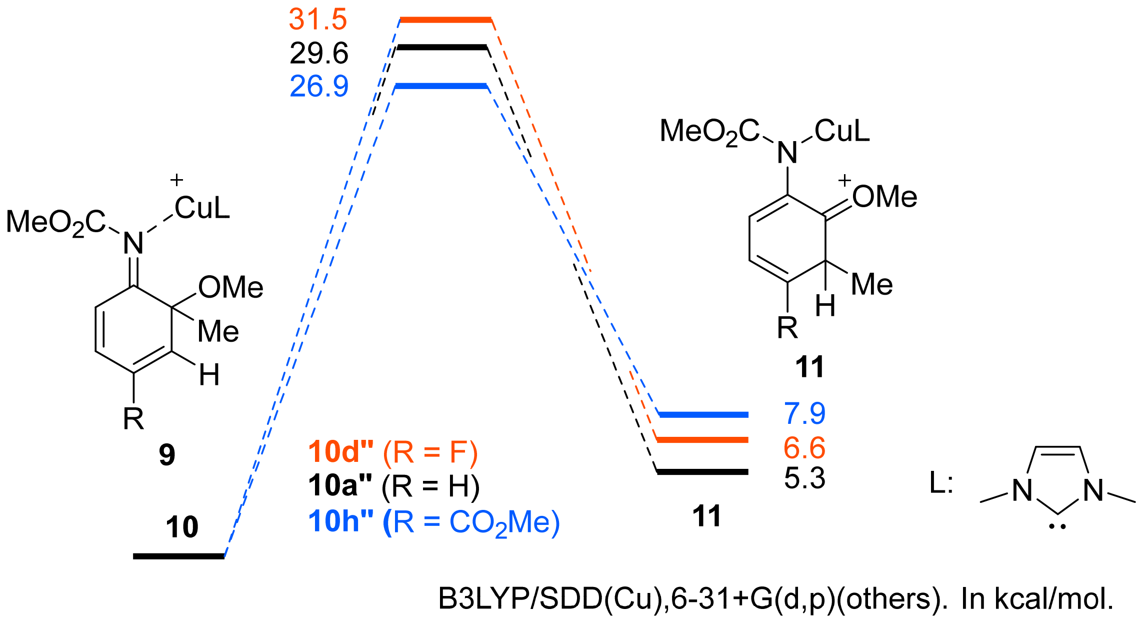

Table S1. Calculated energies of intermediates and transition states

Compound	SCF energy / a.u.	E(ZPVE) / a.u.	H / a.u.	G(298) / a.u.	Imaginary frequency /cm^{-1}
**10d”**	-1270.609516	-1270.268073	-1270.241336	-1270.328190	-
**TS$_{\text{10d”-11d”}}$**	-1270.558807	-1270.218957	-1270.192600	-1270.278000	-580.87
**11d”**	-1270.599974	-1270.257867	-1270.231149	-1270.317702	-
**10a”**	-1171.375649	-1171.025887	-1171.000023	-1171.084731	-
**TS$_{\text{10a”-11a”}}$**	-1171.328839	-1170.980288	-1170.954958	-1171.037584	-546.76
**11a”**	-1171.363425	-1171.013279	-1170.987460	-1171.072340	-
**10h”**	-1399.259952	-1398.867469	-1398.836961	-1398.932699	-
**TS$_{\text{10h”-11h”}}$**	-1399.216875	-1398.825783	-1398.795699	-1398.889809	-527.82
**11h”**	-1399.248730	-1398.855667	-1398.825344	-1398.920072	-

Figure S2. Geometry of the computed intermediates and transition states

10d”

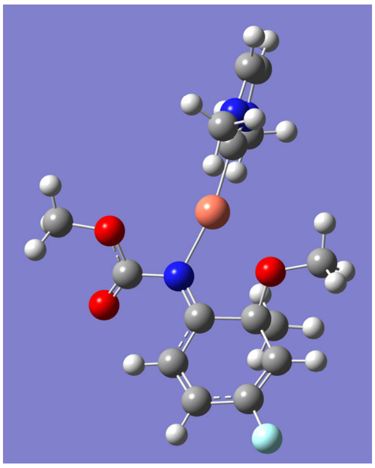

TS$_{10d”-11d”}$

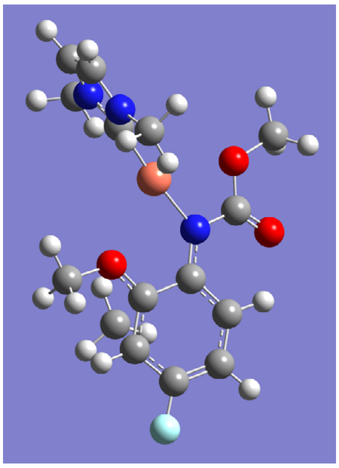

11d”

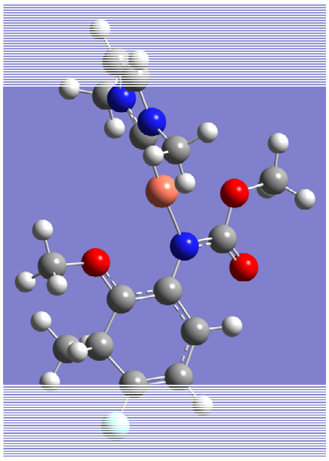

10a”

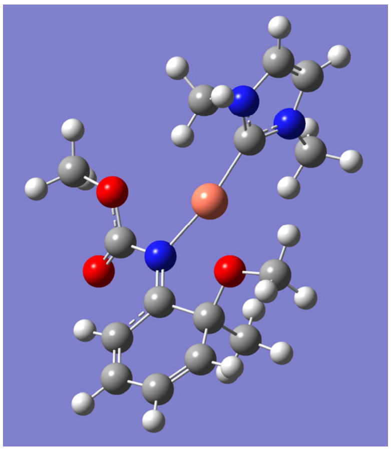

TS$_{10a”-11a”}$

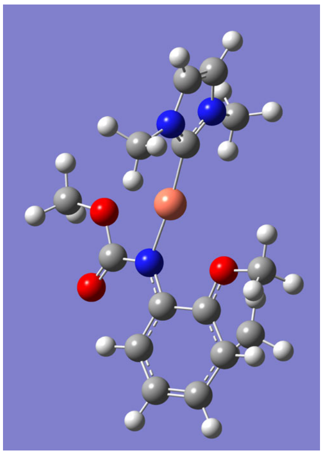

11a”

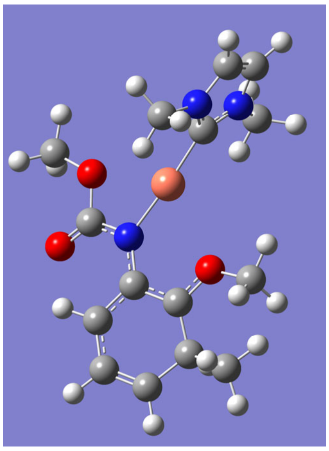

10h”

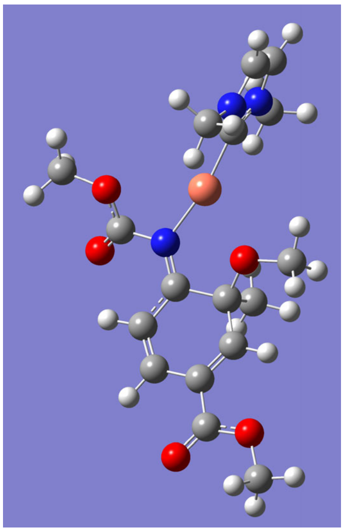

TS$_{10h”-11h”}$

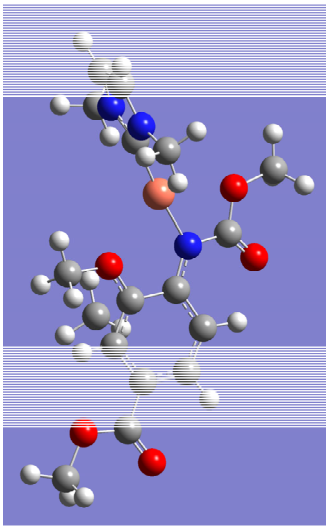

11h”

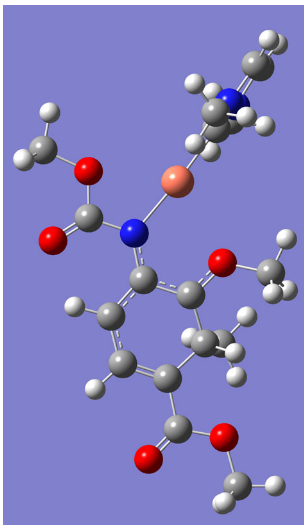

3-3. Cartesian Coordinates

10d”

Center Number	Atomic Number	Atomic Type	Coordinates (Angstroms)		
			X	Y	Z
1	6	0	-4.880993	-0.983386	0.457764
2	1	0	-5.733385	-1.152653	1.097405
3	6	0	-4.748185	-1.085941	-0.891085
4	1	0	-5.463177	-1.359925	-1.651408
5	7	0	-3.646259	-0.589791	0.949526
6	7	0	-3.435302	-0.754322	-1.186648
7	6	0	-2.739821	-0.442750	-0.056847
8	29	0	-0.916088	0.110376	0.057203
9	6	0	1.994545	-1.050692	0.418597
10	6	0	1.979807	0.463628	0.142497
11	6	0	3.241002	1.124588	-0.118162
12	1	0	3.275101	2.204748	-0.138308
13	6	0	4.348824	0.394251	-0.412638
14	1	0	5.285082	0.882673	-0.666577
15	6	0	4.315632	-1.049041	-0.426824
16	6	0	3.228396	-1.753503	-0.089732
17	1	0	3.257848	-2.836798	-0.074679
18	6	0	0.724128	2.483487	0.182418
19	8	0	1.491200	3.243590	0.731562
20	6	0	1.967101	-1.190979	1.971499
21	1	0	1.047780	-0.741940	2.358480
22	1	0	2.010320	-2.243268	2.261417
23	1	0	2.825587	-0.684465	2.420665
24	7	0	0.811670	1.056914	0.195854
25	8	0	0.786812	-1.566428	-0.163299
26	6	0	0.555981	-2.980961	-0.111698
27	1	0	1.219694	-3.516999	-0.797444
28	1	0	0.664147	-3.378833	0.902491
29	1	0	-0.475924	-3.119304	-0.436550
30	6	0	-2.892662	-0.720547	-2.544324
31	1	0	-3.419038	0.028121	-3.142118
32	1	0	-1.836158	-0.457378	-2.491119
33	1	0	-2.997498	-1.701150	-3.015703
34	6	0	-3.375115	-0.360904	2.367774
35	1	0	-3.497201	-1.289574	2.931615
36	1	0	-2.349776	-0.006221	2.472227
37	1	0	-4.057669	0.396222	2.761922
38	8	0	-0.377368	2.845744	-0.483448
39	6	0	-0.636178	4.272302	-0.545967
40	1	0	0.184025	4.778434	-1.058296
41	1	0	-1.563127	4.367069	-1.108597
42	1	0	-0.745969	4.674666	0.462771
43	9	0	5.458356	-1.659389	-0.799595

TS$_{10d''-11d}$

Center Number	Atomic Number	Atomic Type	Coordinates (Angstroms)		
			X	Y	Z
1	6	0	-4.901896	-1.172559	0.492807
2	1	0	-5.735305	-1.353623	1.153895
3	6	0	-4.775348	-1.356771	-0.848062
4	1	0	-5.477768	-1.728022	-1.578251
5	7	0	-3.691485	-0.665837	0.939355
6	7	0	-3.490964	-0.958043	-1.183699
7	6	0	-2.806055	-0.524992	-0.087403
8	29	0	-1.044429	0.193144	-0.021599
9	6	0	2.039746	-0.935839	0.116315
10	6	0	1.930869	0.501931	-0.159516
11	6	0	3.121184	1.131433	-0.570002
12	1	0	3.079432	2.183916	-0.809451
13	6	0	4.349229	0.457495	-0.696125
14	1	0	5.227641	0.995119	-1.038576
15	6	0	4.447813	-0.877647	-0.380349
16	6	0	3.322046	-1.592038	0.064092
17	1	0	3.383911	-2.670748	0.137060
18	6	0	0.585853	2.457803	-0.008887
19	8	0	1.474655	3.294488	-0.038630
20	6	0	2.913539	-0.995442	1.925932
21	1	0	1.904236	-0.915443	2.330530
22	1	0	3.429783	-1.868256	2.319840
23	1	0	3.487624	-0.078273	2.007255
24	7	0	0.697295	1.071267	-0.019805
25	8	0	0.882483	-1.609463	0.190749
26	6	0	0.863445	-3.049806	0.225302
27	1	0	1.344018	-3.466972	-0.664282
28	1	0	1.340992	-3.426789	1.135335
29	1	0	-0.192524	-3.315832	0.234357
30	6	0	-2.968147	-0.968776	-2.548968
31	1	0	-3.547779	-0.290008	-3.180109
32	1	0	-1.930690	-0.634992	-2.524701
33	1	0	-3.016075	-1.979518	-2.962780
34	6	0	-3.425063	-0.306583	2.330994
35	1	0	-3.535822	-1.182962	2.975211
36	1	0	-2.404505	0.069974	2.401125
37	1	0	-4.117459	0.473846	2.657162
38	8	0	-0.729113	2.792737	0.069635
39	6	0	-1.013237	4.208354	0.105547
40	1	0	-0.637012	4.694025	-0.797310
41	1	0	-2.099023	4.280840	0.158339
42	1	0	-0.551597	4.665936	0.983195
43	9	0	5.622707	-1.540729	-0.445168

11d”

Center Number	Atomic Number	Atomic Type	Coordinates (Angstroms)		
			X	Y	Z
1	6	0	4.949906	-1.262230	-0.474708
2	1	0	5.765212	-1.477404	-1.148031
3	6	0	4.848277	-1.409625	0.872966
4	1	0	5.558018	-1.777881	1.597551
5	7	0	3.741330	-0.741103	-0.908847
6	7	0	3.580468	-0.974076	1.224980
7	6	0	2.881521	-0.554478	0.132463
8	29	0	1.139161	0.204044	0.070877
9	6	0	-2.039693	-0.849641	-0.114646
10	6	0	-1.831817	0.516608	0.197814
11	6	0	-2.947981	1.178243	0.757153
12	1	0	-2.793956	2.191598	1.104584
13	6	0	-4.245403	0.616936	0.919902
14	1	0	-5.040747	1.196709	1.374733
15	6	0	-4.449411	-0.655705	0.516941
16	6	0	-3.405111	-1.468110	-0.152335
17	1	0	-3.392330	-2.466537	0.303319
18	6	0	-0.511913	2.448850	-0.114943
19	8	0	-1.438567	3.251681	-0.171898
20	6	0	-3.842077	-1.626659	-1.661797
21	1	0	-3.116939	-2.218691	-2.220494
22	1	0	-4.811568	-2.127112	-1.696027
23	1	0	-3.926098	-0.643397	-2.129377
24	7	0	-0.579506	1.082813	0.043114
25	8	0	-0.979829	-1.557094	-0.468674
26	6	0	-0.970410	-3.003468	-0.488806
27	1	0	-1.273074	-3.394725	0.486632
28	1	0	-1.611680	-3.395680	-1.279248
29	1	0	0.065781	-3.270496	-0.689267
30	6	0	3.084390	-0.942801	2.599606
31	1	0	3.705025	-0.280689	3.209035
32	1	0	2.062351	-0.563933	2.588624
33	1	0	3.096347	-1.948523	3.028222
34	6	0	3.453897	-0.405698	-2.302626
35	1	0	3.560703	-1.292483	-2.932985
36	1	0	2.430398	-0.035853	-2.365519
37	1	0	4.137748	0.372576	-2.651323
38	8	0	0.788099	2.832436	-0.233172
39	6	0	1.010191	4.245610	-0.422608
40	1	0	0.614337	4.811325	0.423672
41	1	0	2.092055	4.359101	-0.487322
42	1	0	0.530878	4.587138	-1.342660
43	9	0	-5.638666	-1.255439	0.628825

10a”

Center Number	Atomic Number	Atomic Type	Coordinates (Angstroms)		
			X	Y	Z
1	6	0	-4.703747	-0.651881	0.550910
2	1	0	-5.538263	-0.806972	1.217140
3	6	0	-4.644617	-0.620735	-0.806686
4	1	0	-5.418035	-0.742086	-1.549289
5	7	0	-3.413765	-0.434357	1.009314
6	7	0	-3.319935	-0.386271	-1.140678
7	6	0	-2.544489	-0.266310	-0.026378
8	29	0	-0.672949	0.100576	0.041731
9	6	0	2.128266	-1.347047	0.227046
10	6	0	2.246656	0.171600	0.030218
11	6	0	3.556134	0.714748	-0.236977
12	1	0	3.695570	1.786155	-0.206172
13	6	0	4.578739	-0.107447	-0.605596
14	1	0	5.538660	0.334639	-0.858443
15	6	0	4.433070	-1.544490	-0.712241
16	6	0	3.270305	-2.129340	-0.370243
17	1	0	3.155984	-3.207378	-0.421284
18	6	0	1.190817	2.292673	0.235558
19	8	0	2.052308	2.946145	0.783032
20	6	0	2.172115	-1.565416	1.772596
21	1	0	1.324515	-1.046930	2.229946
22	1	0	2.121037	-2.630255	2.011309
23	1	0	3.099829	-1.169695	2.193930
24	7	0	1.140304	0.869241	0.159830
25	8	0	0.848108	-1.724268	-0.303889
26	6	0	0.493657	-3.112803	-0.314746
27	1	0	1.039360	-3.656754	-1.092492
28	1	0	0.657525	-3.588187	0.657990
29	1	0	-0.572365	-3.140672	-0.544342
30	6	0	-2.839311	-0.260388	-2.515994
31	1	0	-3.309030	0.598480	-3.002539
32	1	0	-1.759359	-0.113511	-2.495374
33	1	0	-3.069708	-1.168321	-3.079336
34	6	0	-3.054843	-0.383767	2.425454
35	1	0	-3.270036	-1.342447	2.904880
36	1	0	-1.988823	-0.171001	2.505355
37	1	0	-3.616780	0.407954	2.927551
38	8	0	0.095609	2.800044	-0.344894
39	6	0	-0.026064	4.244298	-0.304178
40	1	0	0.812168	4.705428	-0.829855
41	1	0	-0.966878	4.463785	-0.805954
42	1	0	-0.044854	4.587680	0.731887
43	1	0	5.270458	-2.134435	-1.070822

TS$_{10a''-11a''}$

Center Number	Atomic Number	Atomic Type	Coordinates (Angstroms)		
			X	Y	Z
1	6	0	-4.731548	-0.849225	0.544570
2	1	0	-5.561535	-0.988106	1.220008
3	6	0	-4.654154	-0.980158	-0.806238
4	1	0	-5.403968	-1.254251	-1.532203
5	7	0	-3.470775	-0.470892	0.979110
6	7	0	-3.348160	-0.678768	-1.159630
7	6	0	-2.601329	-0.358894	-0.064832
8	29	0	-0.781682	0.198110	-0.019108
9	6	0	2.185127	-1.203237	0.013853
10	6	0	2.205909	0.250530	-0.209327
11	6	0	3.446221	0.779412	-0.607040
12	1	0	3.503238	1.838392	-0.811660
13	6	0	4.597376	-0.015161	-0.765547
14	1	0	5.506085	0.469257	-1.110800
15	6	0	4.608567	-1.370700	-0.491568
16	6	0	3.401611	-1.967546	-0.057251
17	1	0	3.332162	-3.047708	-0.007737
18	6	0	1.038417	2.311949	0.008504
19	8	0	1.993494	3.071531	-0.014519
20	6	0	3.064400	-1.407239	1.810176
21	1	0	2.071169	-1.232342	2.225020
22	1	0	3.484002	-2.339118	2.182182
23	1	0	3.731998	-0.558657	1.916826
24	7	0	1.029900	0.921323	-0.036464
25	8	0	0.967689	-1.763202	0.079962
26	6	0	0.807455	-3.194080	0.061566
27	1	0	1.245884	-3.623921	-0.843578
28	1	0	1.244753	-3.649616	0.955741
29	1	0	-0.269700	-3.354977	0.063629
30	6	0	-2.860257	-0.673753	-2.537731
31	1	0	-3.397620	0.075219	-3.125369
32	1	0	-1.798820	-0.425559	-2.527906
33	1	0	-2.999520	-1.659094	-2.990426
34	6	0	-3.140648	-0.200738	2.377152
35	1	0	-3.316296	-1.091954	2.985635
36	1	0	-2.088521	0.078313	2.435964
37	1	0	-3.750974	0.623552	2.755260
38	8	0	-0.243051	2.755662	0.114753
39	6	0	-0.403509	4.188597	0.190507
40	1	0	0.001376	4.663924	-0.705487
41	1	0	-1.478207	4.353057	0.261859
42	1	0	0.107499	4.581916	1.071985
43	1	0	5.502121	-1.975596	-0.588246

11a”

Center Number	Atomic Number	Atomic Type	Coordinates (Angstroms)		
			X	Y	Z
1	6	0	-4.698520	-1.125390	0.590079
2	1	0	-5.473239	-1.411632	1.284522
3	6	0	-4.708117	-1.042738	-0.766810
4	1	0	-5.492698	-1.243692	-1.479855
5	7	0	-3.429326	-0.749380	1.001099
6	7	0	-3.444823	-0.618003	-1.147216
7	6	0	-2.639673	-0.429278	-0.063254
8	29	0	-0.852683	0.221376	-0.036373
9	6	0	2.215568	-1.043098	-0.107893
10	6	0	2.137871	0.377116	-0.205262
11	6	0	3.346467	0.999736	-0.581803
12	1	0	3.312645	2.067014	-0.758101
13	6	0	4.592025	0.323797	-0.751863
14	1	0	5.453491	0.910110	-1.055243
15	6	0	4.697700	-1.008953	-0.535024
16	6	0	3.512603	-1.783654	-0.081960
17	1	0	3.425474	-2.698443	-0.684004
18	6	0	0.898511	2.371860	0.149188
19	8	0	1.840946	3.152753	0.215802
20	6	0	3.795266	-2.232906	1.407370
21	1	0	2.958236	-2.794092	1.825218
22	1	0	4.684145	-2.866930	1.423498
23	1	0	3.974303	-1.354830	2.031225
24	7	0	0.921470	1.004368	-0.029862
25	8	0	1.072675	-1.688481	0.040184
26	6	0	0.933930	-3.115079	-0.159429
27	1	0	1.309944	-3.392955	-1.147837
28	1	0	1.445820	-3.677823	0.621780
29	1	0	-0.138306	-3.295988	-0.105136
30	6	0	-3.052751	-0.375097	-2.534456
31	1	0	-3.676266	0.410291	-2.969566
32	1	0	-2.011613	-0.052329	-2.547490
33	1	0	-3.158573	-1.290758	-3.122427
34	6	0	-3.017253	-0.674789	2.401695
35	1	0	-3.137260	-1.649521	2.882116
36	1	0	-1.968889	-0.378295	2.438225
37	1	0	-3.618125	0.068136	2.932796
38	8	0	-0.392956	2.787540	0.273980
39	6	0	-0.578084	4.202605	0.486096
40	1	0	-0.169843	4.770780	-0.352661
41	1	0	-1.656478	4.342881	0.556411
42	1	0	-0.087263	4.517305	1.409642
43	1	0	5.642484	-1.537313	-0.616199

10h”

Center Number	Atomic Number	Atomic Type	Coordinates (Angstroms)		
			X	Y	Z
1	6	0	-5.303997	-1.838365	0.325639
2	1	0	-6.137597	-2.153424	0.934082
3	6	0	-5.073024	-1.979595	-1.006467
4	1	0	-5.667740	-2.439678	-1.780434
5	7	0	-4.211398	-1.164558	0.848599
6	7	0	-3.844761	-1.389789	-1.260740
7	6	0	-3.298776	-0.878488	-0.121759
8	29	0	-1.647297	0.062496	0.046968
9	6	0	1.422114	-0.413776	0.662092
10	6	0	1.096463	1.036931	0.279437
11	6	0	2.204877	1.937021	0.064769
12	1	0	2.010528	2.996919	-0.022467
13	6	0	3.463788	1.452529	-0.106959
14	1	0	4.284163	2.128899	-0.327241
15	6	0	3.773796	0.033918	-0.050173
16	6	0	2.806136	-0.852024	0.261832
17	1	0	3.036491	-1.909635	0.323896
18	6	0	-0.560752	2.730380	0.102121
19	8	0	-0.039064	3.661364	0.675244
20	6	0	1.360193	-0.454393	2.222545
21	1	0	0.346654	-0.196097	2.542492
22	1	0	1.618127	-1.449105	2.592661
23	1	0	2.062410	0.261219	2.657786
24	7	0	-0.173787	1.360400	0.215152
25	8	0	0.390334	-1.227364	0.085951
26	6	0	0.466760	-2.650113	0.246664
27	1	0	1.253772	-3.080487	-0.380826
28	1	0	0.621421	-2.939864	1.291099
29	1	0	-0.499323	-3.032625	-0.085135
30	6	0	-3.243466	-1.307708	-2.591324
31	1	0	-3.872015	-0.705700	-3.252797
32	1	0	-2.264085	-0.837249	-2.502973
33	1	0	-3.126696	-2.309301	-3.012998
34	6	0	-4.079864	-0.803493	2.259034
35	1	0	-4.057353	-1.703015	2.879930
36	1	0	-3.149650	-0.250404	2.389118
37	1	0	-4.917739	-0.171462	2.564169
38	8	0	-1.635415	2.817427	-0.690934
39	6	0	-2.182704	4.149454	-0.864850
40	1	0	-1.436571	4.803867	-1.319254
41	1	0	-3.038863	4.019916	-1.524509
42	1	0	-2.489393	4.555080	0.101127
43	6	0	5.186376	-0.367526	-0.356504
44	8	0	6.048245	0.437908	-0.647502
45	8	0	5.378458	-1.692353	-0.272645
46	6	0	6.721257	-2.157781	-0.556949
47	1	0	6.679951	-3.239084	-0.437035
48	1	0	7.002044	-1.887960	-1.576877
49	1	0	7.427161	-1.713253	0.147253

TS$_{10h''-11h''}$

Center Number	Atomic Number	Atomic Type	Coordinates (Angstroms)		
			X	Y	Z
1	6	0	-5.311220	-1.941427	0.477920
2	1	0	-6.089848	-2.292201	1.137498
3	6	0	-5.164910	-2.064086	-0.867974
4	1	0	-5.792726	-2.540524	-1.605006
5	7	0	-4.213061	-1.228426	0.932818
6	7	0	-3.980576	-1.423953	-1.198068
7	6	0	-3.378797	-0.898752	-0.093470
8	29	0	-1.779471	0.132014	-0.017122
9	6	0	1.447905	-0.395366	0.125184
10	6	0	1.080254	1.018767	-0.092531
11	6	0	2.153032	1.876734	-0.409624
12	1	0	1.931977	2.914967	-0.607254
13	6	0	3.477660	1.430846	-0.496006
14	1	0	4.255994	2.134901	-0.774758
15	6	0	3.847899	0.117151	-0.226962
16	6	0	2.822880	-0.798446	0.118838
17	1	0	3.062522	-1.853109	0.158246
18	6	0	-0.624986	2.675582	0.012058
19	8	0	0.077748	3.671262	0.023739
20	6	0	2.231902	-0.397802	1.960742
21	1	0	1.212484	-0.534273	2.322954
22	1	0	2.887815	-1.179665	2.338033
23	1	0	2.617378	0.603480	2.123113
24	7	0	-0.237031	1.333440	0.006697
25	8	0	0.427751	-1.266515	0.098771
26	6	0	0.671546	-2.686587	0.056771
27	1	0	1.266234	-2.952335	-0.821670
28	1	0	1.164353	-3.026561	0.972896
29	1	0	-0.316860	-3.138417	-0.012753
30	6	0	-3.478750	-1.299725	-2.565734
31	1	0	-4.173082	-0.710747	-3.170792
32	1	0	-2.513591	-0.793740	-2.536100
33	1	0	-3.357023	-2.289737	-3.012930
34	6	0	-4.003193	-0.866379	2.333353
35	1	0	-3.904312	-1.766091	2.946794
36	1	0	-3.089821	-0.275663	2.404417
37	1	0	-4.843848	-0.269679	2.696508
38	8	0	-1.981090	2.736030	0.030052
39	6	0	-2.547502	4.065321	0.056963
40	1	0	-2.239517	4.625931	-0.828062
41	1	0	-3.626566	3.916032	0.063453
42	1	0	-2.223816	4.596448	0.954576
43	6	0	5.272263	-0.292776	-0.293448
44	8	0	6.174531	0.442159	-0.640166
45	8	0	5.446708	-1.582978	0.078387
46	6	0	6.809579	-2.071201	0.030816
47	1	0	6.755375	-3.108584	0.357825
48	1	0	7.196130	-2.003251	-0.987999
49	1	0	7.441525	-1.483781	0.699788

11h”

Center Number	Atomic Number	Atomic Type	Coordinates (Angstroms)		
			X	Y	Z
1	6	0	-5.223610	-2.114571	0.644302
2	1	0	-5.863437	-2.641322	1.335312
3	6	0	-5.341607	-1.906933	-0.693986
4	1	0	-6.103877	-2.218244	-1.391415
5	7	0	-4.051743	-1.489338	1.040699
6	7	0	-4.238637	-1.160716	-1.078362
7	6	0	-3.429539	-0.891459	-0.014669
8	29	0	-1.844030	0.160596	-0.000554
9	6	0	1.415006	-0.350779	-0.131495
10	6	0	1.037461	1.027842	-0.010971
11	6	0	2.129793	1.921695	-0.010656
12	1	0	1.897866	2.978449	0.000093
13	6	0	3.494982	1.525837	-0.068716
14	1	0	4.259561	2.294451	-0.123252
15	6	0	3.867213	0.216450	-0.066665
16	6	0	2.812476	-0.829321	0.070176
17	1	0	3.030928	-1.668579	-0.597932
18	6	0	-0.684132	2.675180	0.078349
19	8	0	0.013312	3.680109	0.112188
20	6	0	2.868736	-1.392357	1.554465
21	1	0	2.070273	-2.113390	1.739377
22	1	0	3.831863	-1.884134	1.692972
23	1	0	2.771826	-0.569570	2.265705
24	7	0	-0.299159	1.347314	0.013936
25	8	0	0.440109	-1.212356	-0.322946
26	6	0	0.661719	-2.590863	-0.715998
27	1	0	1.217416	-2.621230	-1.656602
28	1	0	1.185182	-3.145185	0.063262
29	1	0	-0.337931	-2.997904	-0.856591
30	6	0	-4.006943	-0.698886	-2.446176
31	1	0	-4.819349	-0.039953	-2.763466
32	1	0	-3.068474	-0.144836	-2.470335
33	1	0	-3.944306	-1.552109	-3.126845
34	6	0	-3.577319	-1.453775	2.422814
35	1	0	-3.418726	-2.469925	2.793802
36	1	0	-2.635777	-0.905068	2.450593
37	1	0	-4.306647	-0.944747	3.058355
38	8	0	-2.044271	2.740466	0.103484
39	6	0	-2.606838	4.067858	0.181909
40	1	0	-2.311537	4.658077	-0.688276
41	1	0	-3.686274	3.920359	0.199536
42	1	0	-2.270888	4.569941	1.091685
43	6	0	5.309182	-0.142650	-0.150379
44	8	0	6.208147	0.672708	-0.192720
45	8	0	5.496243	-1.480545	-0.193357
46	6	0	6.874079	-1.920001	-0.296217
47	1	0	6.828323	-3.007741	-0.314940
48	1	0	7.320932	-1.531751	-1.213491
49	1	0	7.444438	-1.566371	0.564907

4. Analytical data

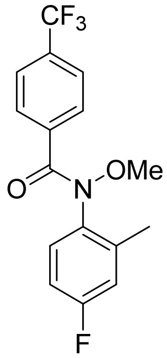

***N*-(4-Fluoro-2-methylphenyl)-*N*-methoxy-4-(trifluoromethyl)benzamide (1b).** Brown solid. Rf = 0.44 [hexane/EtOAc = 3:1 (v/v)]. ^{1}H NMR (400 MHz, $CDCl_3$) δ 7.73 (br, 2H), 7.63 (br, 2H), 7.23 (br, 1H), 7.01 (d, *J* = 8.7 Hz, 1H), 6.92 (br, 1H), 3.66 (s, 3H), 2.36 (s, 3H). ^{13}C NMR (100 MHz, $CDCl_3$) δ 167.1, 162.4 (d, *J* = 249 Hz), 139.3 (d, *J* = 8.6 Hz), 137.6, 132.1 (q, *J* = 33 Hz), 130.1, 128.4, 128.1, 127.5, 124.8 (q, *J* = 2.9 Hz), 122.1, 119.4, 117.8 (d, *J* = 22 Hz), 113.4 (d, *J* = 23 Hz), 60.7, 17.8. ^{19}F NMR (565 MHz, $CDCl_3$) δ -111.8, -110.4, -62.9. IR (neat) 3068, 2973, 2937, 2817, 1926, 1768, 1664, 1618, 1588, 1493, 1439, 1409, 1379, 1271, 1246, 1228, 1215, 1168,1125, 1109, 1065, 1038, 1016, 972, 951, 886, 856, 836, 815, 762, 714, 686 cm^{-1}. HRMS (ESI) calcd. for $C_{16}H_{13}F_4NO_2$ $(M+Na)^+$ 327.0882 , found 327.0880.

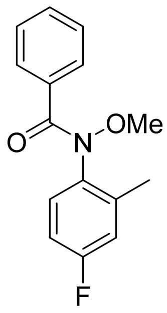

***N*-(4-Fluoro-2-methylphenyl)-*N*-methoxybenzamide (1c).** Orange oil. Rf = 0.27 [hexane/EtOAc = 3:1 (v/v)]. ^{1}H NMR (400 MHz, $CDCl_3$) δ 7.56 (m, 2H), 7.33-7.40 (m, 3H), 7.21 (br, 1H), 6.98 (d, *J* = 8.7 Hz, 1H), 6.88 (br, 1H), 3.71 (s, 3H), 2.35 (s, 3H). ^{13}C NMR (100 MHz, $CDCl_3$) δ 168.2, 162.0 (d, *J* = 249 Hz), 139.1 (d, *J* = 8.6 Hz), 133.8, 133.5, 130.4, 130.2, 127.9, 127.7, 117.5 (d, *J* = 22 Hz), 113.0 (d, *J* = 23 Hz), 60.4, 17.8. ^{19}F NMR (565 MHz, $CDCl_3$) δ -112.0. IR (neat) 3062, 3003, 2973, 2933, 2897, 2815, 1959, 1889, 1814, 1773, 1659, 1617, 1601, 1588, 1493, 1446, 1417, 1352, 1270, 1244, 1227, 1182, 1151, 1101, 1073, 1040, 1022, 971, 950, 865, 838, 814, 785, 754, 696, 679 cm^{-1}. HRMS (ESI) calcd. for $C_{15}H_{14}FNO_2$ $(M+Na)^+$ 259.1009, found 259.1009.

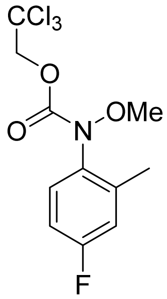

2,2,2-Trichloroethyl (4-fluoro-2-methylphenyl)(methoxy)carbamate (1e). Orange oil. Rf = 0.58 [hexane/EtOAc = 3:1 (v/v)]. ^{1}H NMR (400 MHz, $CDCl_3$) δ 7.28 (dd, *J* = 8.7, 5.5 Hz, 1H), 6.99 (dd, *J* = 9.2, 2.8 Hz, 1H), 6.93 (td, *J* = 8.2, 2.8 Hz, 1H), 4.82 (s, 2H), 3.78 (s, 3H), 2.33 (s, 3H). ^{13}C NMR (100 MHz, $CDCl_3$) δ 162.4 (d, *J* = 249 Hz), 153.2, 139.2 (d, *J* = 9.6 Hz), 133.1 (d, *J* = 2.9 Hz), 129.8 (d, *J* = 9.6 Hz), 117.5 (d, *J* = 22 Hz), 95.0, 74.9, 61.7, 17.8. ^{19}F NMR (565 MHz, $CDCl_3$) δ -111.9 IR (neat) 2966, 2935, 1747, 1275, 1616, 1589, 1496, 1438, 1421, 1387, 1334, 1269, 1240, 1225, 1151, 1111, 1056, 985, 958, 938, 905, 866, 822, 778, 743, 712 cm^{-1}. HRMS (ESI) calcd. for $C_{11}H_{11}Cl_3FNO_3$ $(M+Na)^+$ 328.9789, found 328.9787.

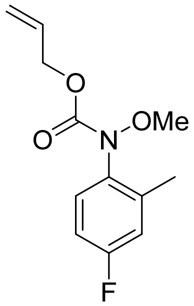

Allyl (4-fluoro-2-methylphenyl)(methoxy)carbamate (1f). Orange oil. Rf = 0.50 [hexane/EtOAc = 3:1 (v/v)]. ^{1}H NMR (400 MHz, $CDCl_3$) δ 7.25 (dd, *J* = 8.7, 5.5 Hz, 1H), 6.97 (dd, *J* = 9.2, 2.8 Hz, 1H), 6.91 (td, *J* = 8.7, 2.8 Hz, 1H), 5.92 (ddt, *J* = 16.9, 10.5, 5.5 Hz, 1H), 5.26 (d, *J* = 16.9 Hz, 1H), 5.22 (dq, *J* = 10.5, 0.9 Hz, 1H), 4.67 (dt, *J* = 6.0, 1.4 Hz, 2H), 3.73 (s, 3H), 2.29 (s, 3H). ^{13}C NMR (100 MHz, $CDCl_3$) δ 162.2 (d, *J* = 248 Hz), 154.9, 139.0 (d, *J* = 8.6 Hz), 134.1, 131.9, 129.5 (d, *J* = 8.6 Hz), 118.0, 117.4 (d, *J* = 22 Hz), 113.1 (d, *J* = 22 Hz), 66.6, 61.7, 17.7. ^{19}F NMR (565 MHz, $CDCl_3$) δ -112.7. IR (neat) 3087, 2971, 2935, 2897, 2816, 1735, 1714, 1649, 1616, 1589, 1496, 1441, 1419, 1381, 1325, 1269, 1244 1224, 1188, 1150, 1091, 1030, 989, 935, 865, 820, 771, 729 cm^{-1}. HRMS (ESI) calcd. for $C_{12}H_{14}FNO_3$ $(M+Na)^+$ 239.0958, found 239.0957.

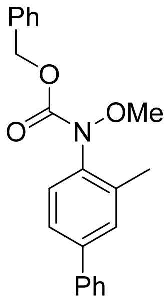

Benzyl methoxy[3-methyl-(1,1'-biphenyl)-4-yl]carbamate (1h). Yellow oil. Rf = 0.50 [hexane/EtOAc = 3:1 (v/v)]. ^{1}H NMR (600 MHz, $CDCl_3$) δ 7.57-7.59 (m, 2H), 7.42-7.47 (m, 4H), 7.29-7.37 (m, 7H), 5.25 (s, 2H), 3.77 (s, 3H), 2.31 (s, 3H). ^{13}C NMR (100 MHz, $CDCl_3$) δ 155.2, 141.7, 140.3, 137.2, 136.6, 136.0, 129.7, 128.7, 128.4, 128.1, 127.9, 127.5, 127.1, 125.2, 67.7, 61.9, 17.9. IR (neat) 3061, 3032, 2965, 2931, 2894, 1954, 1891, 1727, 1601, 1586, 1571, 1508, 1486, 1455, 1388, 1327, 1239, 1180, 1131, 1091, 1024, 986, 907, 885, 832, 768, 734, 714, 695 cm^{-1}. HRMS (ESI) calcd. for $C_{22}H_{21}NO_3$ $(M+Na)^+$ 347.1521, found 347.1521.

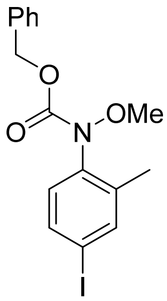

Benzyl (4-iodo-2-methylphenyl)(methoxy)carbamate (1k). Yellow oil. Rf = 0.53 [hexane/EtOAc = 3:1 (v/v)]. ^{1}H NMR (600 MHz, $CDCl_3$) δ 7.61 (dd, J = 2.1, 0.7 Hz, 1H), 7.54 (ddd, J = 8.3, 2.1, 0.7 Hz, 1H), 7.30-7.36 (m, 5H), 7.00 (d, J = 8.3 Hz, 1H), 5.21 (s, 2H), 3.71 (s, 3H), 2.18 (s, 3H). ^{13}C NMR (100 MHz, $CDCl_3$) δ 154.8, 139.7, 138.6, 138.0, 135.7, 135.5, 129.2, 128.4, 128.1, 127.9, 94.4, 67.8, 62.0, 17.4. IR (neat) 3089, 3064, 3033, 2964, 2931, 2893, 2813, 1953, 1711, 1586, 1479, 1455, 1393, 1322, 1237, 1190, 1131, 1091, 1022, 984, 934, 907, 873, 820, 696 cm^{-1}. HRMS (ESI) calcd. for $C_{16}H_{16}INO_3$ $(M+Na)^+$ 397.0175, found 397.0175.

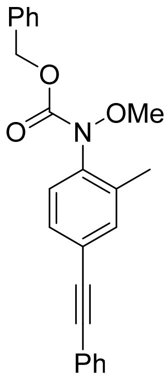

Benzyl methoxy[2-methyl-4-(phenylethynyl)phenyl]carbamate (1l). Yellow oil. Rf = 0.53 [hexane/EtOAc = 3:1 (v/v)]. ^{1}H NMR (600 MHz, $CDCl_3$) δ 7.51-7.54 (m, 2H), 7.43 (br, 1H), 7.30-7.39 (m, 9H), 7.26 (d, *J* = 8.3 Hz, 1H), 5.23 (s, 2H), 3.74 (s, 3H), 2.24 (s, 3H). ^{13}C NMR (100 MHz, $CDCl_3$) δ 154.9, 137.9, 136.4, 135.8, 134.0, 131.5, 129.6, 128.4, 128.4, 128.3, 128.1, 127.9, 127.6, 123.6, 122.9, 90.1, 88.6, 67.8, 62.0, 17.6. IR (neat) 3064, 3033, 2967, 2932, 2894, 2251, 1714, 1602, 1572, 1498, 1455, 1442, 1388, 1335, 1309, 1238, 1092, 1023, 986, 907, 829, 755, 728, 690 cm^{-1}. HRMS (ESI) calcd. for $C_{24}H_{21}NO_3$ $(M+Na)^+$ 371.1521, found 371.1521.

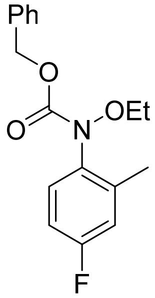

Benzyl ethoxy(4-fluoro-2-methylphenyl)carbamate (1n). Yellow oil. Rf = 0.56 [hexane/EtOAc = 3:1 (v/v)]. ^{1}H NMR (600 MHz, $CDCl_3$) δ 7.29-7.35 (m, 5H), 7.24-7.26 (m, 1H), 6.94 (dd, *J* = 9.3, 2.8 Hz, 1H), 6.90 (dt, *J* = 8.3, 3.1 Hz, 1H), 5.21 (s, 2H), 3.95 (q, *J* = 6.9 Hz, 2H), 2.23 (s, 3H), 1.22 (t, *J* = 7.2 Hz, 3H). ^{13}C NMR (100 MHz, $CDCl_3$) δ 162.1 (d, *J* = 248 Hz), 155.5, 138.9, 136.0, 134.9, 129.4 (d, *J* = 9.6 Hz), 128.5, 128.1, 127.9, 117.4 (d, *J* = 22 Hz), 113.2 (d, *J* = 22 Hz), 69.9, 67.8, 17.9, 13.5. ^{19}F NMR (565 MHz, $CDCl_3$) δ -113.1. IR (neat) 3066, 3034, 2981, 2935, 2888, 1955, 1869, 1733, 1714, 1617, 1589, 1496, 1456, 1418, 1389, 1331, 1268,1244, 1150, 1091, 1031, 955, 939, 868, 819, 795, 755 cm^{-1}. HRMS (ESI) calcd. for $C_{17}H_{18}FNO_3$ $(M+Na)^+$ 303.12707, found 303.12705.

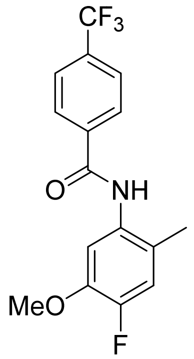

N-(4-Fluoro-5-methoxy-2-methylphenyl)-4-(trifluoromethyl)benzamide (3ba). Colorless solid. Rf = 0.42 [hexane/EtOAc = 3:1 (v/v)]. ^{1}H NMR (400 MHz, CDCl3) δ 8.00 (d, *J* = 8.2 Hz, 2H), 7.78 (d, *J* = 8.2 Hz, 2H), 7.69 (d, *J* = 7.8 Hz, 1H), 7.59 (br, 1H), 6.97 (d, *J* = 11.5 Hz, 1H), 3.91 (s, 3H), 2.26 (s, 3H). ^{13}C NMR (100 MHz, CDCl3) δ 164.4, 149.7 (d, *J* = 244 Hz), 145.8 (d, *J* = 12 Hz), 137.9, 133.7 (q, *J* = 33 Hz), 131.0 (d, *J* = 2.9 Hz), 127.5, 126.0 (q, *J* = 3.8 Hz), 123.5 (q, *J* = 272 Hz), 121.9 (d, *J* = 5.8 Hz), 117.6 (d, *J* = 19 Hz), 109.2, 56.4, 16.9. ^{19}F NMR (565 MHz, CDCl3) δ -138.2, -62.9. IR (neat) 3271, 2924, 2853, 1662, 1638, 1536, 1519, 1469, 1408, 1330, 1277, 1218, 1199, 1170, 1127, 1109, 1091, 1067, 1017, 925, 876, 858, 691 cm^{-1}. HRMS (ESI) calcd. for $C_{16}H_{13}F_4NO_2$ $(M+Na)^+$ 327.0882, found 327.0882.

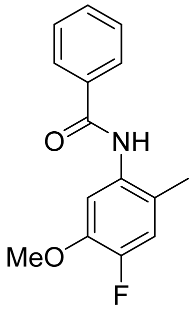

N-(4-Fluoro-5-methoxy-2-methylphenyl)benzamide (3ca). Colorless solid. Rf = 0.22 [hexane/EtOAc = 3:1 (v/v)]. ^{1}H NMR (400 MHz, CDCl3) δ 7.85-7.90 (m, 2H), 7.64-7.73 (m, 2H), 7.54-7.60 (m, 1H), 7.47-7.53 (m, 2H), 6.93 (d, *J* = 11.5 Hz, 1H), 3.88 (s, 3H), 2.24 (s, 3H). ^{13}C NMR (100 MHz, CDCl3) δ 165.7, 149.4 (d, *J* = 243 Hz), 145.6 (d, *J* = 11 Hz), 134.5, 131.9, 131.5 (d, *J* = 2.9 Hz), 128.8, 127.0, 122.0 (d, *J* = 6.7 Hz), 117.4 (d, *J* = 19 Hz), 109.2, 56.3, 16.9. ^{19}F NMR (565 MHz, CDCl3) δ -139.1. IR (neat) 3286, 2960, 2933, 1644, 1602, 1579, 1510, 1487, 1462, 1408, 1334, 1308, 1272, 1212, 1199, 1132, 1092, 1074, 1017, 918, 864, 849, 836, 798, 771, 753, 709, 691, 665 cm^{-1}. HRMS (ESI) calcd. for $C_{15}H_{14}FNO_2$ $(M+Na)^+$ 259.1009, found 259.1008.

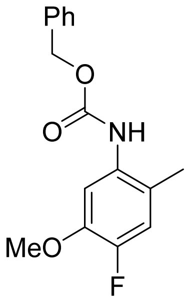

Benzyl (4-fluoro-5-methoxy-2-methylphenyl)carbamate (3da). Colorless solid. Rf = 0.39 [hexane/EtOAc = 3:1 (v/v)]. ^{1}H NMR (400 MHz, $CDCl_3$) δ 7.56 (br, 1H), 7.37-7.41 (m, 5H), 6.86 (d, *J* = 11.5 Hz, 1H), 6.39 (br, 1H), 5.20 (s, 2H), 3.86 (s, 3H), 2.14 (s, 3H). ^{13}C NMR (100 MHz, $CDCl_3$) δ 153.7, 148.7 (d, *J* = 241 Hz), 145.7 (d, *J* = 11 Hz), 135.9, 131.5, 128.6, 128.4, 128.4, 119.5, 117.3 (d, *J* = 19 Hz), 107.1, 67.2, 56.3, 16.8. ^{19}F NMR (565 MHz, $CDCl_3$) δ -141.0. IR (neat) 3279, 2956, 2928, 1692, 1535, 1514, 1454, 1409, 1334, 1254, 1207, 1121, 1056, 1019, 873, 837, 772, 694 cm^{-1}. HRMS (ESI) calcd. for $C_{16}H_{16}FNO_3$ $(M+Na)^+$ 289.1114, found 289.1113.

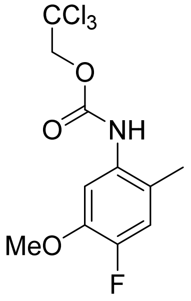

2,2,2-Trichloroethyl (4-fluoro-5-methoxy-2-methylphenyl)carbamate (3ea). Colorless solid. Rf = 0.49 [hexane/EtOAc = 3:1 (v/v)]. ^{1}H NMR (400 MHz, $CDCl_3$) δ 7.45 (d, *J* = 6.0 Hz, 1H), 6.91 (d, *J* = 11.5 Hz, 1H), 6.57 (br, 1H), 4.83 (s, 2H), 3.88 (s, 3H), 2.22 (s, 3H). ^{13}C NMR (100 MHz, $CDCl_3$) δ 152.1, 149.3 (d, *J* = 244 Hz), 145.9, 130.6 121.0, 117.5 (d, *J* = 19 Hz), 108.0, 95.2, 74.6, 56.4, 16.8. ^{19}F NMR (565 MHz, $CDCl_3$) δ -139.5. IR (neat) 3317, 2957, 1724, 1629, 1601, 1534, 1457, 1413, 1333, 1276, 1126, 1092, 1048, 1016, 966, 871, 820, 741, 719 cm^{-1}. HRMS (ESI) calcd. for $C_{11}H_{11}Cl_3FNO_3$ $(M+Na)^+$ 328.9789, found 328.9788.

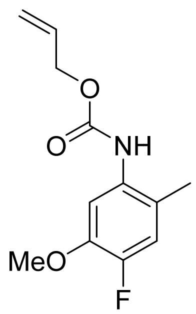

Allyl (4-fluoro-5-methoxy-2-methylphenyl)carbamate (3fa). Colorless solid. Rf = 0.39 [hexane/EtOAc = 3:1 (v/v)]. ^{1}H NMR (400 MHz, $CDCl_3$) δ 7.55 (br, 1H), 6.88 (d, *J* = 11.5 Hz, 1H), 6.35 (br, 1H), 5.98 (ddt, *J* = 17.0, 11.0, 5.5 Hz, 1H), 5.38 (d, *J* = 17.4 Hz, 1H), 5.28 (d, *J* = 11.5 Hz, 1H), 4.67 (d, *J* = 5.5 Hz, 2H), 3.88 (s, 3H), 2.17 (s, 3H). ^{13}C NMR (100 MHz, $CDCl_3$) δ 153.6, 148.7 (d, *J* = 244 Hz), 145.6 (d, *J* = 12 Hz), 132.3, 131.4, 112.0, 118.2, 117.2 (d, *J* = 19 Hz), 107.3, 65.9, 56.2, 16.6. ^{19}F NMR (565 MHz, $CDCl_3$) δ -141.0. IR (neat) 3317, 2937, 1708, 1649, 1628, 1604, 1530, 1455, 1412, 1331, 1228, 1200, 1120, 1047, 996, 933, 870, 733, 699 cm^{-1}. HRMS (ESI) calcd. for $C_{12}H_{14}FNO_3$ (M+Na)$^+$ 239.0958, found 239.0957.

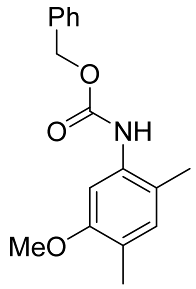

Benzyl (5-methoxy-2,4-dimethylphenyl)carbamate (3ga). Colorless solid. Rf = 0.54 [hexane/EtOAc = 3:1 (v/v)]. ^{1}H NMR (400 MHz, $CDCl_3$) δ 7.32-7.46 (m, 6H), 6.89 (s, 1H), 6.42 (br, 1H), 5.20 (s, 2H), 3.81 (s, 3H), 2.14 (s, 3H), 2.13 (s, 3H). ^{13}C NMR (100 MHz, $CDCl_3$) δ 156.4, 153.9, 136.3, 134.1, 132.2, 128.7, 128.5, 122.2, 118.5, 103.8, 67.1, 56.6, 16.7, 15.7. IR (neat) 3303, 2922, 1692, 1661, 1621, 1594, 1532, 1505, 1463, 1402, 1371, 1317, 1291, 1272, 1241, 1208, 1126, 1055, 1011, 988, 920, 873, 835, 747, 737, 697, 663 cm^{-1}. HRMS (ESI) calcd. for $C_{17}H_{19}NO_3$ (M+Na)$^+$ 285.1365, found 285.1364.

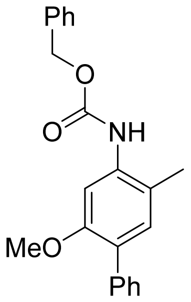

Benzyl (2-methoxy-5-methyl-[1,1'-biphenyl]-4-yl)carbamate (3ha). Colorless solid. Rf = 0.48 [hexane/EtOAc = 3:1 (v/v)]. ^{1}H NMR (400 MHz, $CDCl_3$) δ 7.69 (br, 1H), 7.27-7.51 (m, 10H), 7.09 (s, 1H), 6.54 (br, 1H), 5.24 (s, 2H), 3.81 (s, 3H), 2.21 (s, 3H). ^{13}C NMR (100 MHz, $CDCl_3$) δ 155.2, 153.5, 138.1, 135.9, 135.9, 132.3, 129.4, 128.7, 128.5, 128.4, 128.0, 126.7, 126.0, 67.2, 55.7, 16.7. IR (neat) 3335, 2925, 2854, 1697, 1591, 1533, 1507, 1487, 1462, 1397, 1313, 1243, 1148, 1063, 1042, 892, 839, 697 cm^{-1}. HRMS (ESI) calcd. for $C_{22}H_{21}NO_3$ $(M+Na)^+$ 347.1521, found 347.1521.

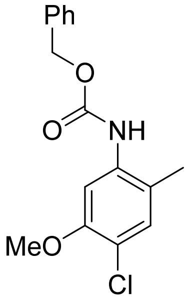

Benzyl (4-chloro-5-methoxy-2-methylphenyl)carbamate (3ia). Colorless solid. Rf = 0.43 [hexane/EtOAc = 3:1 (v/v)]. ^{1}H NMR (600 MHz, $CDCl_3$) δ 7.69 (br, 1H), 7.36-7.43 (m, 5H), 7.12 (s, 1H), 6.47 (br, 1H), 5.21 (s, 2H), 3.89 (s, 3H), 2.14 (s, 3H). ^{13}C NMR (100 MHz, $CDCl_3$) δ 153.6, 153.3, 135.8, 135.3, 131.2, 128.7, 128.5, 128.4, 119.1, 109.1, 104.8, 67.3, 56.2, 16.5. IR (neat) 3295, 3033, 2956, 1705, 1612, 1584, 1523, 1454, 1397, 1301, 1219, 1073, 1046, 1003, 915, 882, 841, 772, 697 cm^{-1}. HRMS (ESI) calcd. for $C_{16}H_{16}ClNO_3$ $(M+Na)^+$ 305.0819, found 305.0818.

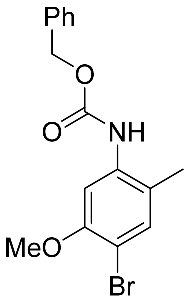

Benzyl (4-bromo-5-methoxy-2-methylphenyl)carbamate (3ja). Colorless solid. Rf = 0.42 [hexane/EtOAc = 3:1 (v/v)]. ^{1}H NMR (400 MHz, $CDCl_3$) δ 7.67 (br, 1H), 7.33-7.45 (m, 5H), 7.29 (s, 1H), 6.49 (br, 1H), 5.21 (s, 2H), 3.88 (s, 3H), 2.14 (s, 3H). ^{13}C NMR (100 MHz, $CDCl_3$) δ 154.5, 153.3, 136.0, 135.7, 134.1, 128.7, 128.35, 128.43, 119.5, 105.2, 104.4, 67.3, 56.3, 16.5. IR (neat) 3280, 3034, 2972, 2953, 2851, 1692, 1607, 1577, 1526, 1489, 1454, 1390, 1380, 1294, 1266, 1242, 1219, 1210, 1187, 1066, 1047, 736, 696 cm^{-1}. HRMS (ESI) calcd. for $C_{16}H_{16}BrNO_3$ $(M+Na)^+$ 349.0314, found 349.0313.

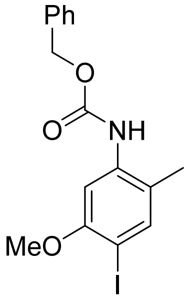

Benzyl (4-iodo-5-methoxy-2-methylphenyl)carbamate (3ka). Colorless solid. Rf = 0.50 [hexane/EtOAc = 3:1 (v/v)]. ^{1}H NMR (600 MHz, $CDCl_3$) δ 7.62 (br, 1H), 7.51 (s, 1H), 7.35-7.43 (m, 5H), 6.48 (br, 1H), 5.21 (s, 2H), 3.87 (s, 3H), 2.13 (s, 3H). ^{13}C NMR (100 MHz, $CDCl_3$) δ 157.1, 153.4, 140.1, 137.3, 135.9, 128.8, 128.6, 128.5, 120.3, 110.0, 103.5, 67.4, 56.5, 16.4. IR (neat) 3288, 2956, 1694, 1604, 1577, 1525, 1489, 1455, 1389, 1379, 1287, 1269, 1245, 1219, 1186, 1163, 1065, 1046, 1001, 882, 838, 697, 672 cm^{-1}. HRMS (ESI) calcd. for $C_{16}H_{16}INO_3$ $(M+Na)^+$ 397.0175, found 397.0174.

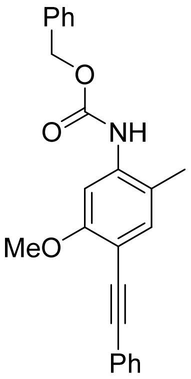

Benzyl (5-methoxy-2-methyl-4-(phenylethynyl)phenyl)carbamate (3la). Colorless oil. Rf = 0.60 [hexane/EtOAc = 3:1 (v/v)]. ^{1}H NMR (600 MHz, $CDCl_3$) δ 7.72 (br, 1H), 7.51-7.54 (m, 2H), 7.26-7.44 (m, 10H), 6.58 (s, 1H), 5.22 (s, 2H), 3.91 (s, 3H), 2.16 (s, 3H). ^{13}C NMR (100 MHz, $CDCl_3$) δ 159.1, 153.1, 137.2, 135.7, 131.6, 128.7, 128.5, 128.4, 128.2, 127.9, 123.7, 117.3, 107.0, 102.4, 92.6, 85.7, 67.3, 56.0, 16.5. IR (neat) 3331, 3033, 2930, 2856, 1737, 1613, 1583, 1518, 1486, 1456, 1403, 1335, 1259, 1219, 1110, 1044, 1004, 886, 852, 771, 692 cm^{-1}. HRMS (ESI) calcd. for $C_{24}H_{21}NO_3$ $(M+Na)^+$ 371.1521, found 371.1521.

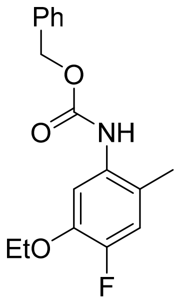

Benzyl (5-ethoxy-4-fluoro-2-methylphenyl)carbamate (3nb). Colorless solid. Rf = 0.45 [hexane/EtOAc = 3:1 (v/v)]. ^{1}H NMR (400 MHz, $CDCl_3$) δ 7.54 (br, 1H), 7.33-7.44 (m, 5H), 6.86 (d, *J* = 11.5 Hz, 1H), 6.36 (br, 1H), 5.20 (s, 2H), 4.09 (q, *J* = 6.9 Hz, 2H), 2.15 (s, 3H), 1.43 (t, *J* = 7.3 Hz, 3H). ^{13}C NMR (100 MHz, $CDCl_3$) δ 153.7, 147.8, 145.0 (d, *J* = 12 Hz), 135.9, 131.4, 128.6, 128.4, 128.3, 117.3 (d, *J* = 19 Hz), 108.4 (d, *J* = 20 Hz), 97.7, 67.2, 65.0, 16.8, 14.7. ^{19}F NMR (565 MHz, $CDCl_3$) δ -140.3. IR (neat) 3293, 2977, 2957, 2925, 1693, 1627, 1541, 1516, 1473, 1454, 1415, 1392, 1335, 1254, 1241, 1126, 1058, 1042, 1005, 924, 876, 845, 826, 694, 672 cm^{-1}. HRMS (ESI) calcd. for $C_{17}H_{18}FNO_3$ $(M+Na)^+$ 303.1271, found 303.1270.

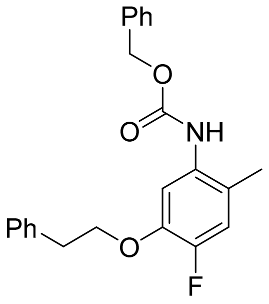

Benzyl (4-fluoro-2-methyl-5-phenethoxyphenyl)carbamate (3dc). Colorless solid. Rf = 0.41 [hexane/EtOAc = 3:1 (v/v)]. ^{1}H NMR (400 MHz, $CDCl_3$) δ 7.56 (br, 1H), 7.21-7.39 (m, 12H), 6.86 (d, J = 11.5 Hz, 1H), 6.35 (br, 1H), 5.18 (s, 2H), 4.21 (t, J = 6.4 Hz, 2H), 3.12 (t, J = 7.3 Hz, 2H), 2.14 (s, 3H). ^{13}C NMR (100 MHz, $CDCl_3$) δ 153.7, 149.0 (d, J = 241 Hz), 144.9 (d, J = 12 Hz), 137.8, 135.9, 131.5, 129.1, 128.6, 128.5, 128.4, 128.4, 126.5, 119.8, 117.4 (d, J = 19 Hz), 108.4, 70.1, 67.2, 35.7, 16.8. ^{19}F NMR (565 MHz, $CDCl_3$) δ -140.0. IR (neat) 3426, 3311, 3087, 3063, 3031, 2951, 2926, 2874, 1955, 1870, 1705, 1627, 1603, 1530, 1496, 1469, 1453, 1418, 1331, 1219, 1197, 1120, 1084, 1042, 1028, 872, 771, 749, 697 cm^{-1}. HRMS (ESI) calcd. for $C_{23}H_{22}FNO_3$ $(M+Na)^+$ 379.1584, found 379.1583.

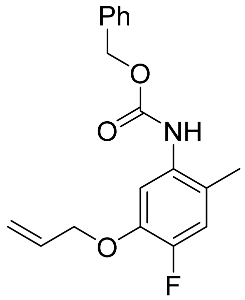

Benzyl (5-(allyloxy)-4-fluoro-2-methylphenyl)carbamate (3dd). Colorless solid. Rf = 0.46 [hexane/EtOAc = 3:1 (v/v)]. ^{1}H NMR (600 MHz, $CDCl_3$) δ 7.57 (br, 1H), 7.34-7.42 (m, 5H), 6.87 (d, J = 11.7 Hz, 1H), 6.37 (br, 1H), 6.05 (ddt, J = 16.2, 10.7, 5.5 Hz, 1H), 5.42 (d, J = 17.2 Hz, 1H), 5.29 (d, J = 11.3 Hz, 1H), 5.20 (s, 2H), 4.58 (s, 2H), 2.14 (s, 3H). ^{13}C NMR (100 MHz, $CDCl_3$) δ 153.7, 149.0 (d, J = 243 Hz), 144.6 (d, J = 11 Hz), 135.9, 132.7, 132.7, 132.7, 132.7, 131.4, 128.6, 128.4, 128.4, 118.3, 117.5 (d, J = 19 Hz), 113.3 (d, J = 22 Hz), 108.9, 70.3, 67.2, 16.8. ^{19}F NMR (565 MHz, $CDCl_3$) δ -139.8. IR (neat) 3283, 3033, 2958, 2925, 2863, 1692, 1649, 1627, 1540, 1516, 1455, 1415, 1337, 1256, 1220, 1124, 1056, 1017, 934, 876, 836, 695 cm^{-1}. HRMS (ESI) calcd. for $C_{18}H_{18}FNO_3$ $(M+Na)^+$ 315.1271, found 315.1271.

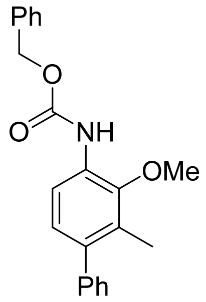

Benzyl (3-methoxy-2-methyl-[1,1'-biphenyl]-4-yl)carbamate (5h). Orange oil. Rf = 0.56 [hexane/EtOAc = 3:1 (v/v)]. ^{1}H NMR (400 MHz, $CDCl_3$) δ 8.00 (d, *J* = 7.7 Hz, 1H), 7.45-7.27 (m, 11H), 7.02 (d, *J* = 8.2 Hz, 1H), 5.24 (s, 2H), 3.76 (s, 3H), 2.19 (s, 3H). ^{13}C NMR (100 MHz, $CDCl_3$) δ 153.3, 146.9, 141.3, 138.0, 136.1, 130.4, 129.4, 128.6, 128.4, 128.3, 128.1, 126.8, 125.9, 116.0, 67.1, 60.3, 13.8. IR (neat) 3423, 3332, 3244, 3219, 3060, 3031, 2956, 2863, 2830, 1735, 1609, 1584, 1574, 1522, 1496, 1453, 1401, 1371, 1307, 1264, 1222, 1199, 1113, 1088, 1007, 950, 916, 827, 794, 770, 701, 677 cm^{-1}. HRMS (FD) calcd. for $C_{22}H_{21}NO_3$ (M^+) 347.15214, found 347.15212.

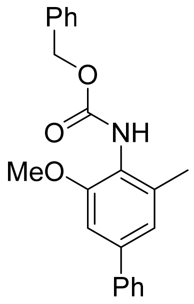

Benzyl (3-methoxy-5-methyl-[1,1'-biphenyl]-4-yl)carbamate (6h). Colorless solid. Rf = 0.25 [hexane/EtOAc = 3:1 (v/v)]. ^{1}H NMR (400 MHz, $CDCl_3$) δ 7.57-7.54 (m, 2H), 7.44-7.28 (m, 8H), 7.06 (s, 1H), 6.93 (s, 1H), 6.31 (br, 1H), 5.21 (s, 2H), 3.85 (s, 3H), 2.34 (s, 3H). ^{13}C NMR (100 MHz, $CDCl_3$) δ 154.2, 141.0, 140.3, 136.7, 136.4, 129.5, 128.7, 128.5, 128.2, 128.1, 127.9, 127.3, 127.1, 123.5, 121.7, 107.4, 67.1, 55.7, 18.5. IR (neat) 3384, 3282, 3060, 3032, 2955, 2933, 2853, 1712, 1591, 1575, 1520, 1494, 1455, 1404, 1341, 1289, 1218, 1148, 1099, 1080, 1048, 1029, 1004, 892, 848, 794, 764, 697 cm^{-1}. HRMS (FD) calcd. for $C_{22}H_{21}NO_3$ (M^+) 347.15214, found 347.15210.

5. NMR charts

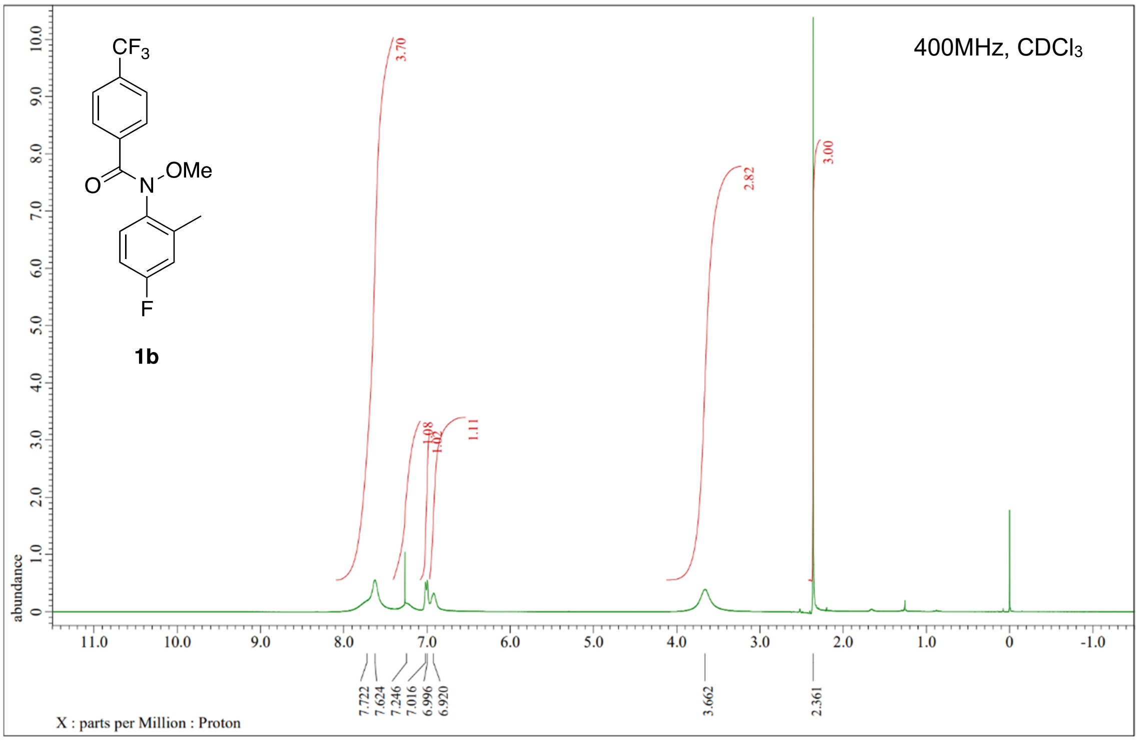

400MHz, CDCl3
1b
OMe
X : parts per Million : Proton

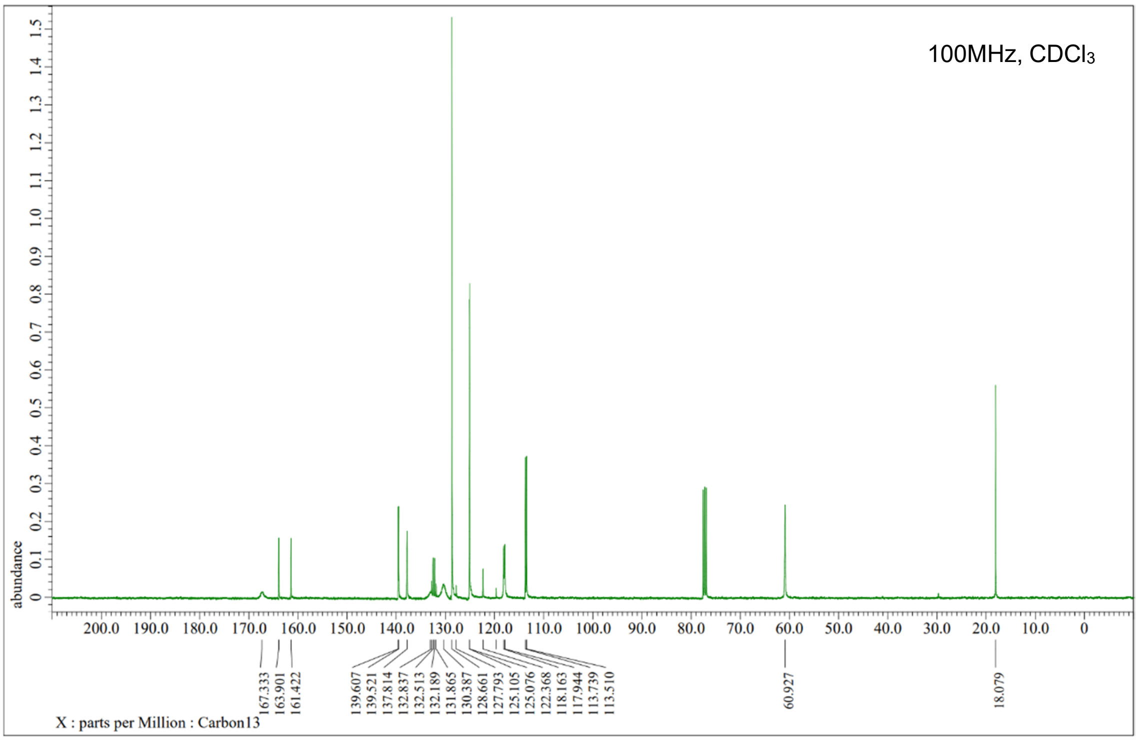

100MHz, CDCl3
X : parts per Million : Carbon13

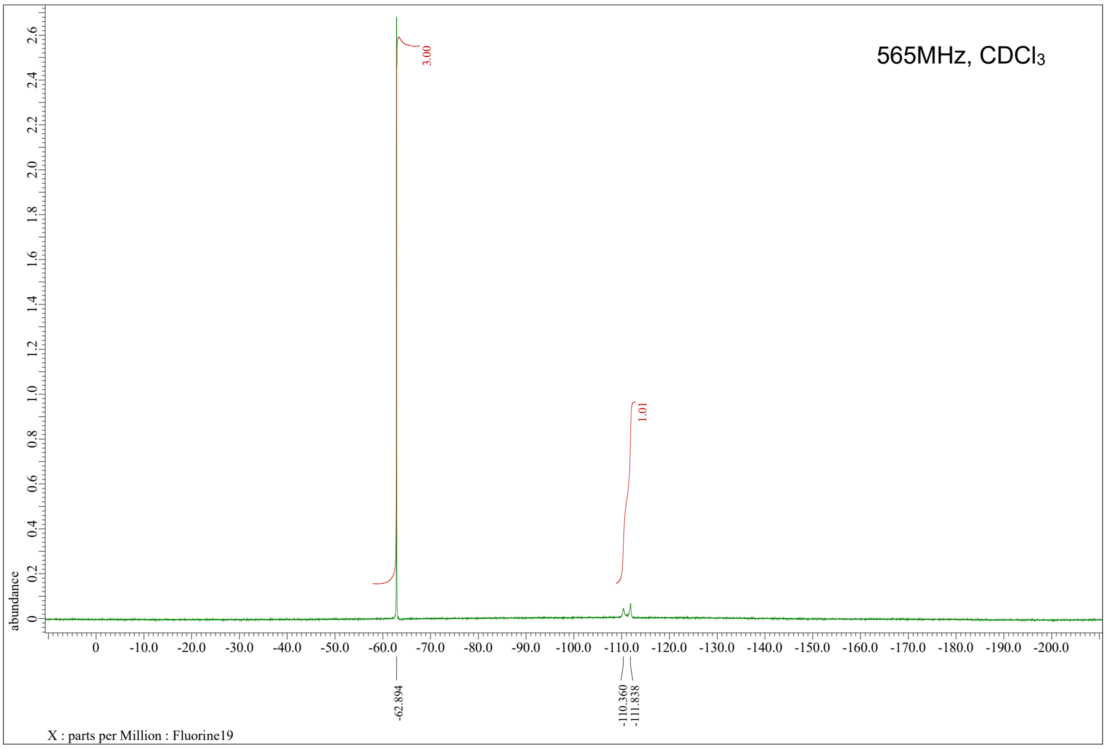

565MHz, CDCl3
3.00
1.01
-62.894
-110.360
-111.838
abundance
X : parts per Million : Fluorine19

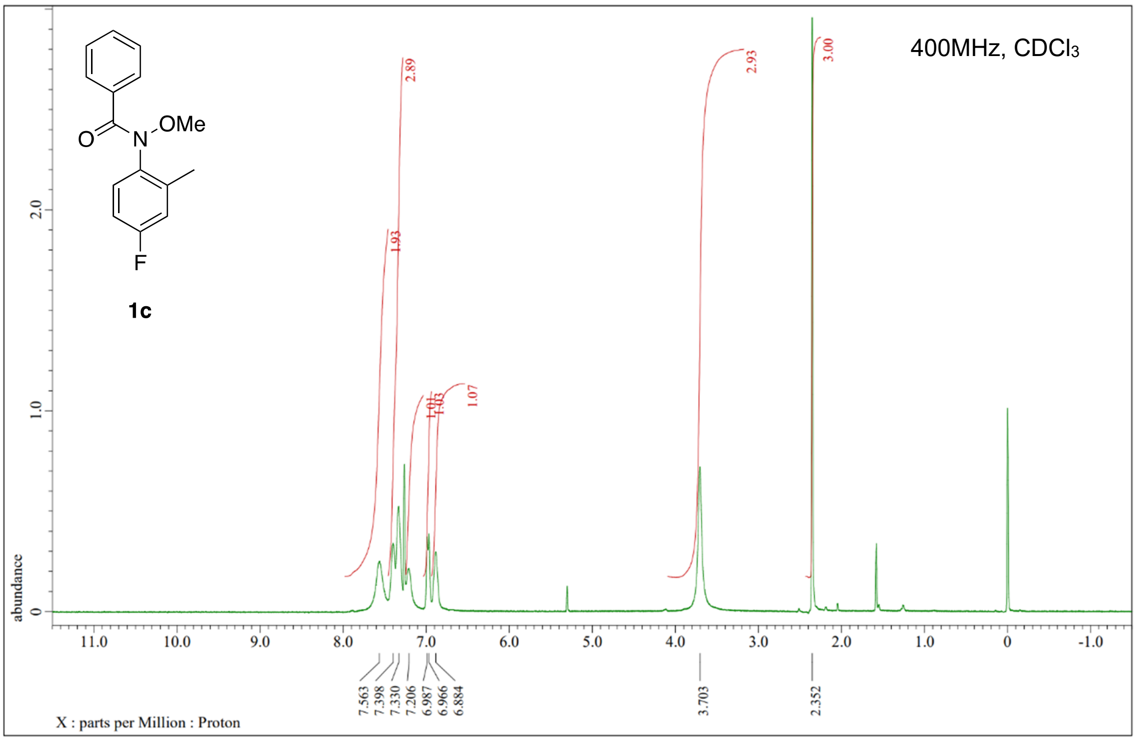

OMe
O
N
F
1c
400MHz, CDCl3
2.89
1.93
1.01
1.03
1.07
2.93
3.00
abundance
X : parts per Million : Proton
7.563
7.398
7.330
7.206
6.987
6.966
6.884
3.703
2.352

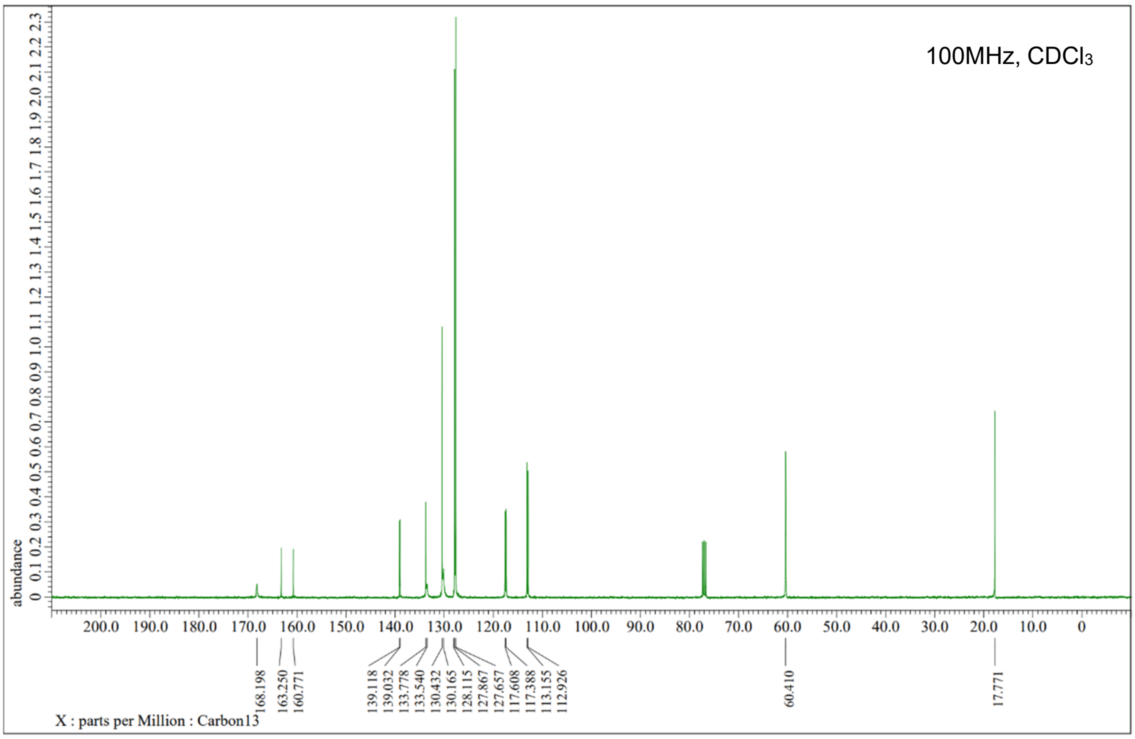

100MHz, CDCl3
abundance
X : parts per Million : Carbon13
168.198
163.250
160.771
139.118
139.032
133.778
133.540
130.432
130.165
128.115
127.867
127.657
117.608
117.388
113.155
112.926
60.410
17.771

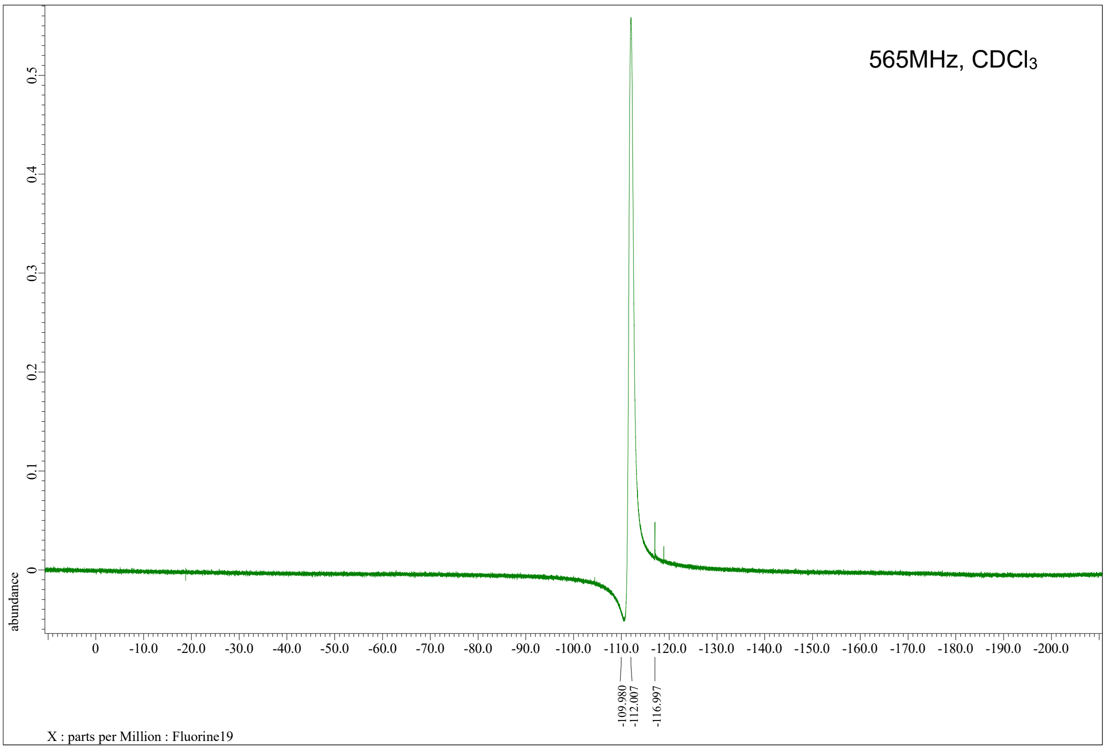

565MHz, CDCl3
abundance
0.5
0.4
0.3
0.2
0.1
0
0
-10.0
-20.0
-30.0
-40.0
-50.0
-60.0
-70.0
-80.0
-90.0
-100.0
-110.0
-120.0
-130.0
-140.0
-150.0
-160.0
-170.0
-180.0
-190.0
-200.0
-109.980
-112.007
-116.997
X : parts per Million : Fluorine19

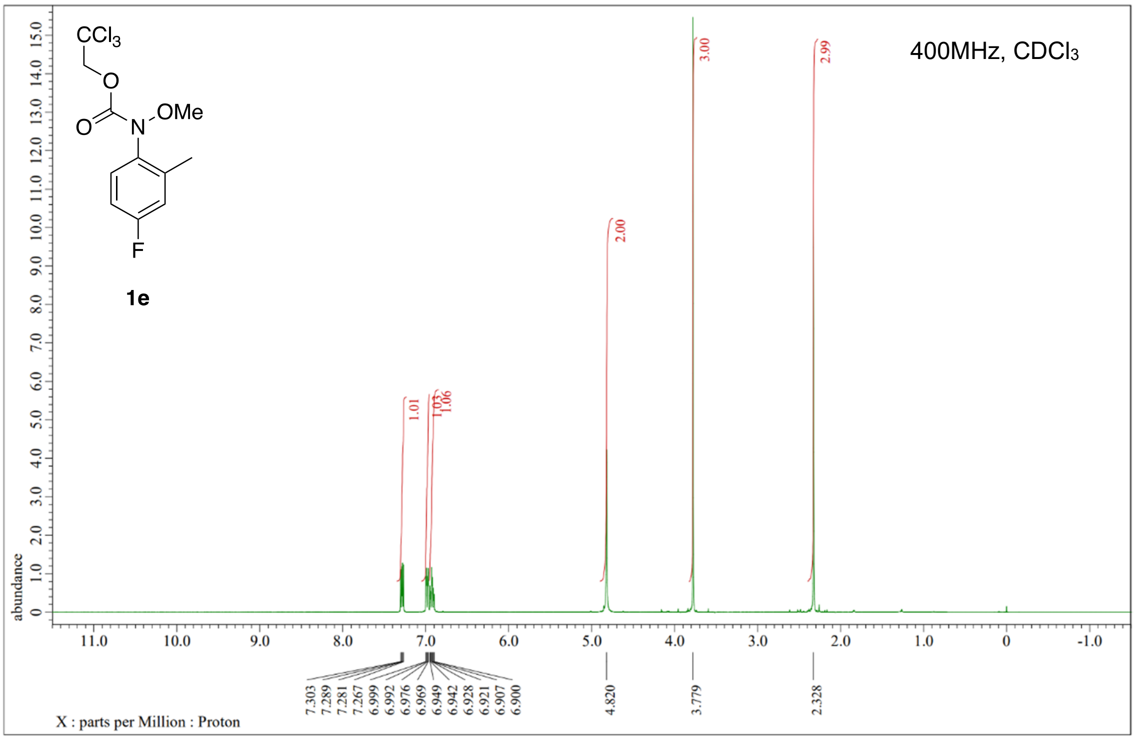
400MHz, CDCl3
CCl3
O
O
N
OMe
F
1e
1.01
1.03
1.06
2.00
3.00
2.99
7.303
7.289
7.281
7.267
6.999
6.992
6.976
6.969
6.949
6.942
6.928
6.921
6.907
6.900
4.820
3.779
2.328
abundance
X : parts per Million : Proton

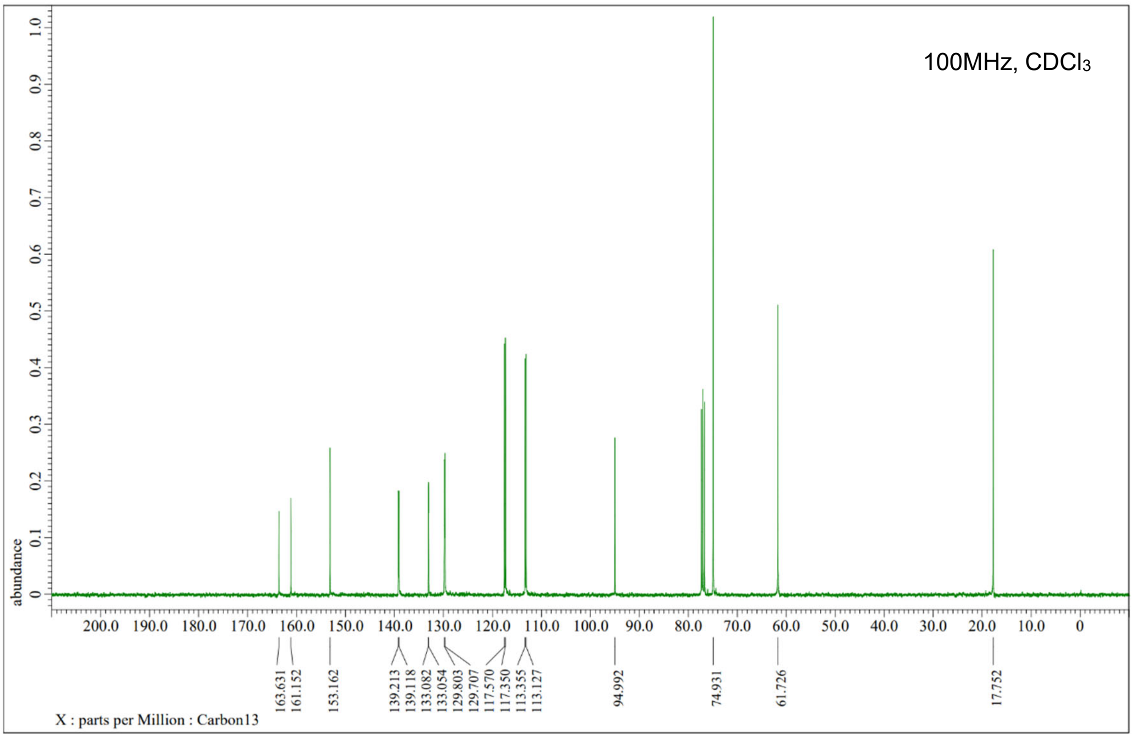
100MHz, CDCl3
163.631
161.152
153.162
139.213
139.118
133.082
133.054
129.803
129.707
117.570
117.350
113.355
113.127
94.992
74.931
61.726
17.752
abundance
X : parts per Million : Carbon13

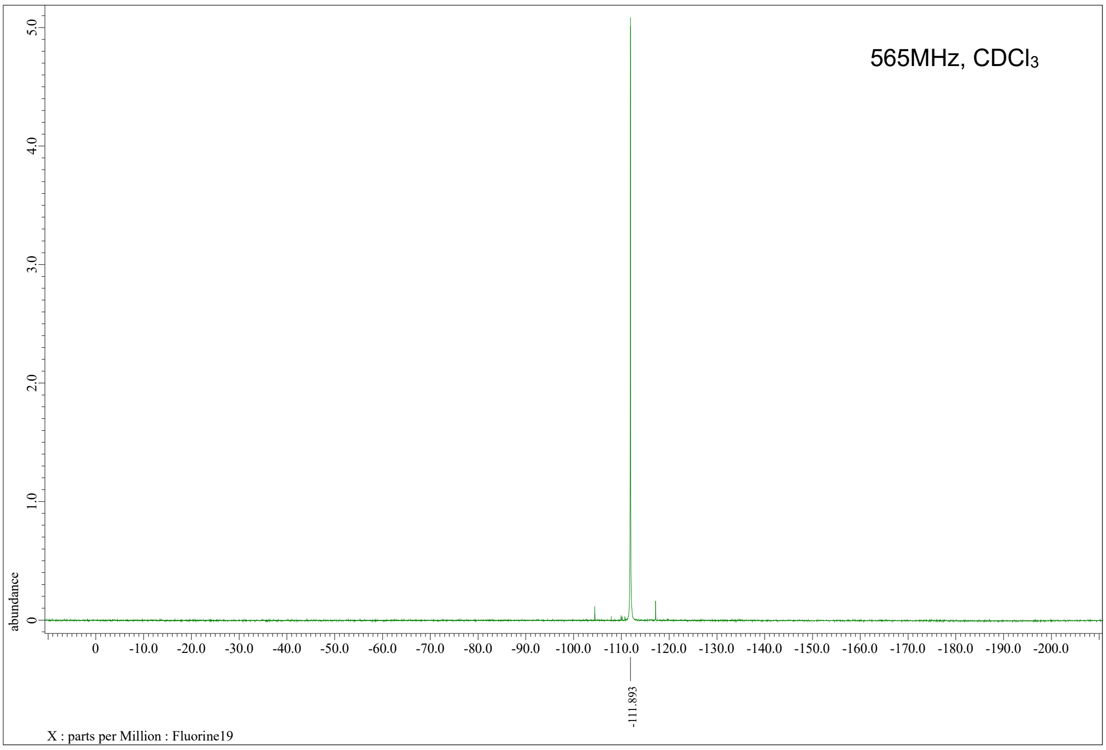
565MHz, CDCl3
abundance
5.0
4.0
3.0
2.0
1.0
0
0
-10.0
-20.0
-30.0
-40.0
-50.0
-60.0
-70.0
-80.0
-90.0
-100.0
-110.0
-120.0
-130.0
-140.0
-150.0
-160.0
-170.0
-180.0
-190.0
-200.0
-111.893
X : parts per Million : Fluorine19

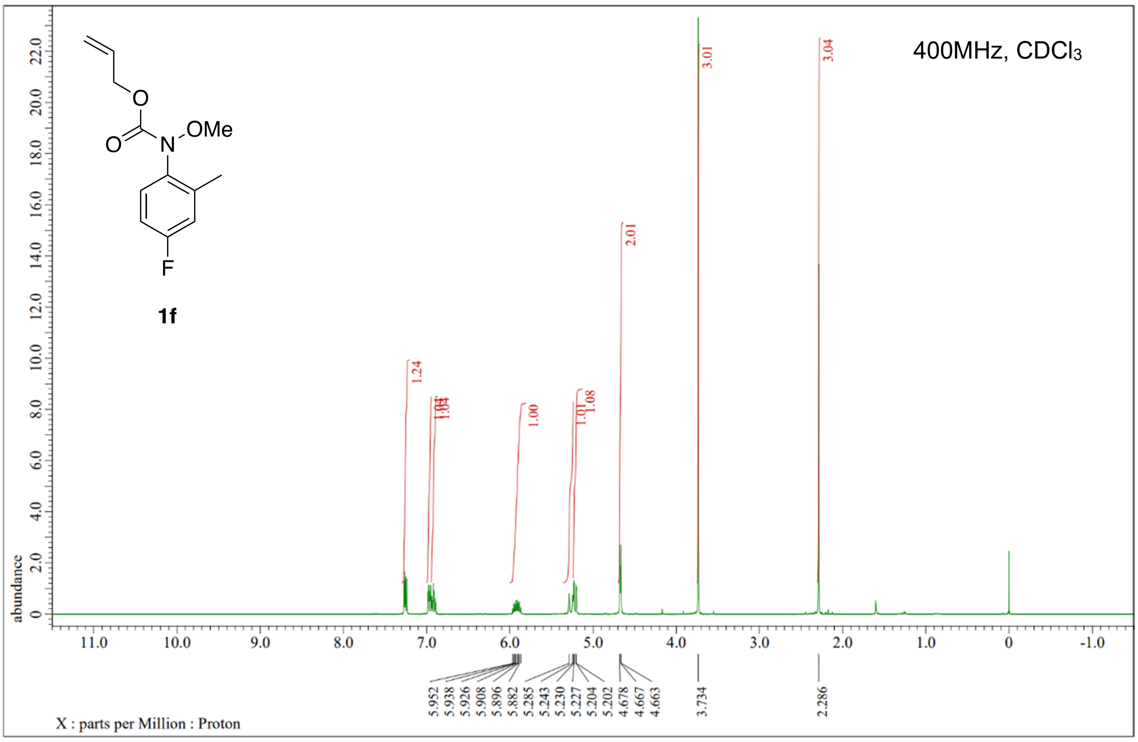

400MHz, CDCl3
O
N
OMe
F
1f
abundance
X : parts per Million : Proton
3.01
3.04
2.01
1.24
1.00
1.08
5.952
5.938
5.926
5.908
5.896
5.882
5.285
5.243
5.230
5.227
5.204
5.202
4.678
4.667
4.663
3.734
2.286

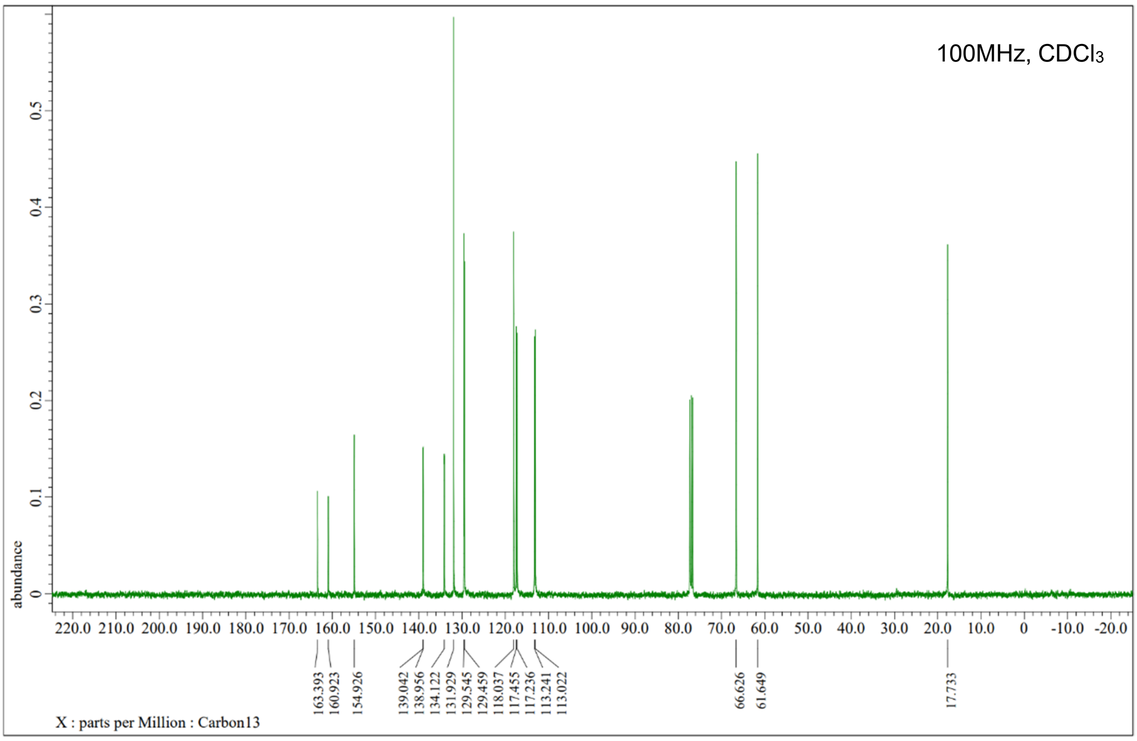

100MHz, CDCl3
abundance
X : parts per Million : Carbon13
163.393
160.923
154.926
139.042
138.956
134.122
131.929
129.545
129.459
118.037
117.455
117.236
113.241
113.022
66.626
61.649
17.733

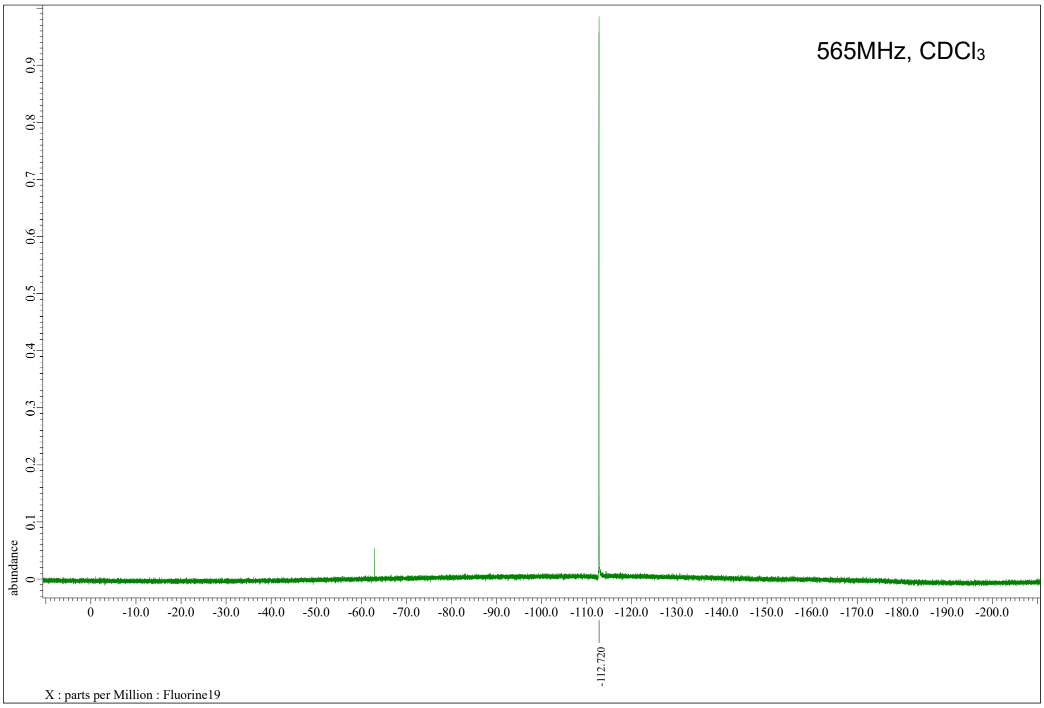
565MHz, CDCl3
abundance
0 0.1 0.2 0.3 0.4 0.5 0.6 0.7 0.8 0.9
0 -10.0 -20.0 -30.0 -40.0 -50.0 -60.0 -70.0 -80.0 -90.0 -100.0 -110.0 -120.0 -130.0 -140.0 -150.0 -160.0 -170.0 -180.0 -190.0 -200.0
-112.720
X : parts per Million : Fluorine19

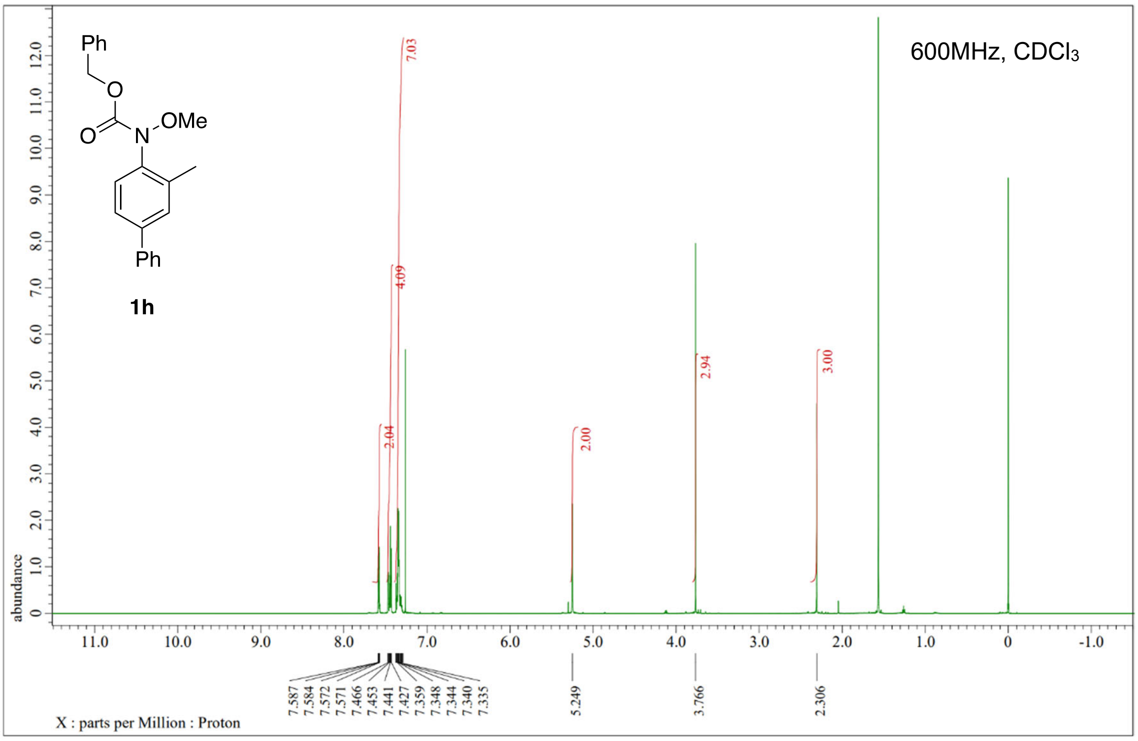
600MHz, CDCl3
Ph
O
O
N
OMe
Ph
1h
7.03
4.09
2.04
2.00
2.94
3.00
abundance
7.587 7.584 7.572 7.571 7.466 7.453 7.441 7.427 7.359 7.348 7.344 7.340 7.335
5.249
3.766
2.306
X : parts per Million : Proton

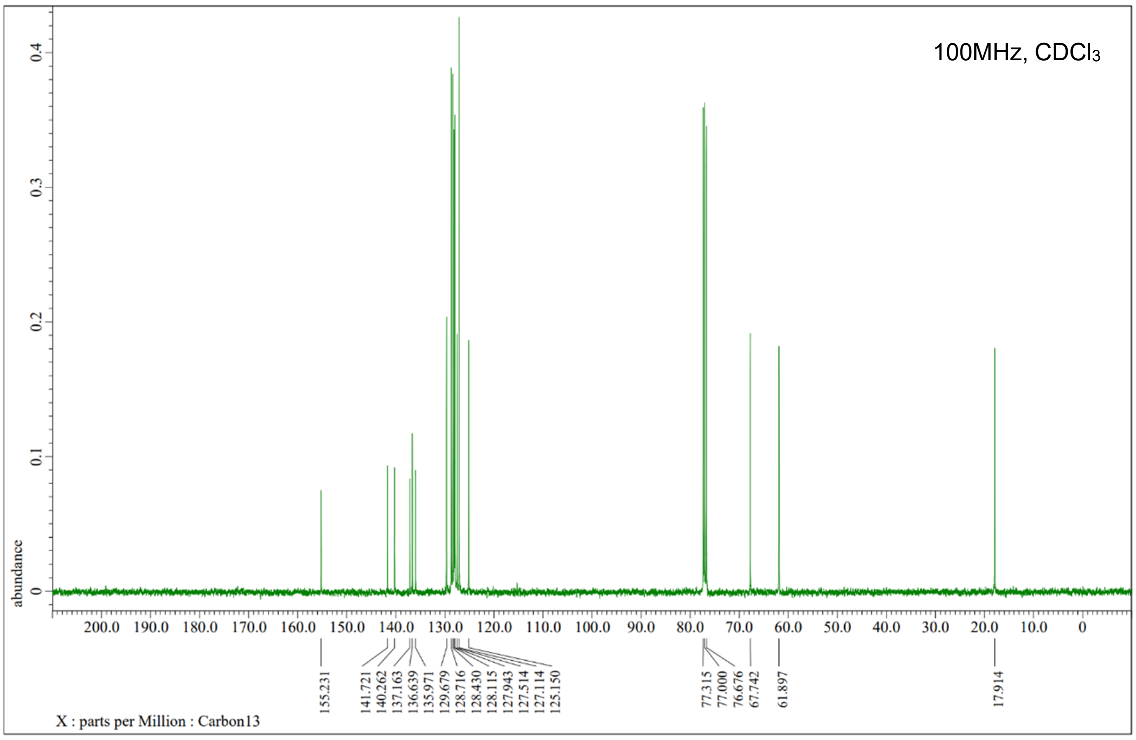
100MHz, CDCl3
abundance
155.231
141.721 140.262 137.163 136.639 135.971 129.679 128.716 128.430 128.115 127.943 127.514 127.114 125.150
77.315 77.000 76.676 67.742
61.897
17.914
X : parts per Million : Carbon13

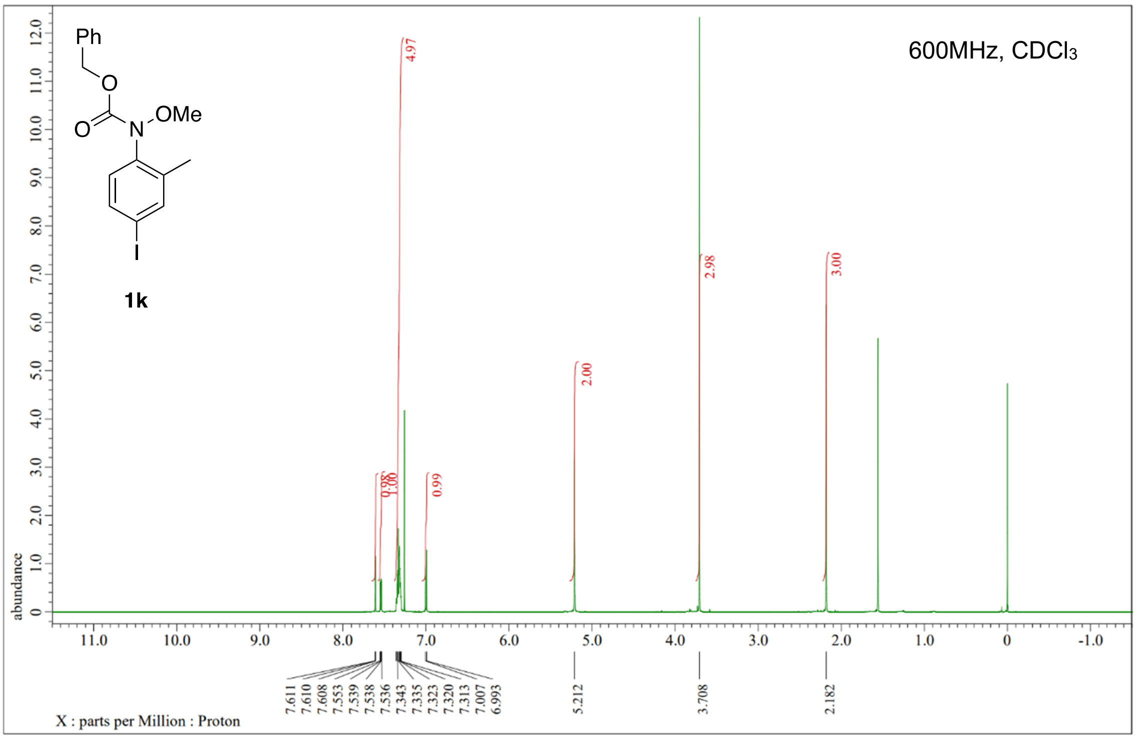
Ph
O
O
N
OMe
I
1k
600MHz, CDCl3
abundance
X : parts per Million : Proton
7.611
7.610
7.608
7.553
7.539
7.538
7.536
7.343
7.335
7.323
7.320
7.313
7.007
6.993
5.212
3.708
2.182
4.97
0.98
1.00
0.99
2.00
2.98
3.00

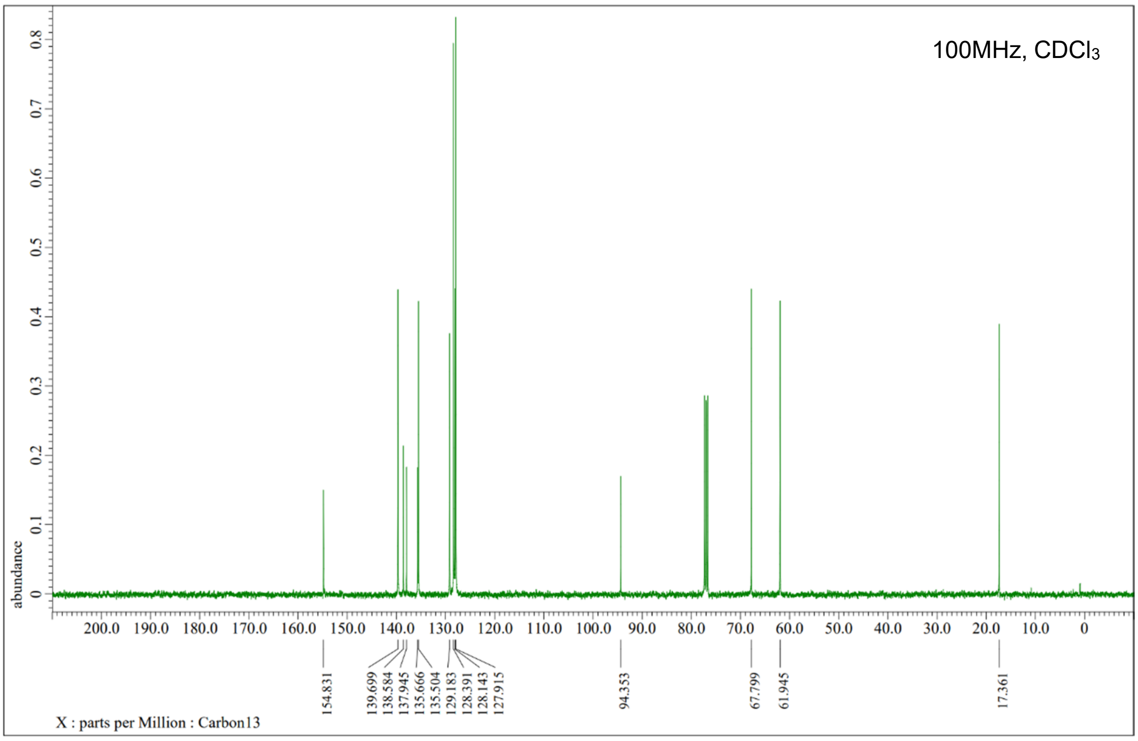
100MHz, CDCl3
abundance
X : parts per Million : Carbon13
154.831
139.699
138.584
137.945
135.666
135.504
129.183
128.391
128.143
127.915
94.353
67.799
61.945
17.361

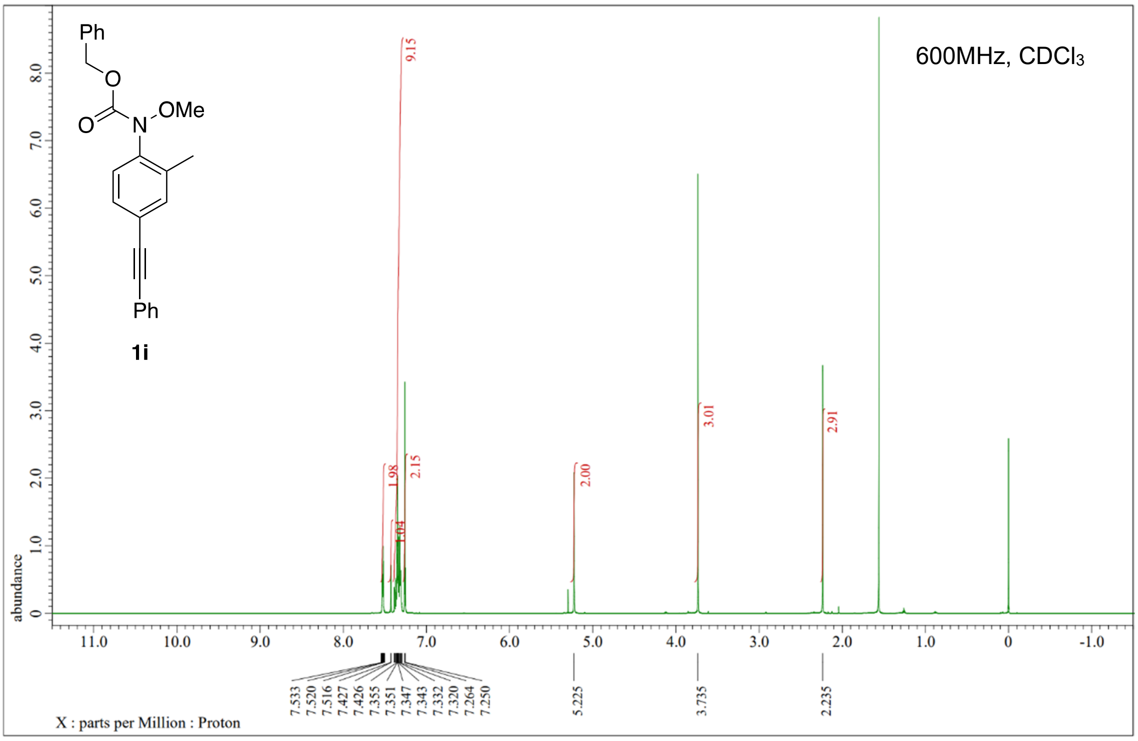

Ph
O
O
N
OMe
Ph
1i
600MHz, CDCl3
abundance
9.15
1.98
1.04
2.15
2.00
3.01
2.91
7.533
7.520
7.516
7.427
7.426
7.355
7.351
7.347
7.343
7.332
7.320
7.264
7.250
5.225
3.735
2.235
X : parts per Million : Proton

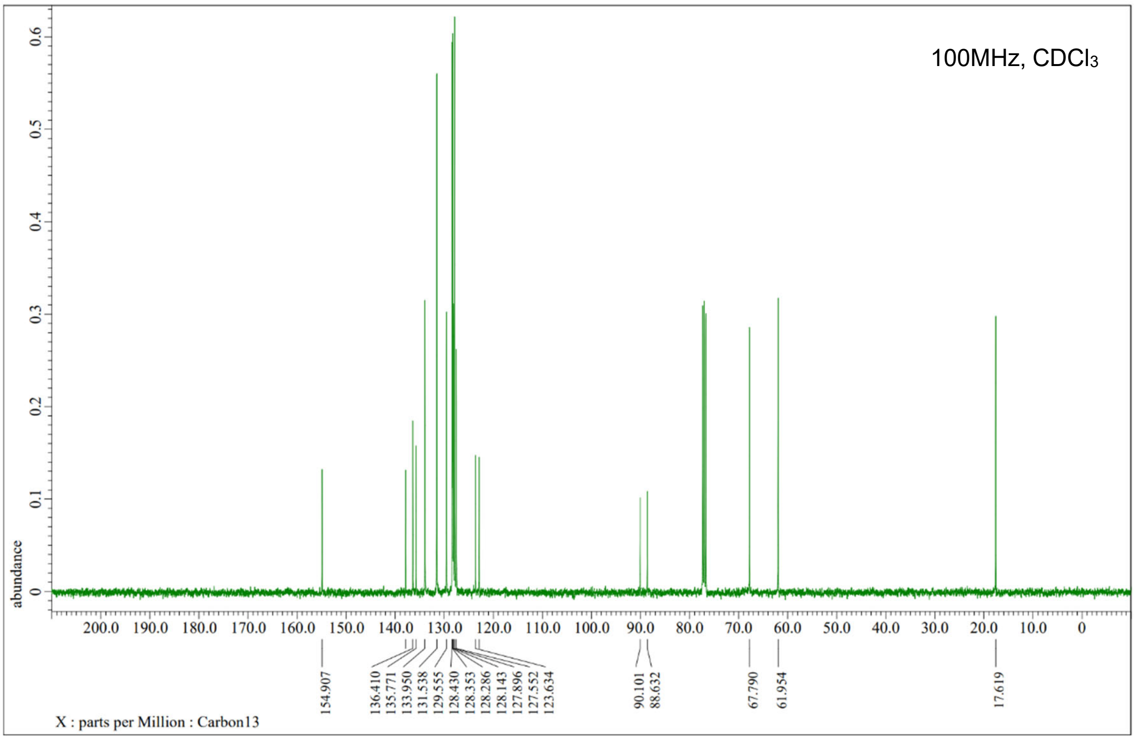

100MHz, CDCl3
abundance
154.907
136.410
135.771
133.950
131.538
129.555
128.430
128.353
128.286
128.143
127.896
127.552
123.634
90.101
88.632
67.790
61.954
17.619
X : parts per Million : Carbon13

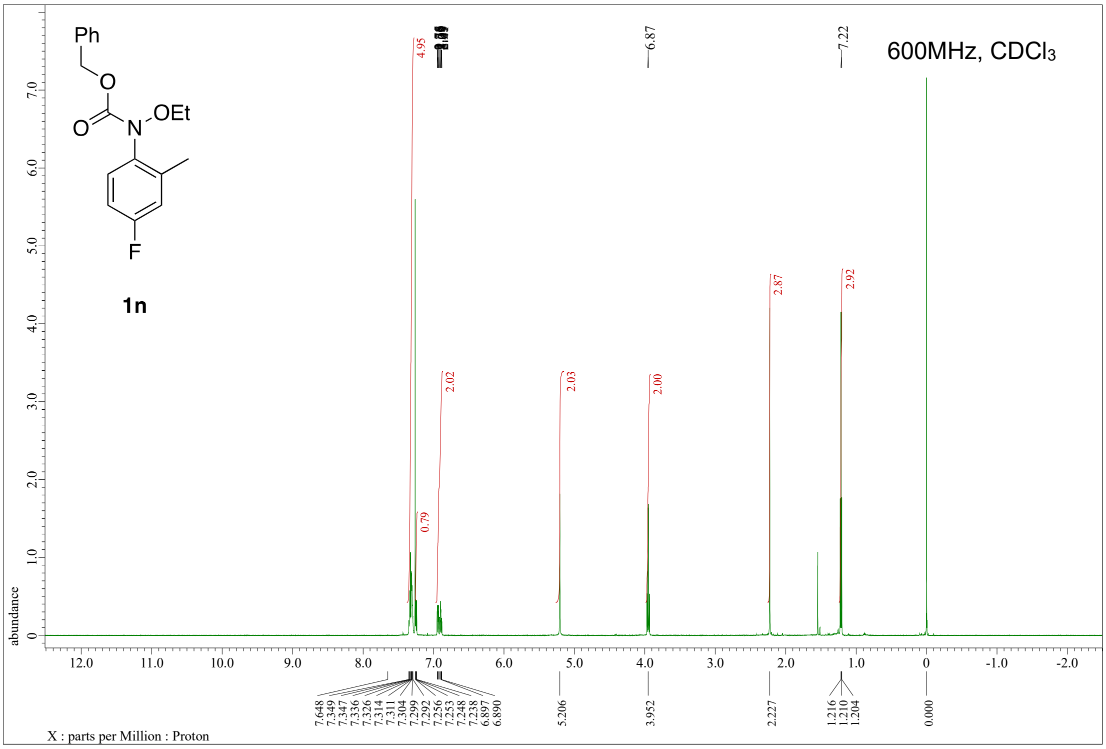
600MHz, CDCl3
Ph
O
O
N
OEt
F
1n
X : parts per Million : Proton
abundance

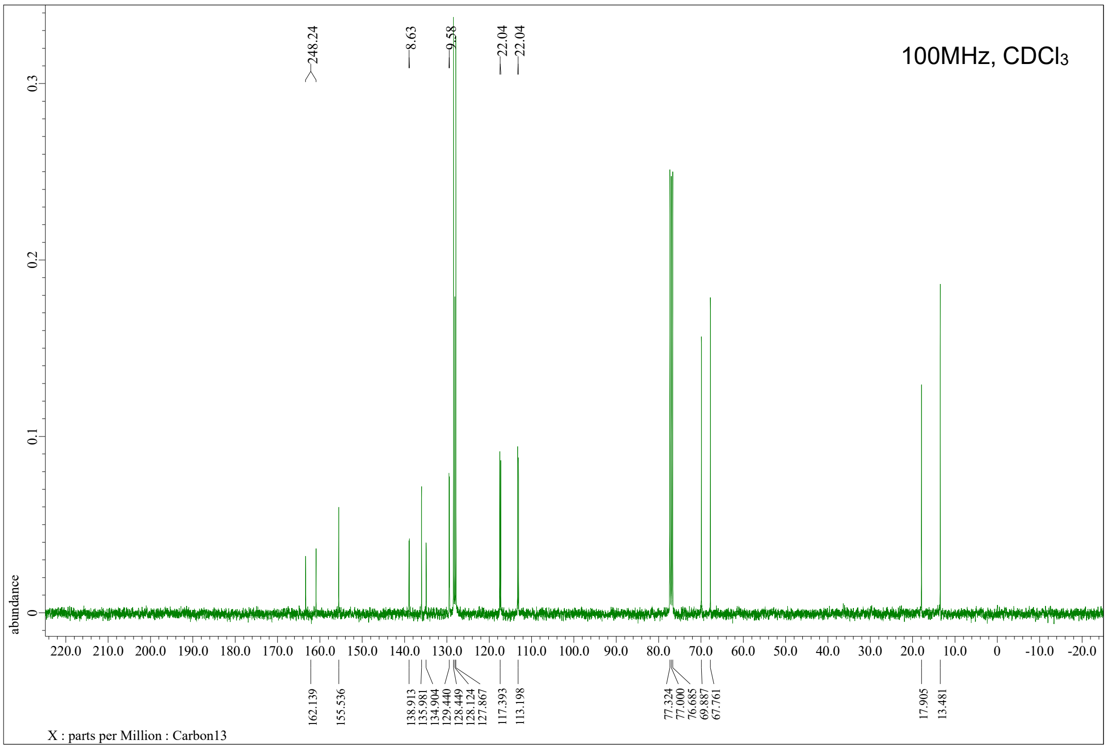
100MHz, CDCl3
X : parts per Million : Carbon13
abundance

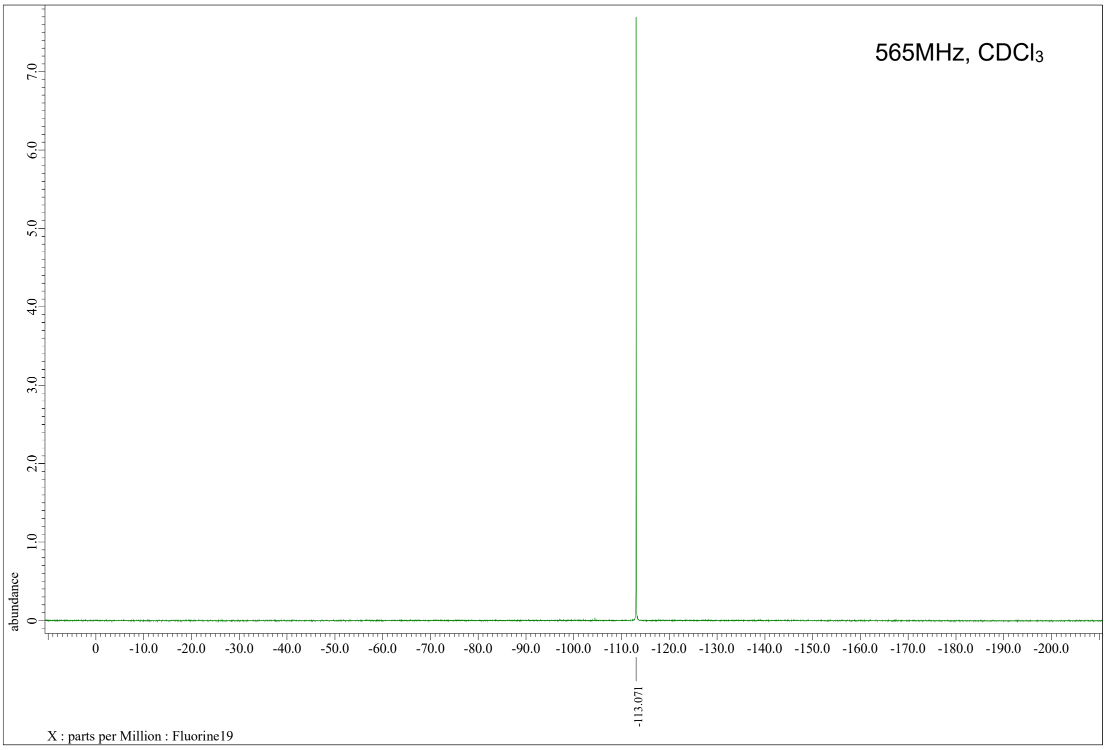
565MHz, CDCl3
abundance
7.0
6.0
5.0
4.0
3.0
2.0
1.0
0
0
-10.0
-20.0
-30.0
-40.0
-50.0
-60.0
-70.0
-80.0
-90.0
-100.0
-110.0
-120.0
-130.0
-140.0
-150.0
-160.0
-170.0
-180.0
-190.0
-200.0
-113.071
X : parts per Million : Fluorine19

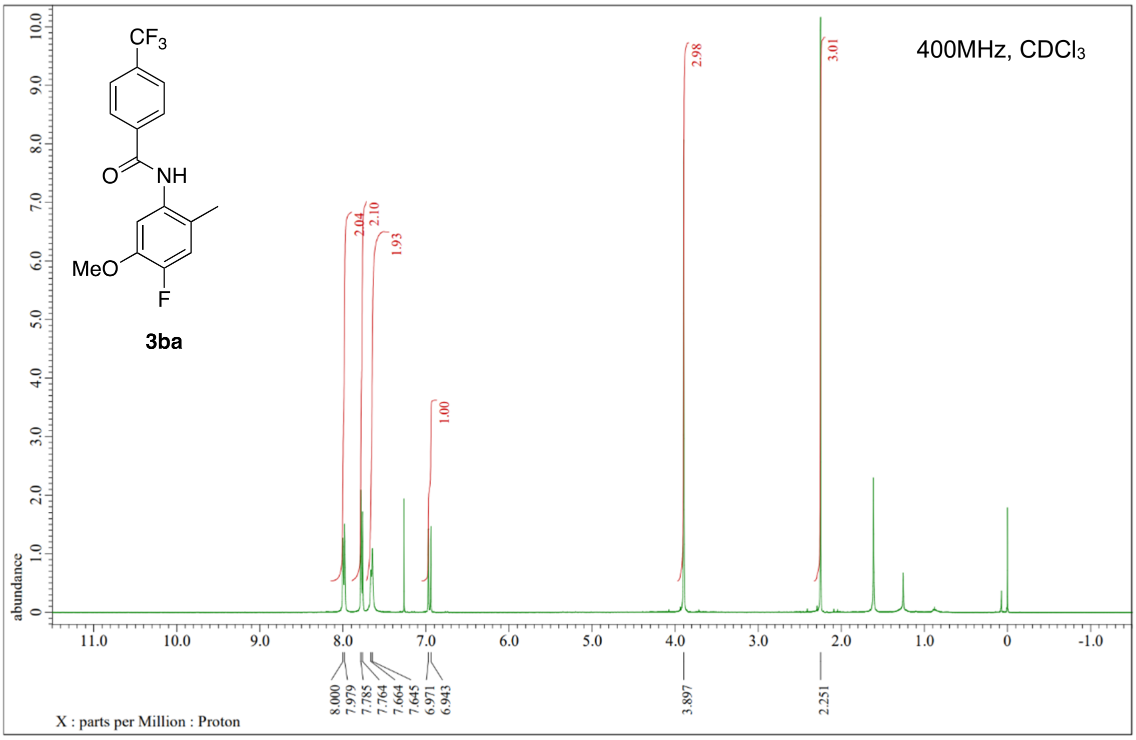
CF3
O
NH
MeO
F
3ba
400MHz, CDCl3
abundance
X : parts per Million : Proton
2.04
2.10
1.93
1.00
2.98
3.01
8.000
7.979
7.785
7.764
7.664
7.645
6.971
6.943
3.897
2.251

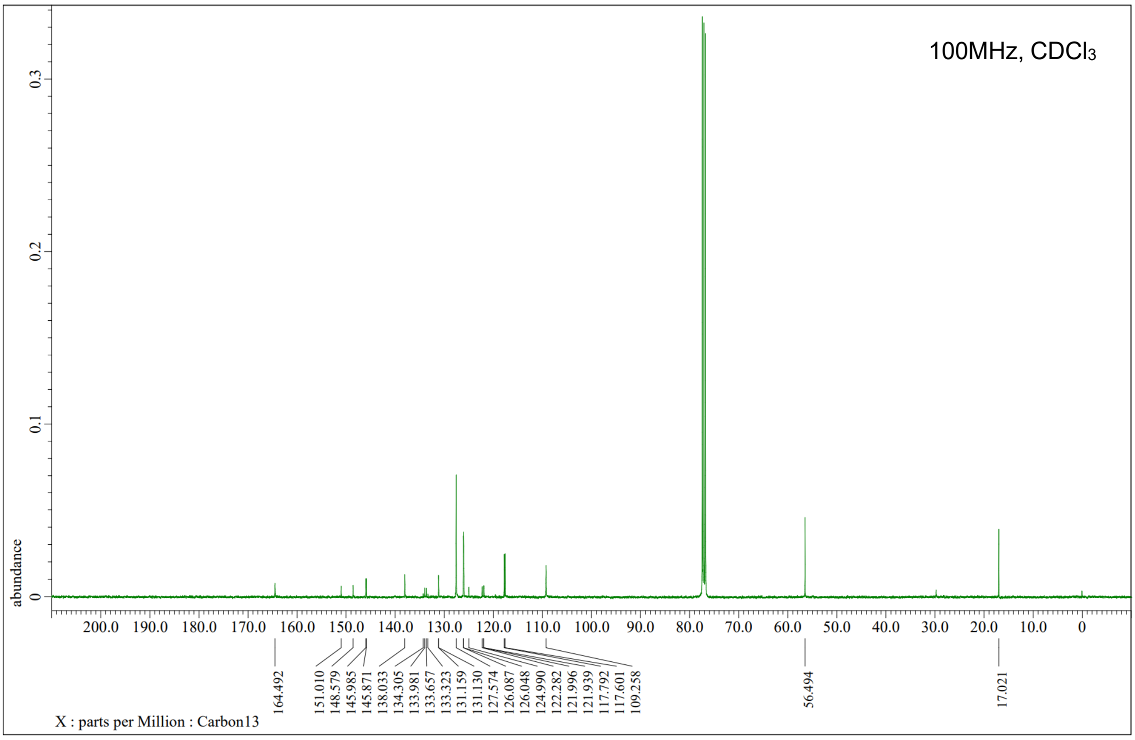
100MHz, CDCl3
abundance
X : parts per Million : Carbon13
164.492
151.010
148.579
145.985
145.871
138.033
134.305
133.981
133.657
133.323
131.159
131.130
127.574
126.087
126.048
124.990
122.282
121.996
121.939
117.792
117.601
109.258
56.494
17.021

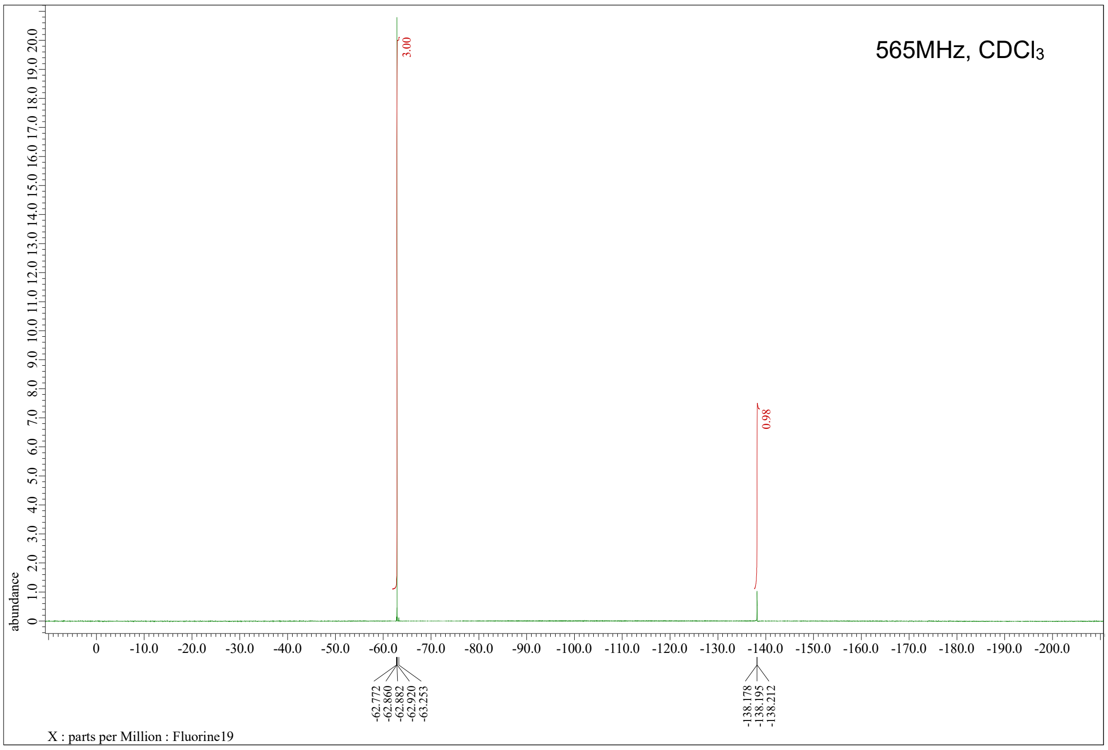

565MHz, CDCl3
3.00
0.98
abundance
0 -10.0 -20.0 -30.0 -40.0 -50.0 -60.0 -70.0 -80.0 -90.0 -100.0 -110.0 -120.0 -130.0 -140.0 -150.0 -160.0 -170.0 -180.0 -190.0 -200.0
-62.772
-62.860
-62.882
-62.920
-63.253
-138.178
-138.195
-138.212
X : parts per Million : Fluorine19

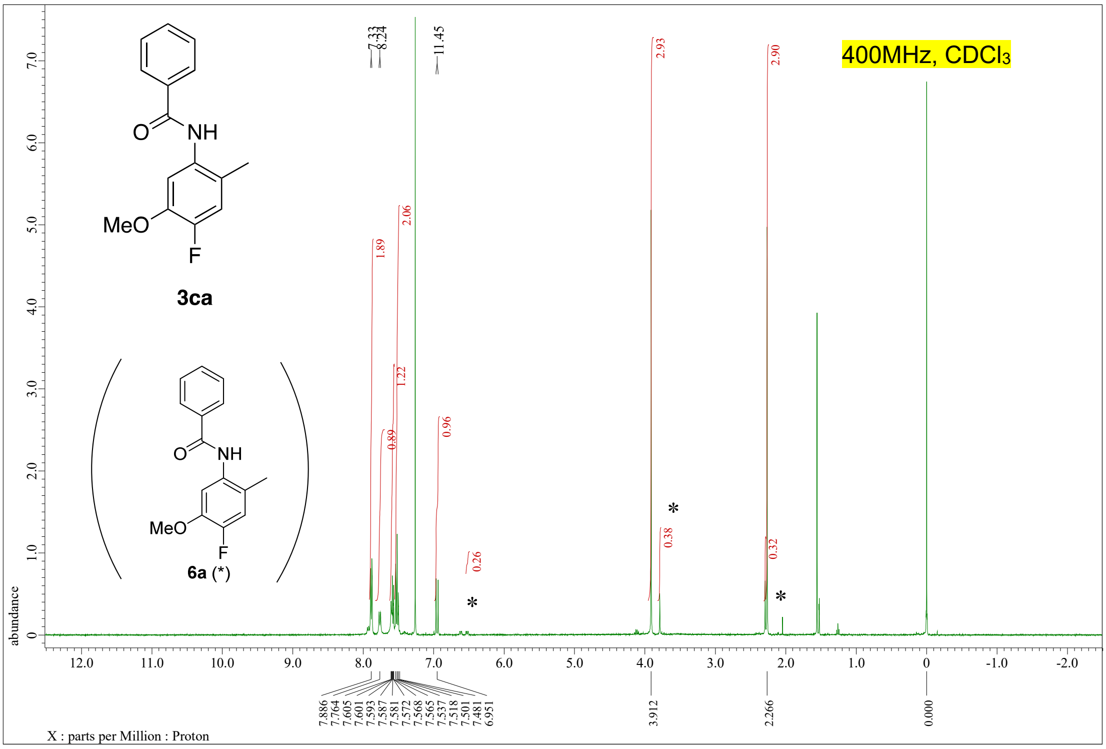
400MHz, CDCl3
3ca
6a (*)
X : parts per Million : Proton
abundance
7.886
7.764
7.605
7.601
7.593
7.587
7.581
7.572
7.568
7.565
7.537
7.518
7.501
7.481
6.951
3.912
2.266
0.000
1.89
0.89
1.22
2.06
0.96
0.26
2.93
0.38
2.90
0.32

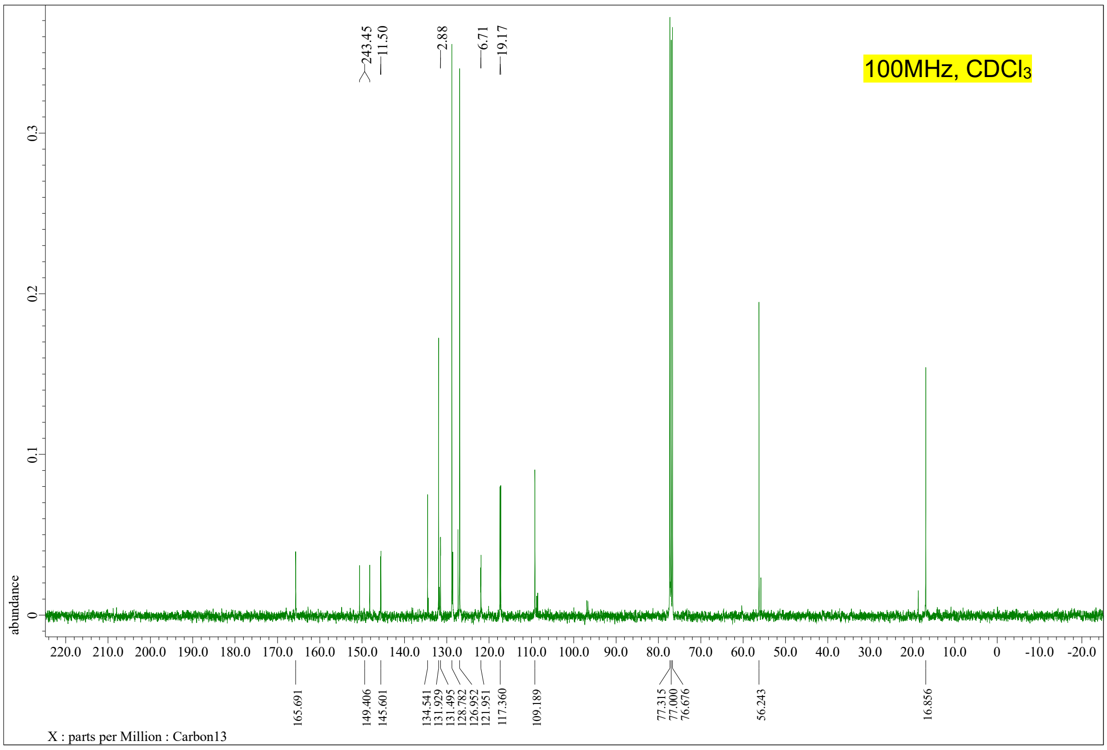
100MHz, CDCl3
X : parts per Million : Carbon13
abundance
165.691
149.406
145.601
134.541
131.929
131.495
128.782
126.952
121.951
117.360
109.189
77.315
77.000
76.676
56.243
16.856

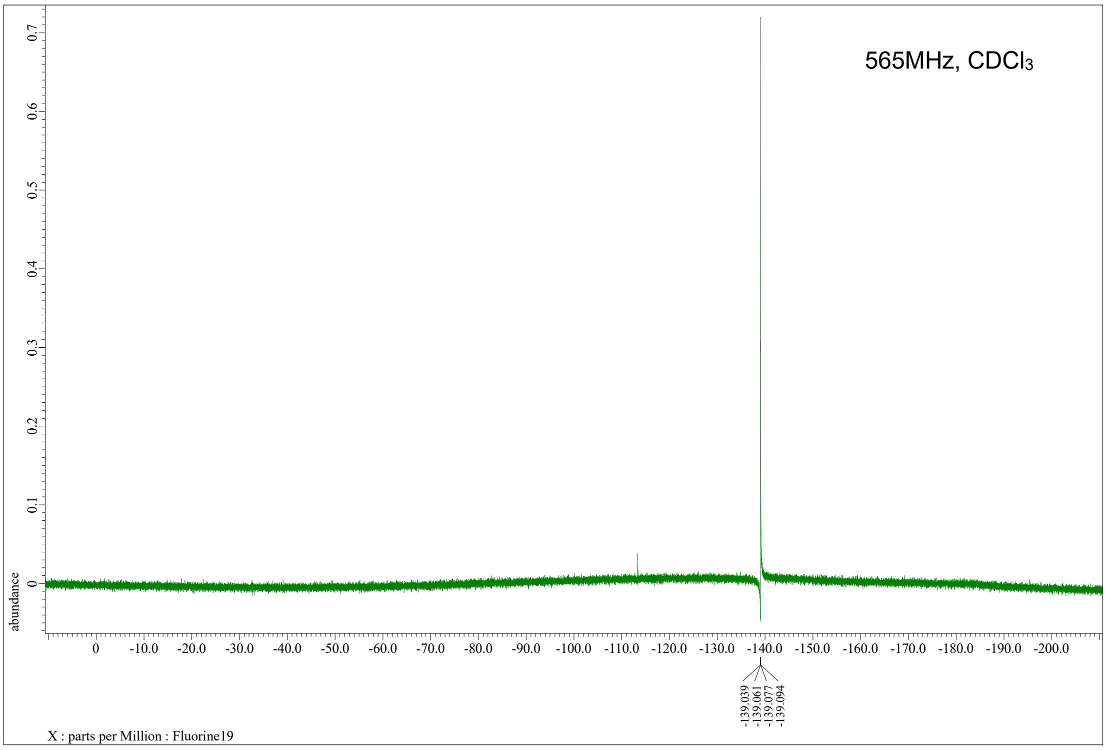
565MHz, CDCl3
abundance
X : parts per Million : Fluorine19
-139.039
-139.061
-139.077
-139.094

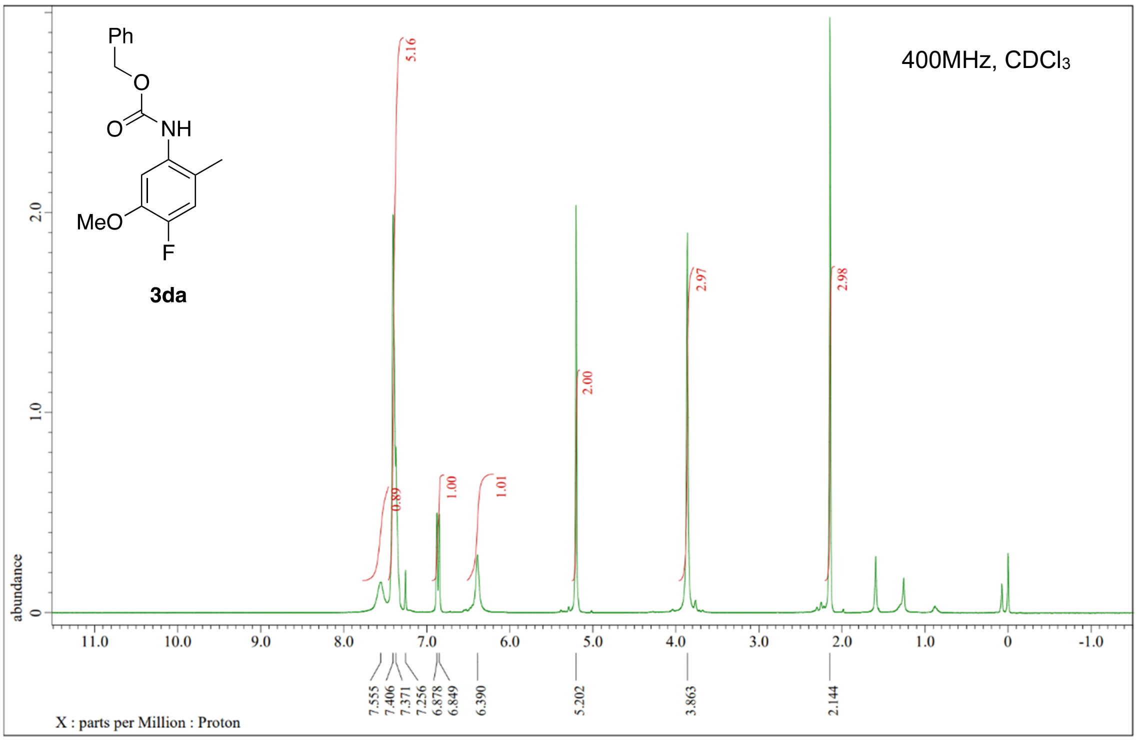

Ph
O
O
NH
MeO
F
3da
400MHz, CDCl3
5.16
0.89
1.00
1.01
2.00
2.97
2.98
7.555
7.406
7.371
7.256
6.878
6.849
6.390
5.202
3.863
2.144
X : parts per Million : Proton
abundance

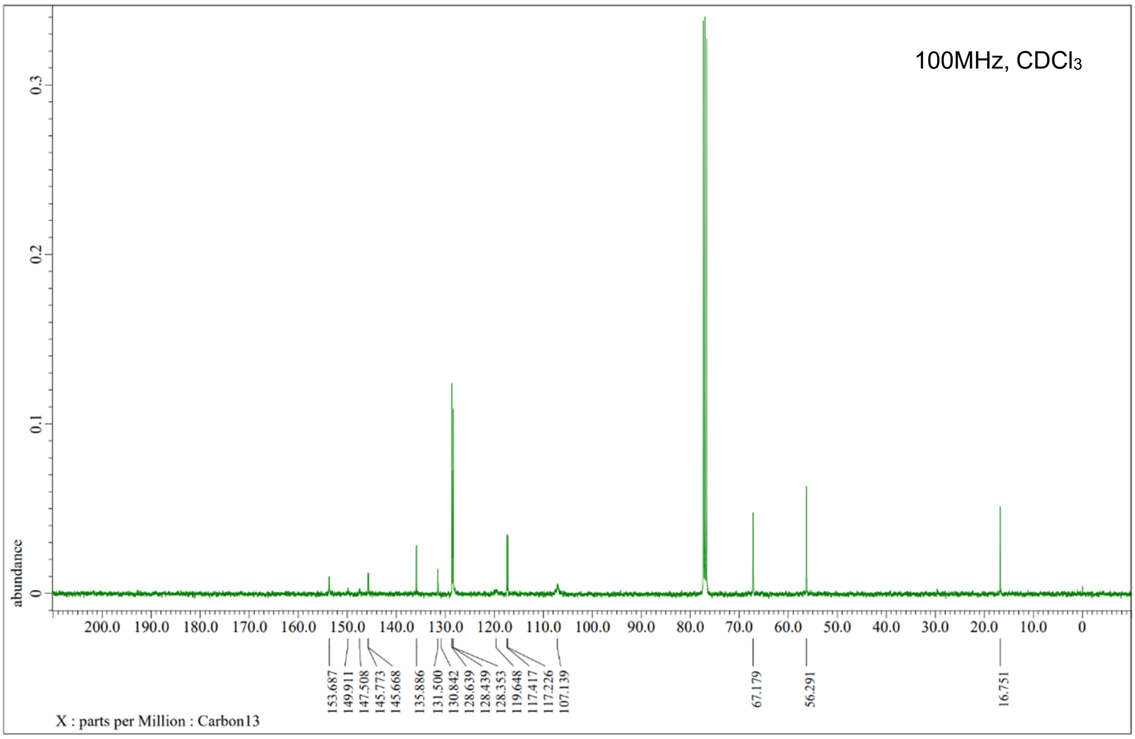

100MHz, CDCl3
153.687
149.911
147.508
145.773
145.668
135.886
131.500
130.842
128.639
128.439
128.353
119.648
117.417
117.226
107.139
67.179
56.291
16.751
X : parts per Million : Carbon13
abundance

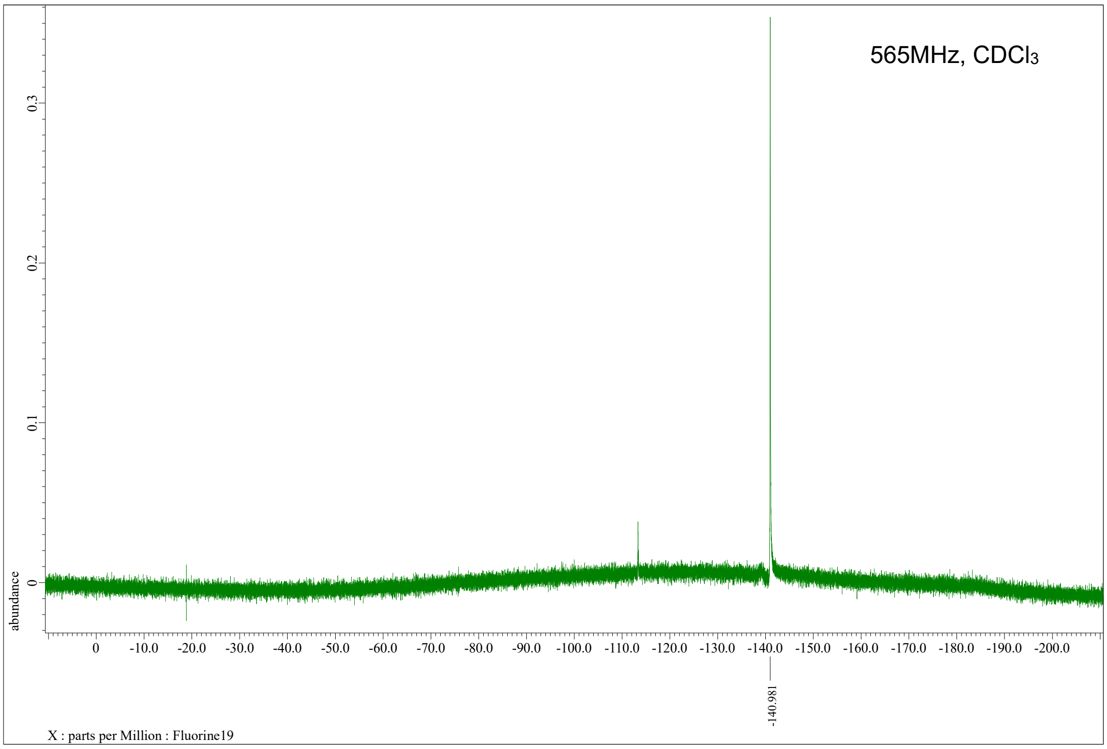
565MHz, CDCl3
abundance
0.3
0.2
0.1
0
0
-10.0
-20.0
-30.0
-40.0
-50.0
-60.0
-70.0
-80.0
-90.0
-100.0
-110.0
-120.0
-130.0
-140.0
-150.0
-160.0
-170.0
-180.0
-190.0
-200.0
-140.981
X : parts per Million : Fluorine19

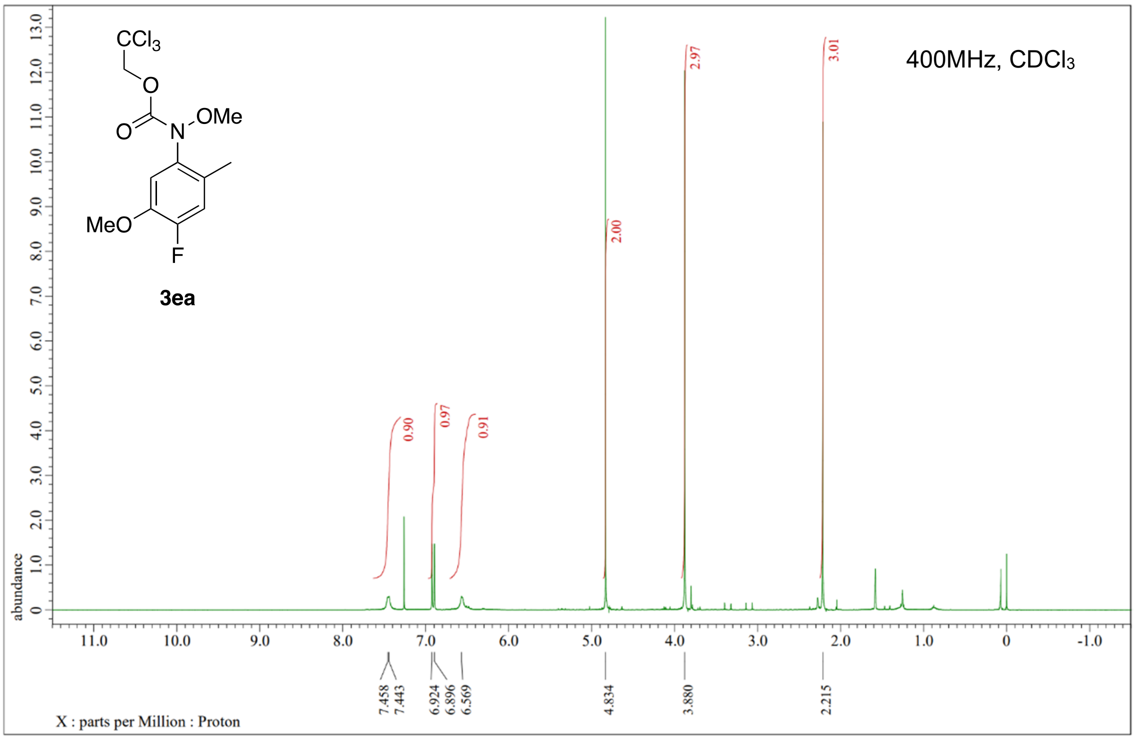

CCl3
O
O
N
OMe
MeO
F
3ea
400MHz, CDCl3
0.90
0.97
0.91
2.00
2.97
3.01
7.458
7.443
6.924
6.896
6.569
4.834
3.880
2.215
abundance
X : parts per Million : Proton

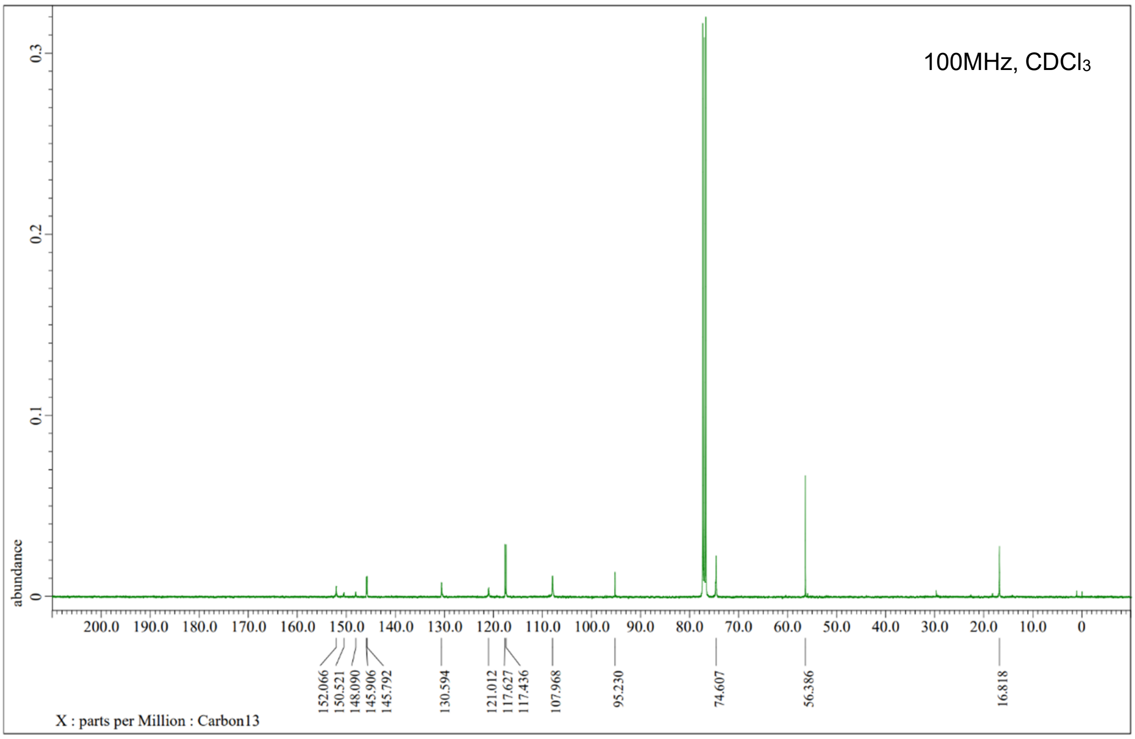

100MHz, CDCl3
152.066
150.521
148.090
145.906
145.792
130.594
121.012
117.627
117.436
107.968
95.230
74.607
56.386
16.818
abundance
X : parts per Million : Carbon13

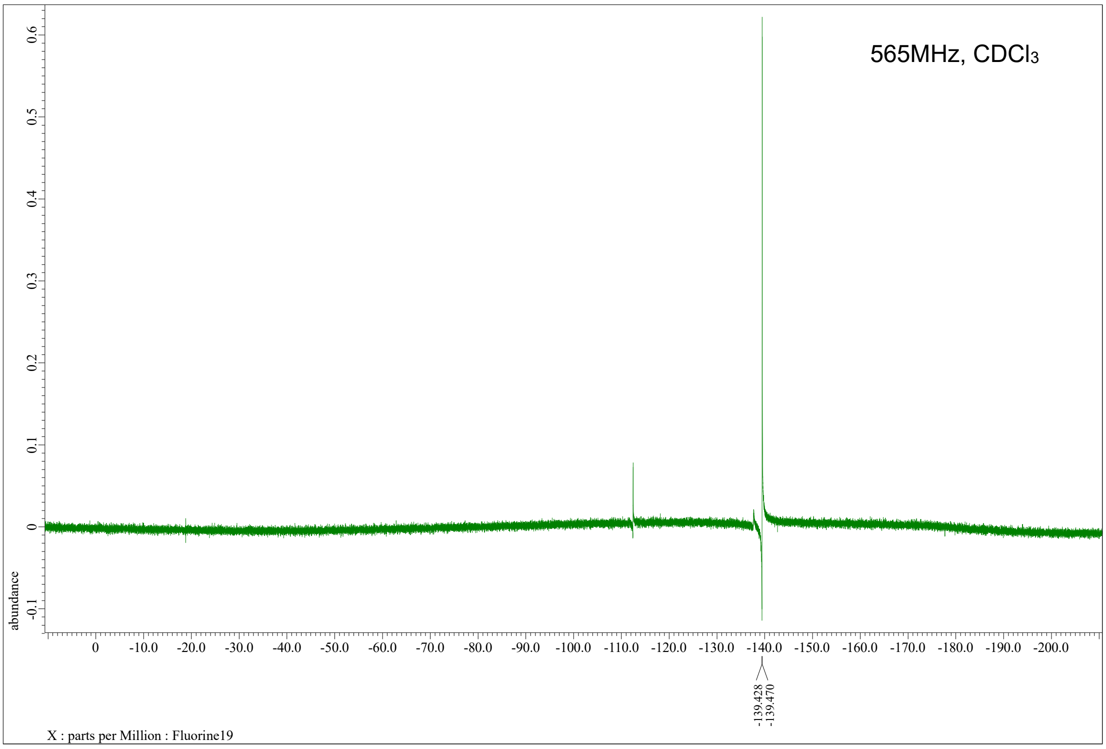
565MHz, CDCl3
abundance
X : parts per Million : Fluorine19
-139.428
-139.470

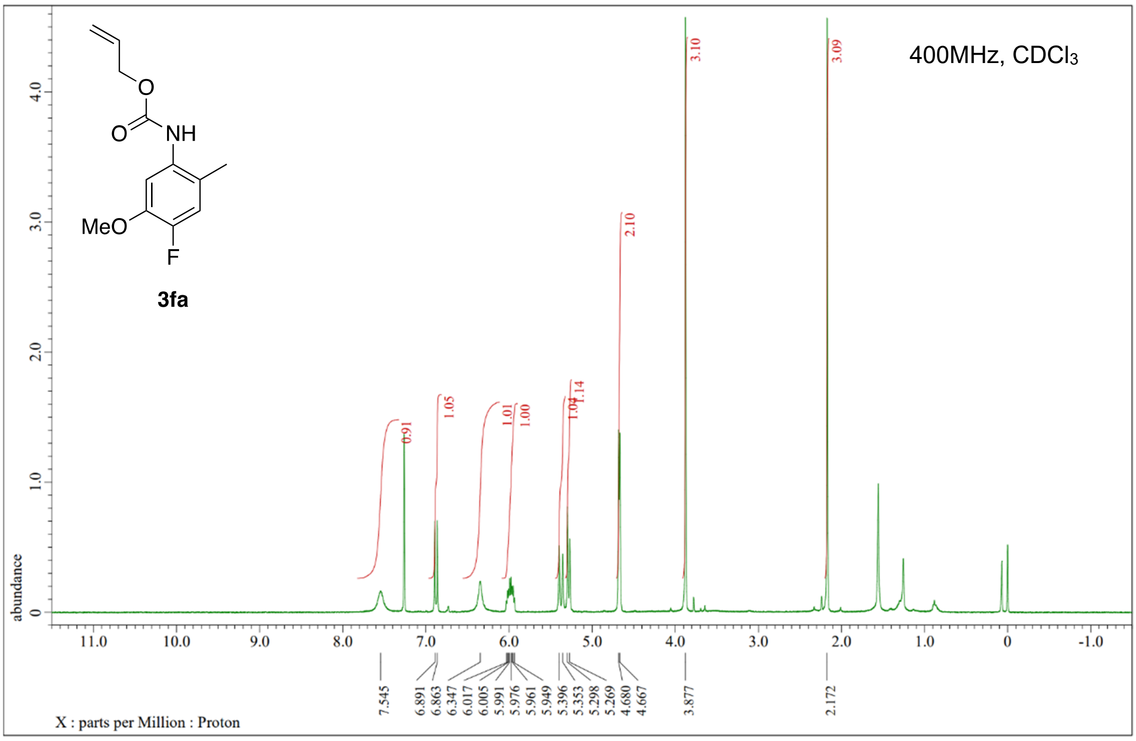

400MHz, CDCl3
MeO
F
NH
O
O
3fa
abundance
X : parts per Million : Proton
7.545
6.891
6.863
6.347
6.017
6.005
5.991
5.976
5.961
5.949
5.396
5.353
5.298
5.269
4.680
4.667
3.877
2.172

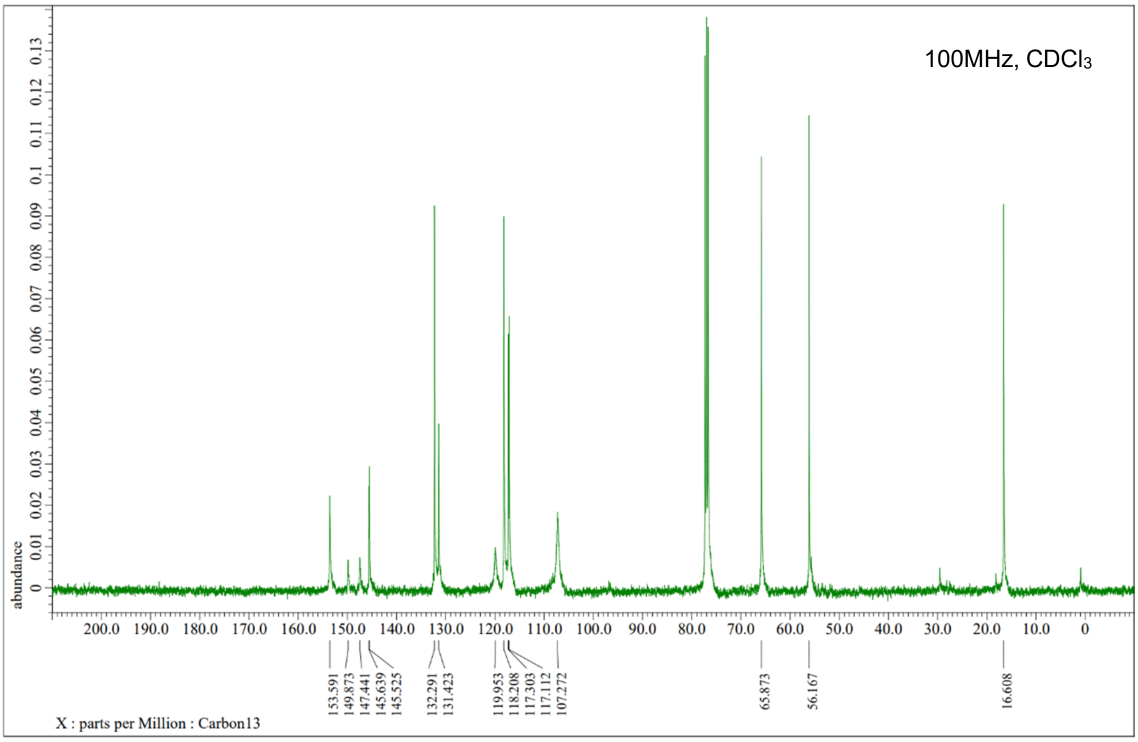

100MHz, CDCl3
abundance
X : parts per Million : Carbon13
153.591
149.873
147.441
145.639
145.525
132.291
131.423
119.953
118.208
117.303
117.112
107.272
65.873
56.167
16.608

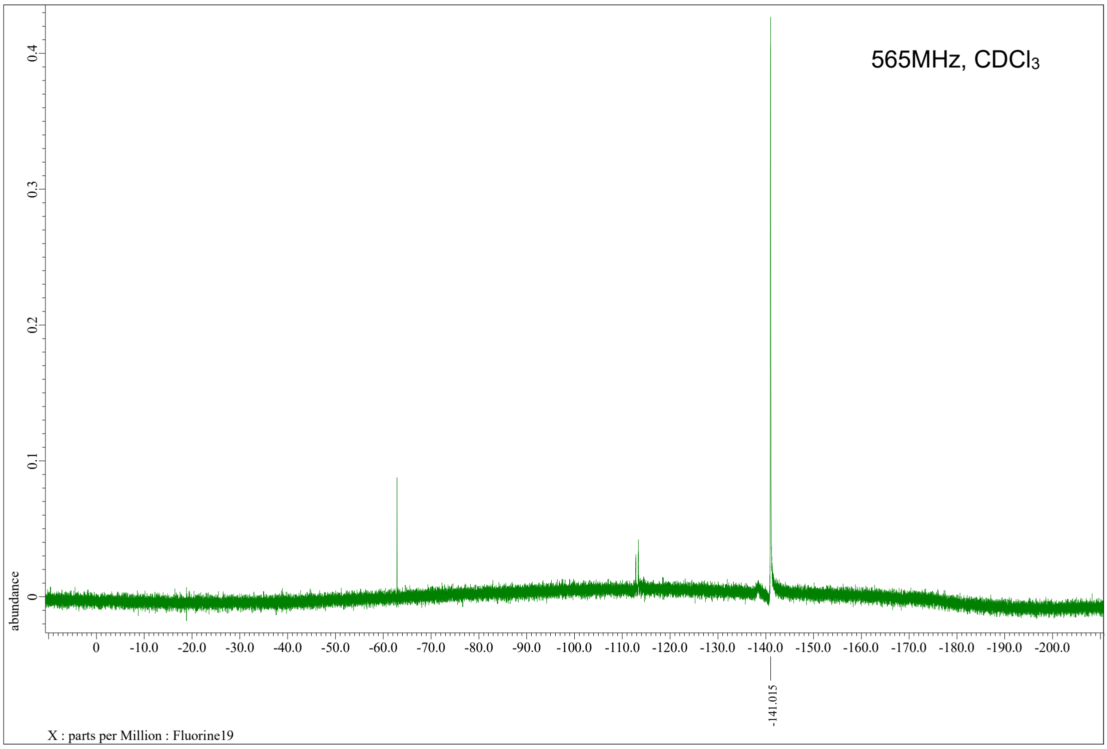

565MHz, CDCl3
abundance
0.4
0.3
0.2
0.1
0
0
-10.0
-20.0
-30.0
-40.0
-50.0
-60.0
-70.0
-80.0
-90.0
-100.0
-110.0
-120.0
-130.0
-140.0
-150.0
-160.0
-170.0
-180.0
-190.0
-200.0
-141.015
X : parts per Million : Fluorine19

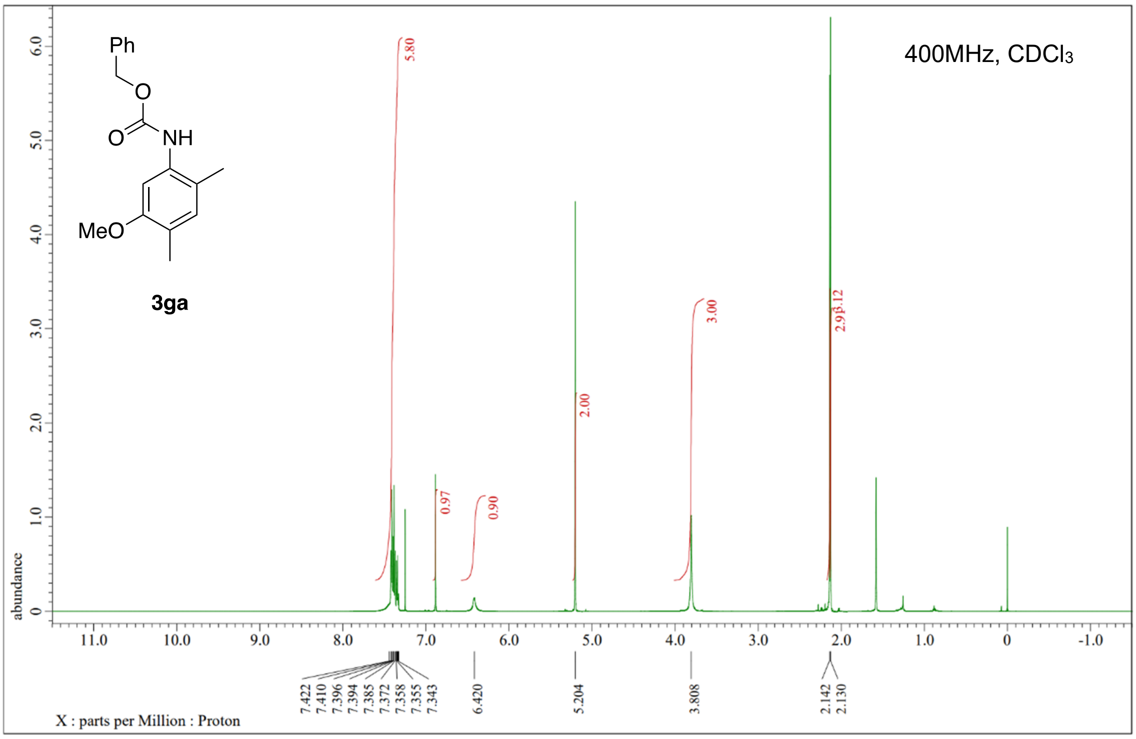
Ph
O
O
NH
MeO
3ga
400MHz, CDCl3
5.80
0.97
0.90
2.00
3.00
2.9β.12
abundance
X : parts per Million : Proton
7.422
7.410
7.396
7.394
7.385
7.372
7.358
7.355
7.343
6.420
5.204
3.808
2.142
2.130

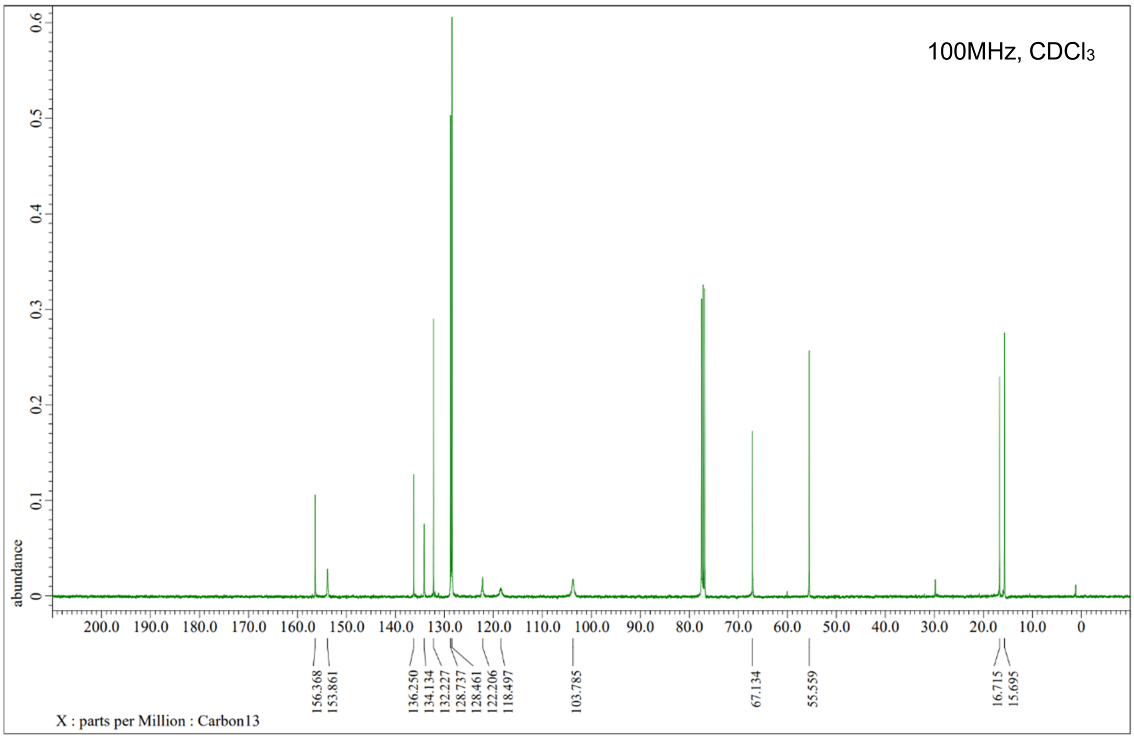
100MHz, CDCl3
abundance
X : parts per Million : Carbon13
156.368
153.861
136.250
134.134
132.227
128.737
128.461
122.206
118.497
103.785
67.134
55.559
16.715
15.695

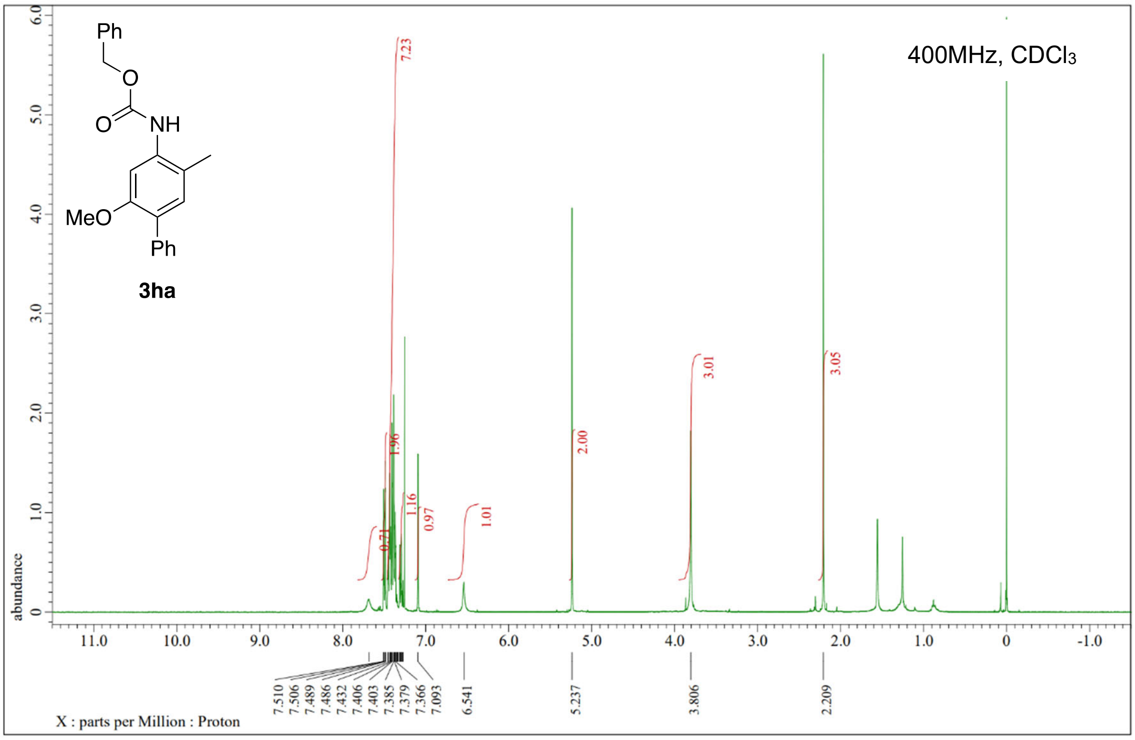
400MHz, CDCl3
3ha
MeO
Ph
NH
X : parts per Million : Proton
abundance

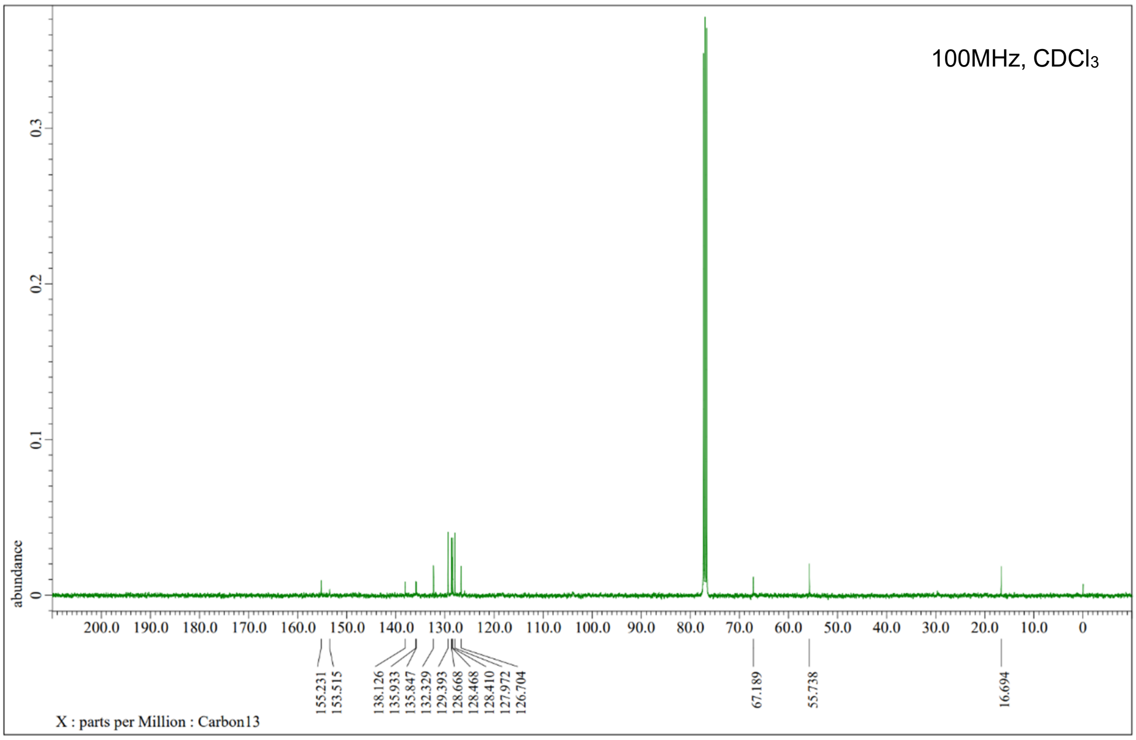
100MHz, CDCl3
X : parts per Million : Carbon13
abundance

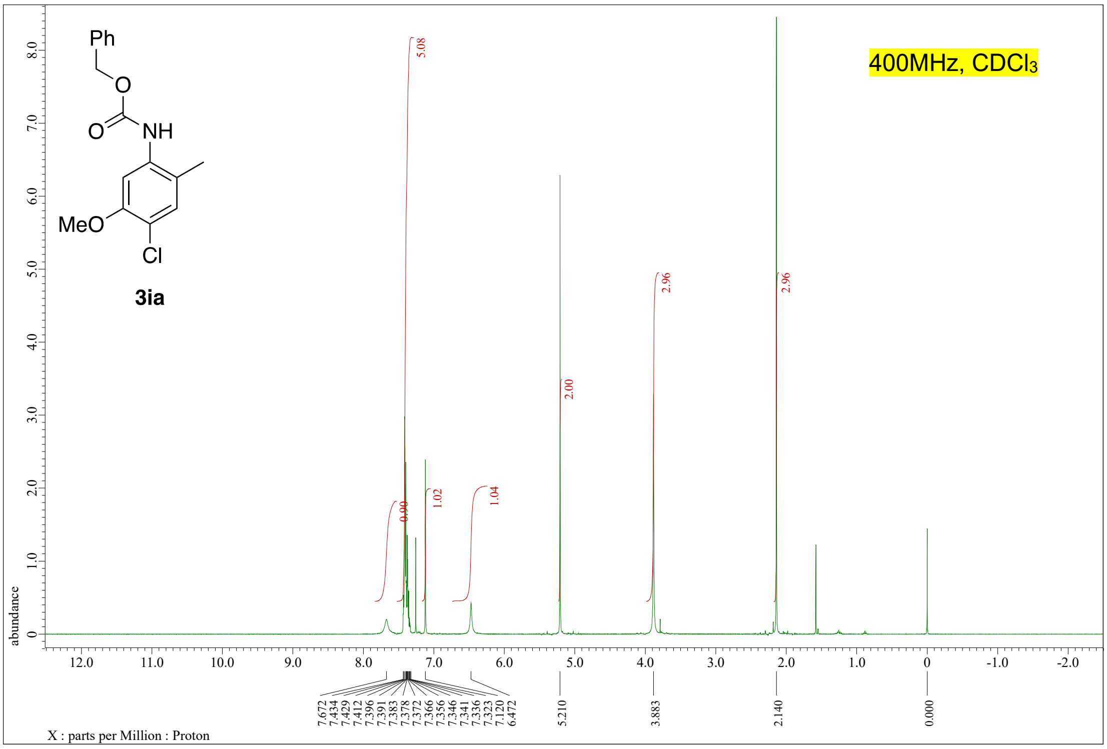
Ph
O
O
NH
MeO
Cl
3ia
400MHz, CDCl3
5.08
0.90
1.02
1.04
2.00
2.96
2.96
abundance
X : parts per Million : Proton
7.672
7.434
7.429
7.412
7.396
7.391
7.383
7.378
7.372
7.366
7.356
7.346
7.341
7.336
7.323
7.120
6.472
5.210
3.883
2.140
0.000

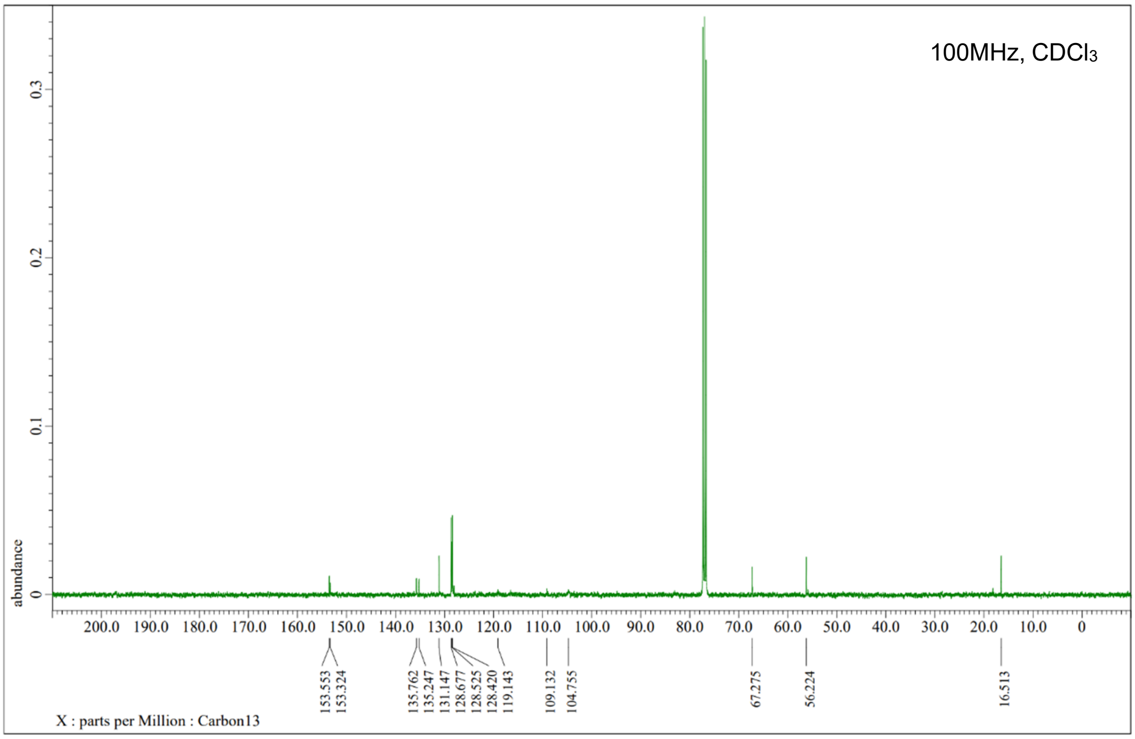
100MHz, CDCl3
abundance
X : parts per Million : Carbon13
153.553
153.324
135.762
135.247
131.147
128.677
128.525
128.420
119.143
109.132
104.755
67.275
56.224
16.513

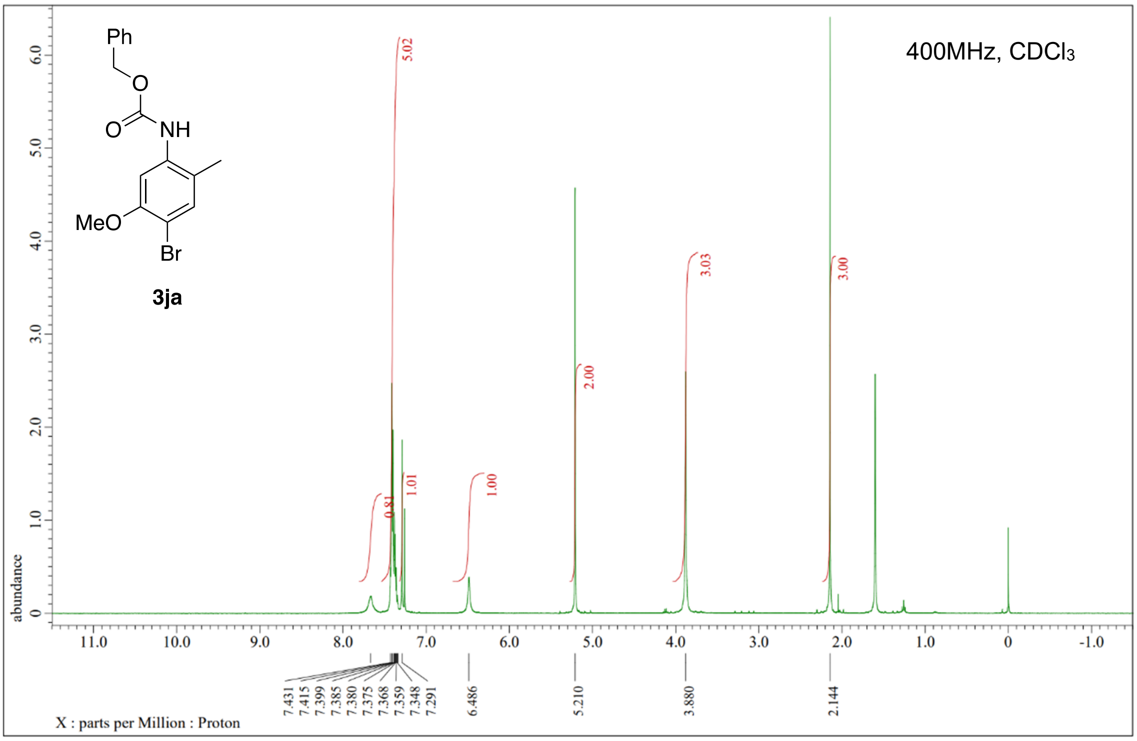
Ph
O
O
NH
MeO
Br
3ja
400MHz, CDCl3
5.02
0.81
1.01
1.00
2.00
3.03
3.00
abundance
X : parts per Million : Proton
7.431
7.415
7.399
7.385
7.380
7.375
7.368
7.359
7.348
7.291
6.486
5.210
3.880
2.144

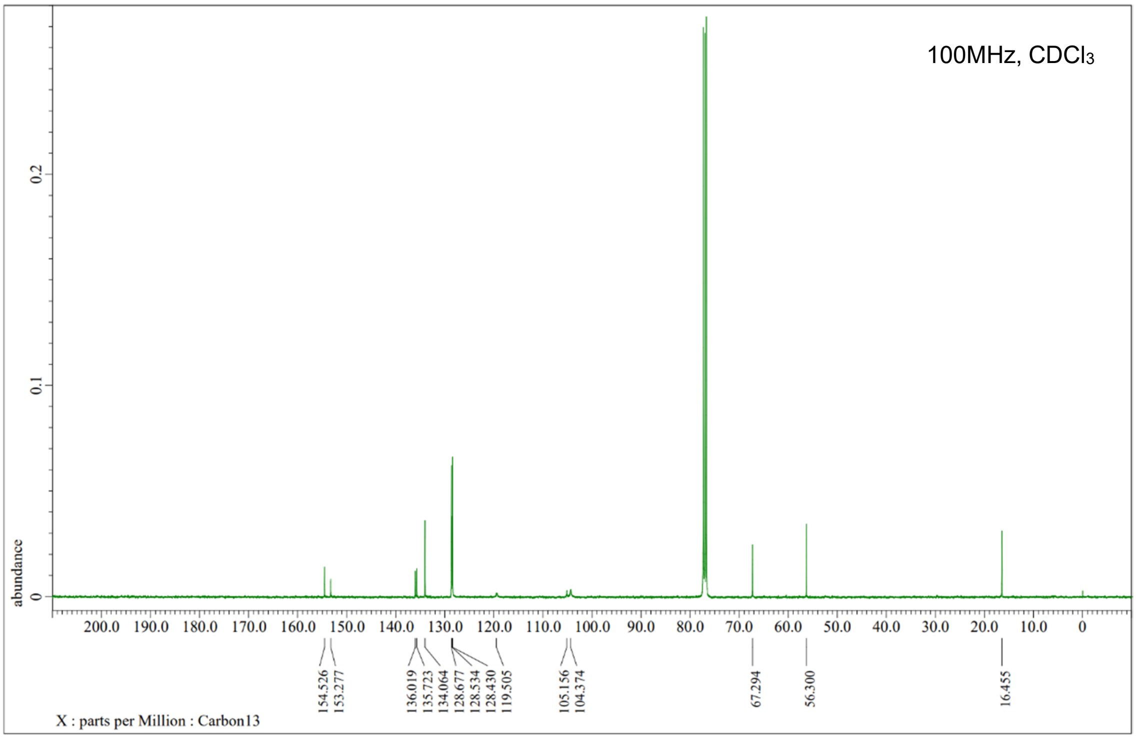
100MHz, CDCl3
abundance
X : parts per Million : Carbon13
154.526
153.277
136.019
135.723
134.064
128.677
128.534
128.430
119.505
105.156
104.374
67.294
56.300
16.455

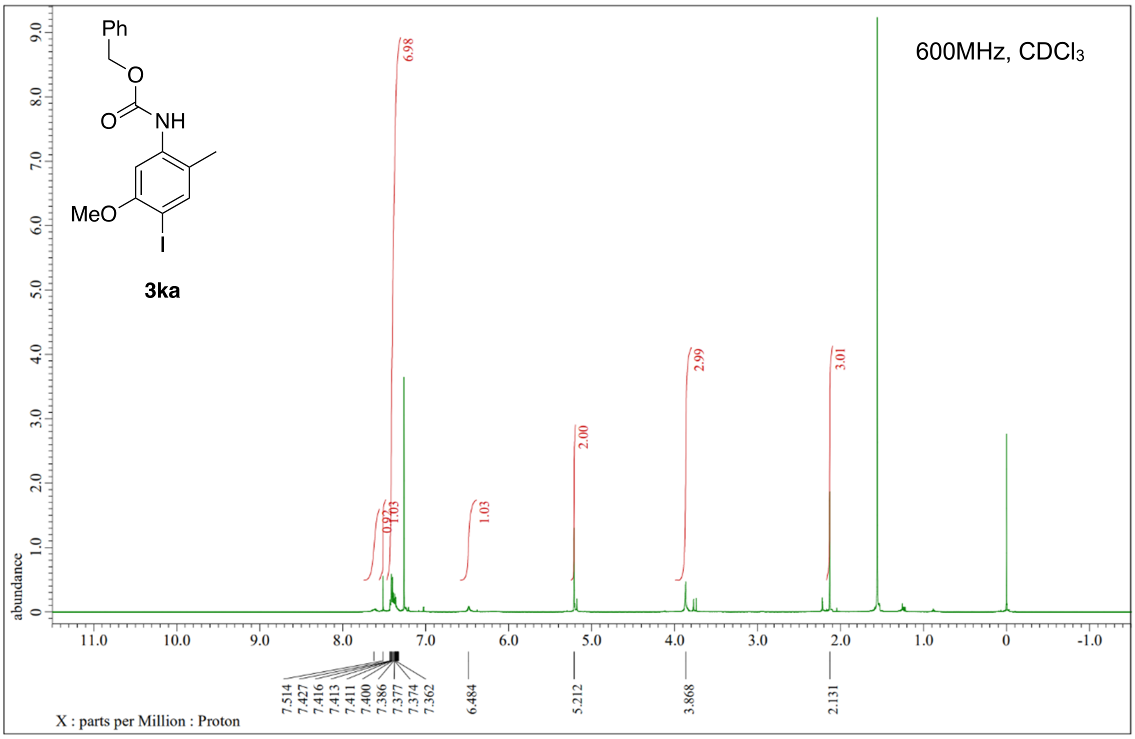
Ph
O
O
NH
MeO
I
3ka
600MHz, CDCl3
abundance
X : parts per Million : Proton
7.514 7.427 7.416 7.413 7.411 7.400 7.386 7.377 7.374 7.362
6.484
5.212
3.868
2.131
0.97 1.03 6.98 1.03 2.00 2.99 3.01

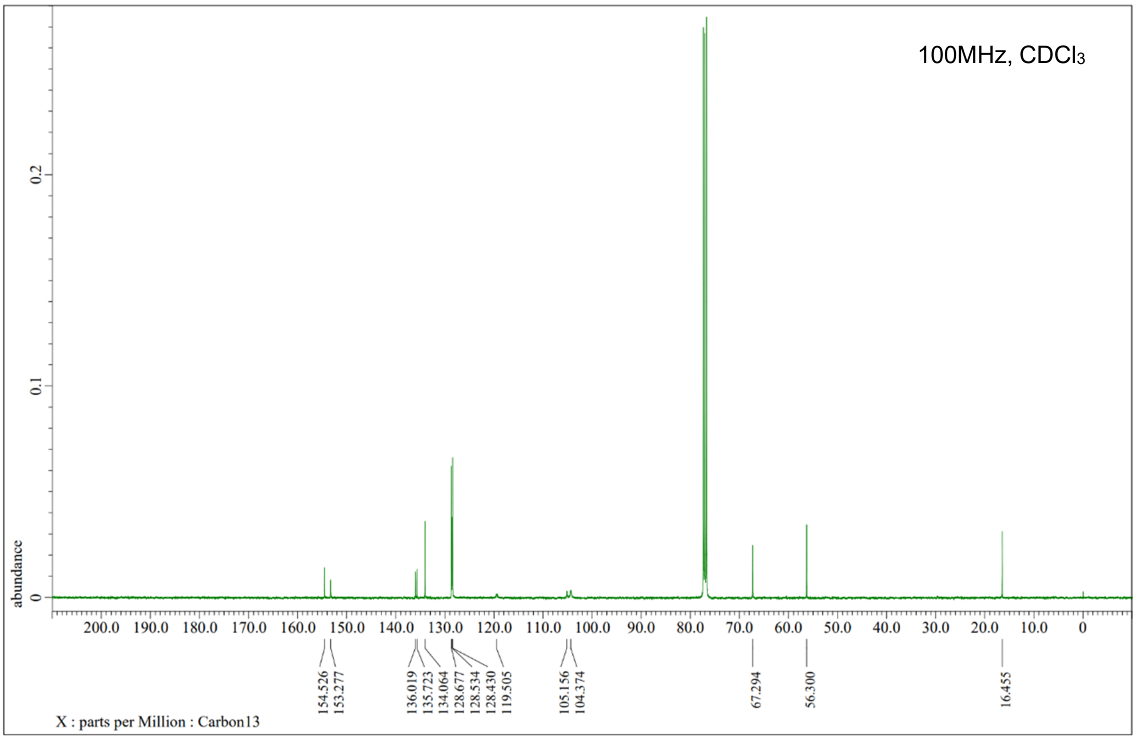
100MHz, CDCl3
abundance
X : parts per Million : Carbon13
154.526 153.277
136.019 135.723 134.064 128.677 128.534 128.430 119.505
105.156 104.374
67.294
56.300
16.455

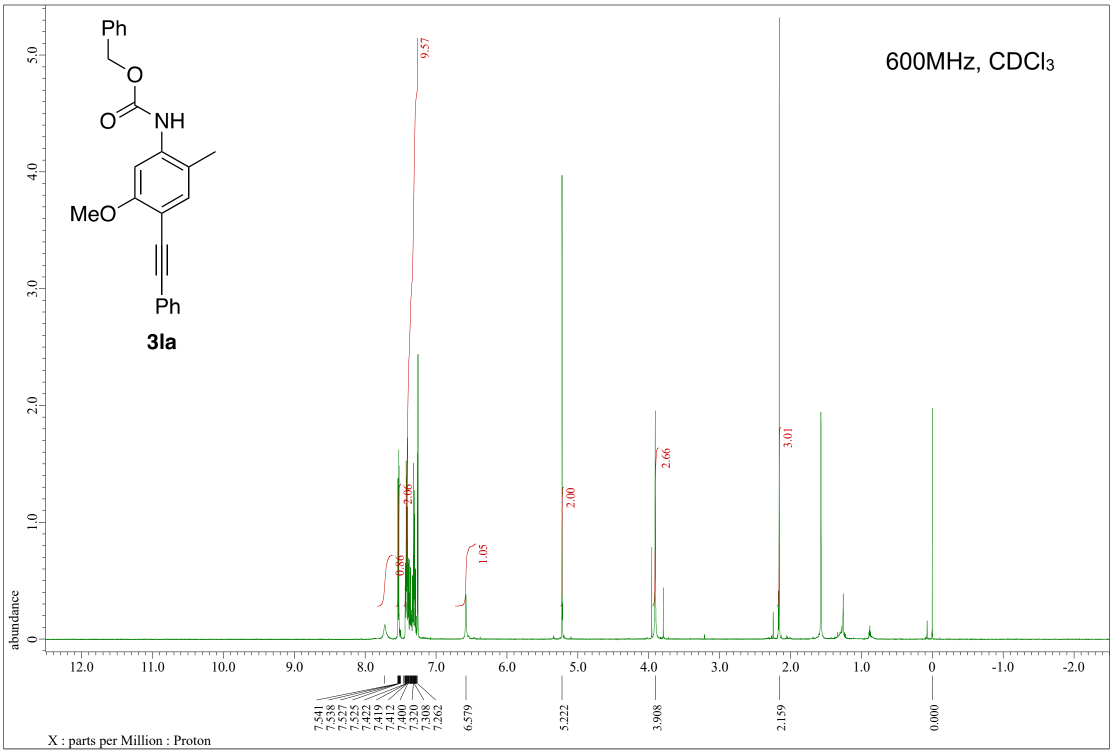
600MHz, CDCl3
3la
abundance
X : parts per Million : Proton
7.541
7.538
7.527
7.525
7.422
7.419
7.412
7.400
7.320
7.308
7.262
6.579
5.222
3.908
2.159
0.000
9.57
2.66
3.01
2.00
2.06
1.05
0.86

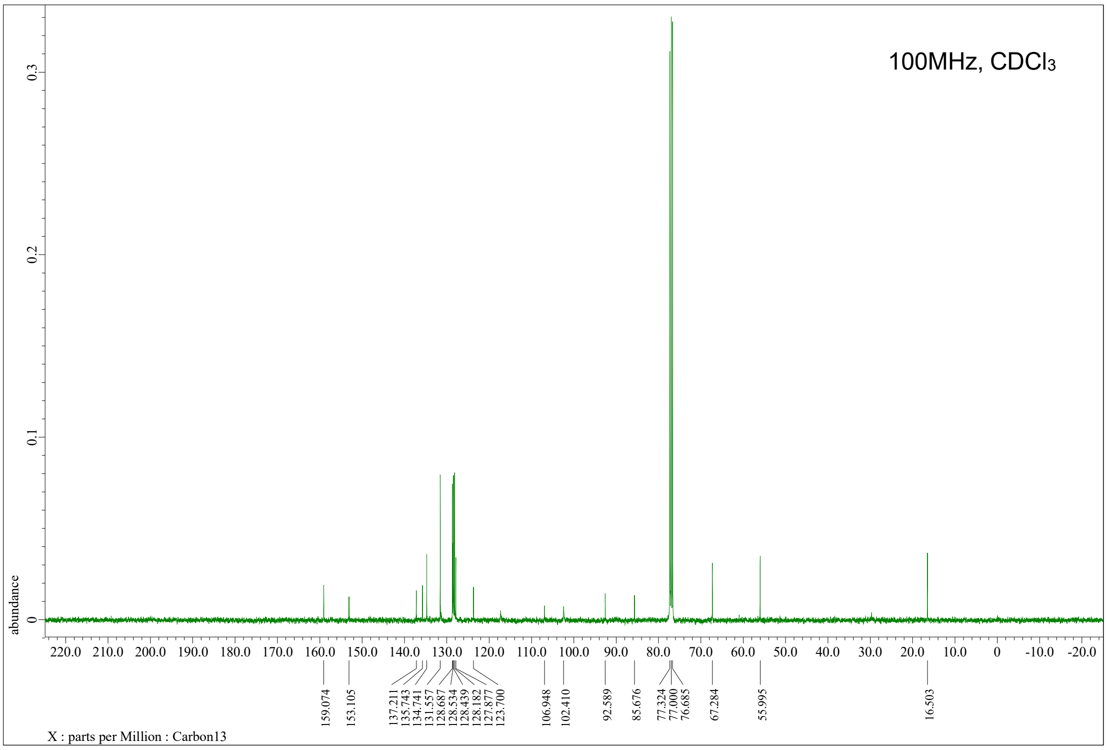
100MHz, CDCl3
abundance
X : parts per Million : Carbon13
159.074
153.105
137.211
135.743
134.741
131.557
128.687
128.534
128.439
128.182
127.877
123.700
106.948
102.410
92.589
85.676
77.324
77.000
76.685
67.284
55.995
16.503

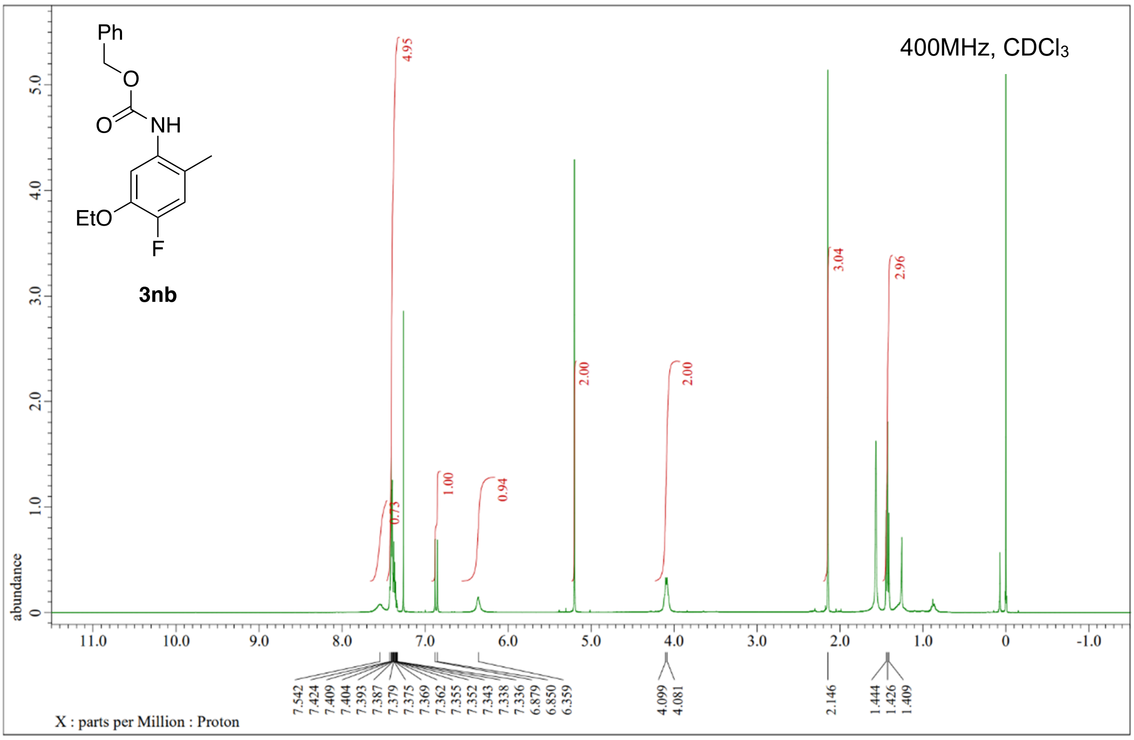

400MHz, CDCl3
Ph
O
O
NH
EtO
F
3nb
X : parts per Million : Proton
abundance
7.542
7.424
7.409
7.404
7.393
7.387
7.379
7.375
7.369
7.362
7.355
7.352
7.343
7.338
7.336
6.879
6.850
6.359
4.099
4.081
2.146
1.444
1.426
1.409
4.95
0.73
1.00
0.94
2.00
2.00
3.04
2.96

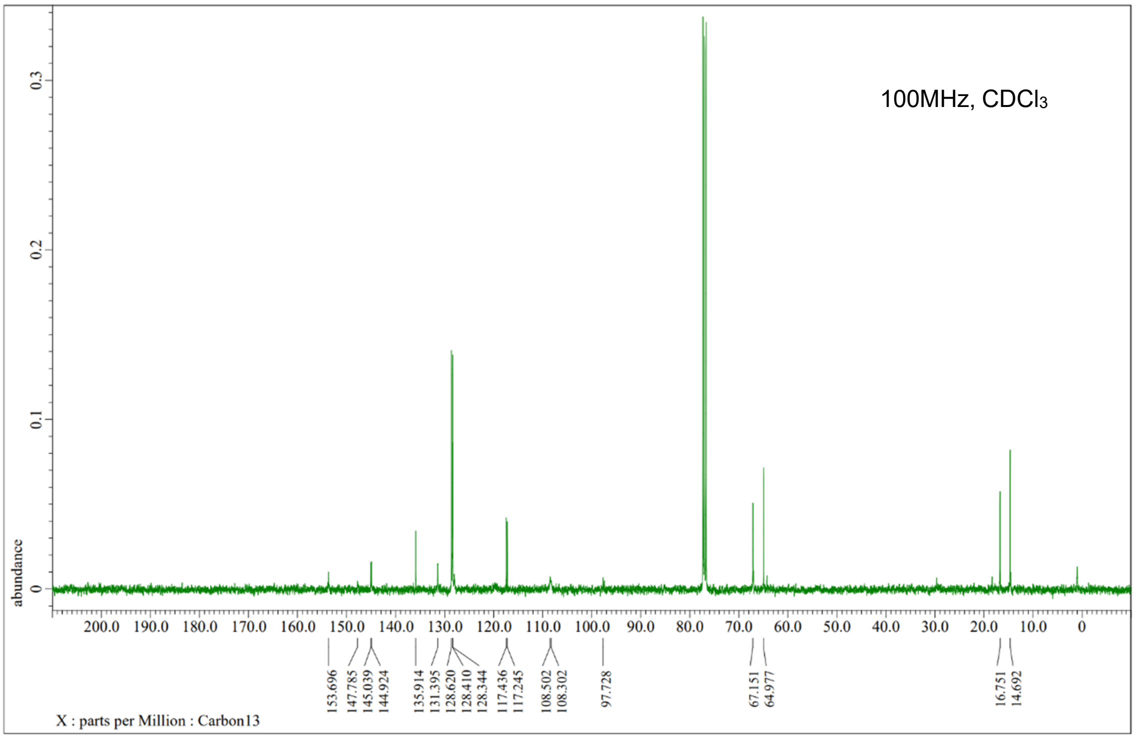

100MHz, CDCl3
X : parts per Million : Carbon13
abundance
153.696
147.785
145.039
144.924
135.914
131.395
128.620
128.410
128.344
117.436
117.245
108.502
108.302
97.728
67.151
64.977
16.751
14.692

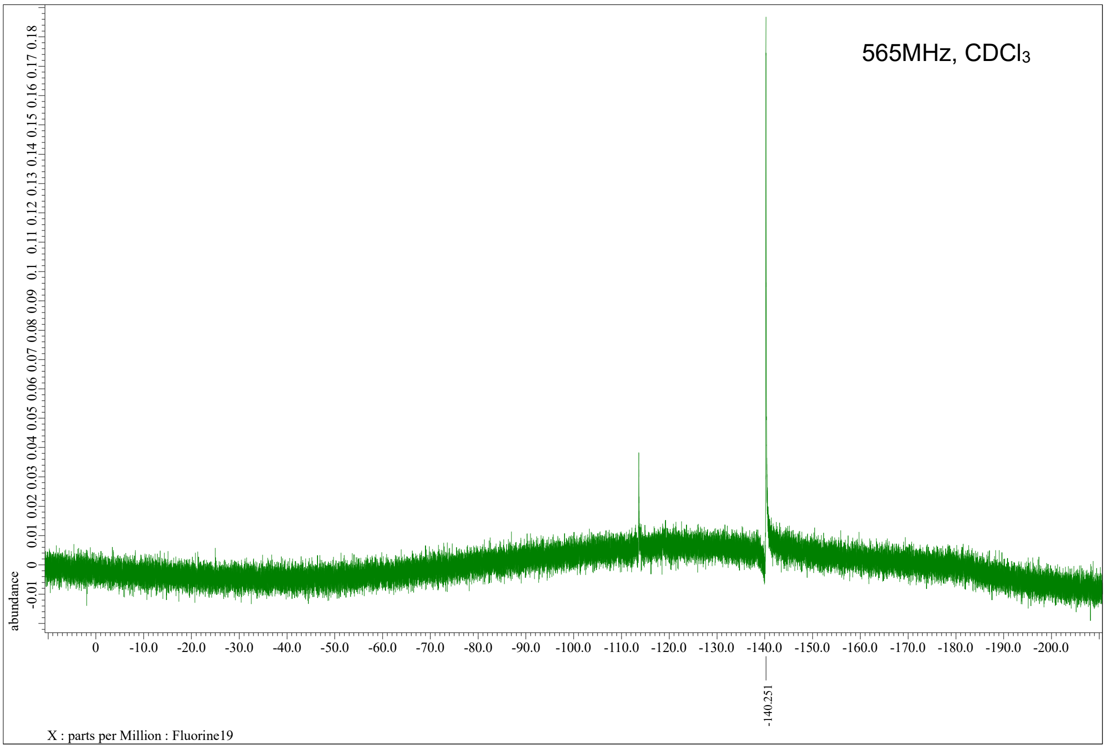
565MHz, $CDCl_3$
abundance
-140.251
X : parts per Million : Fluorine19

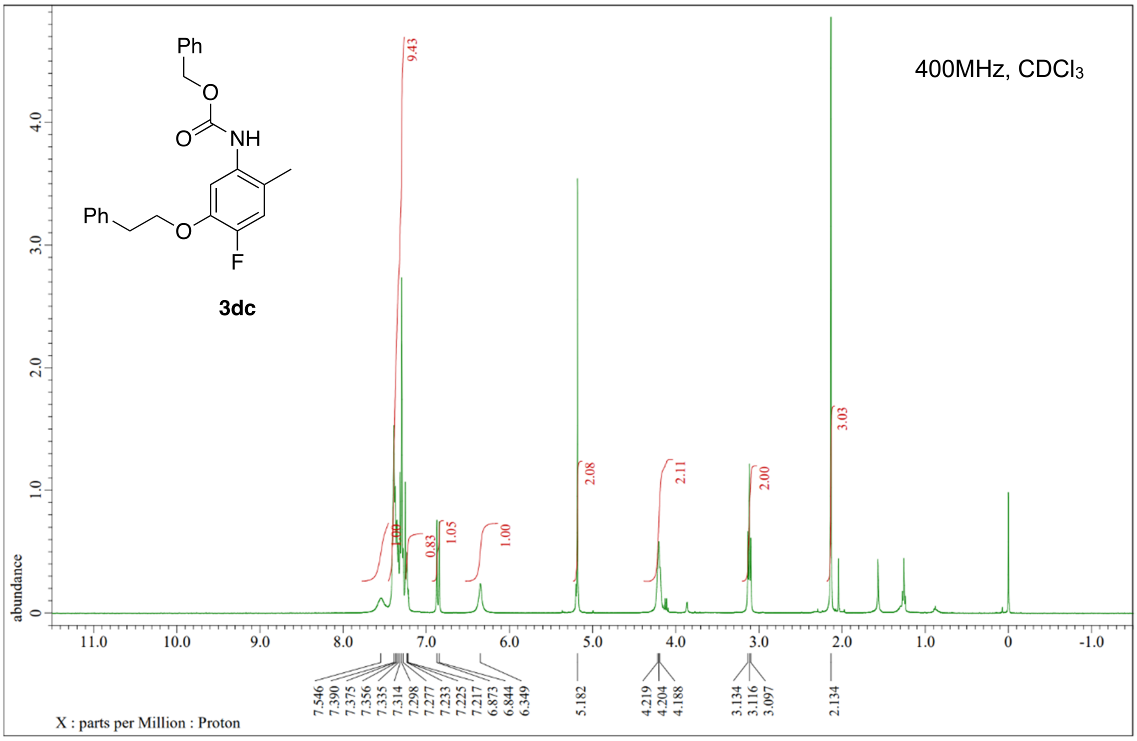
400MHz, CDCl3
3dc
X : parts per Million : Proton

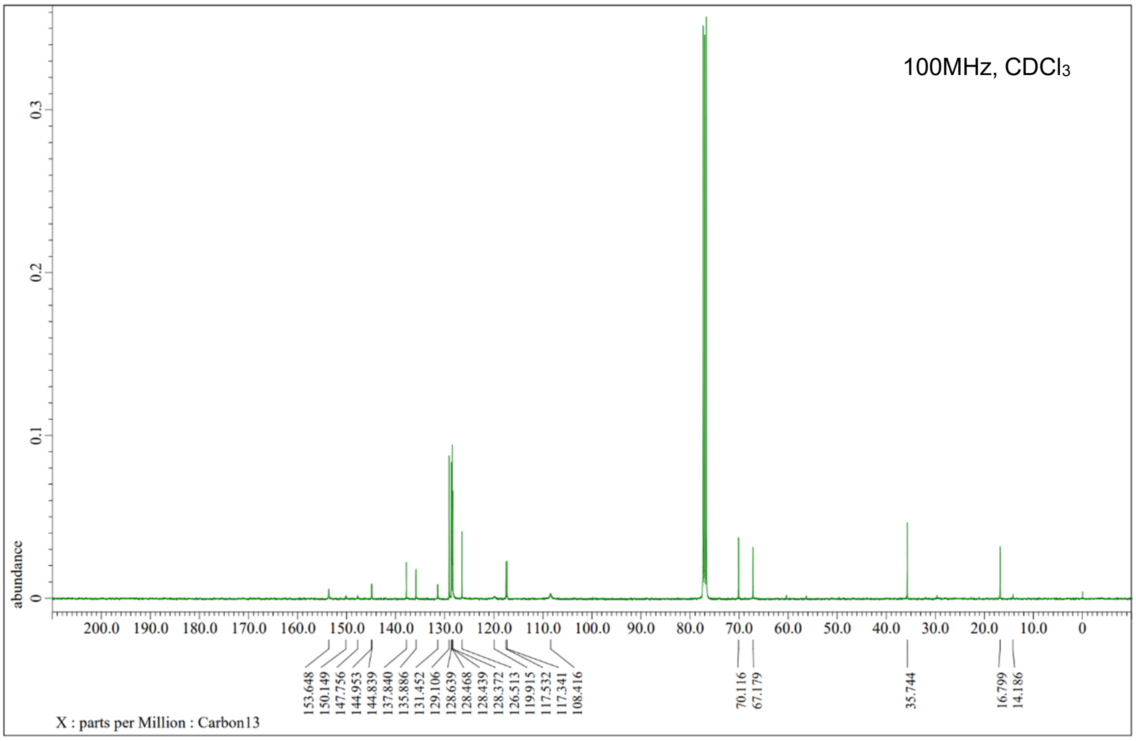
100MHz, CDCl3
X : parts per Million : Carbon13

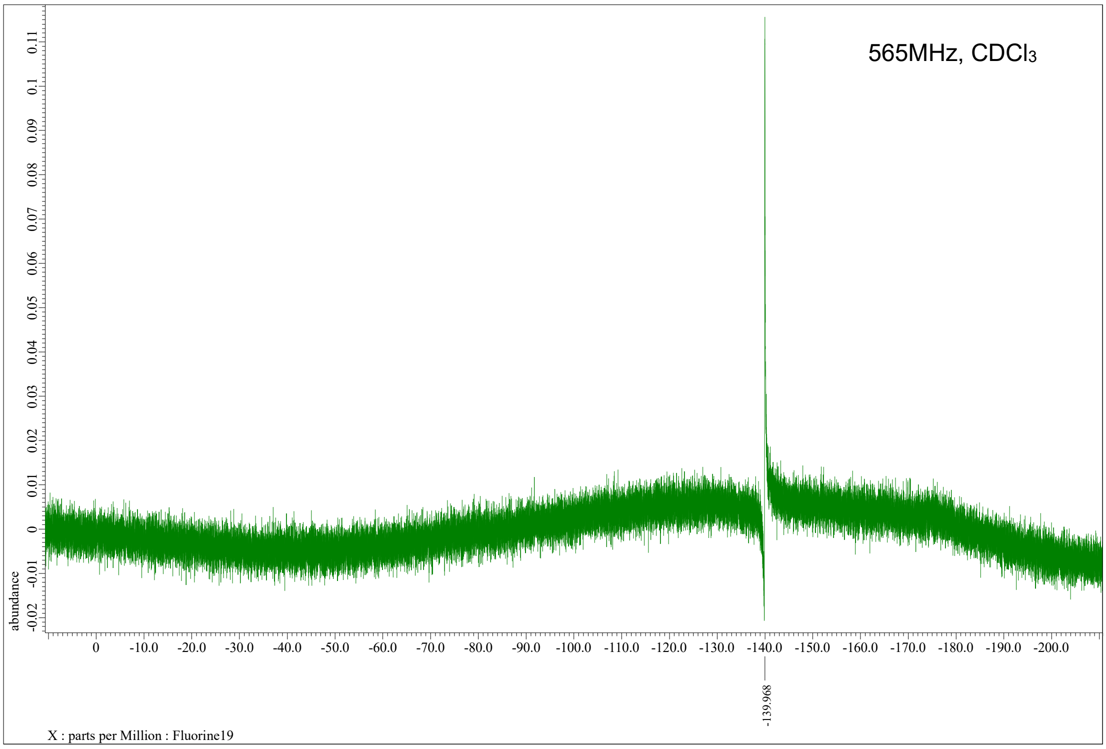
565MHz, CDCl3
abundance
-139.968
X : parts per Million : Fluorine19

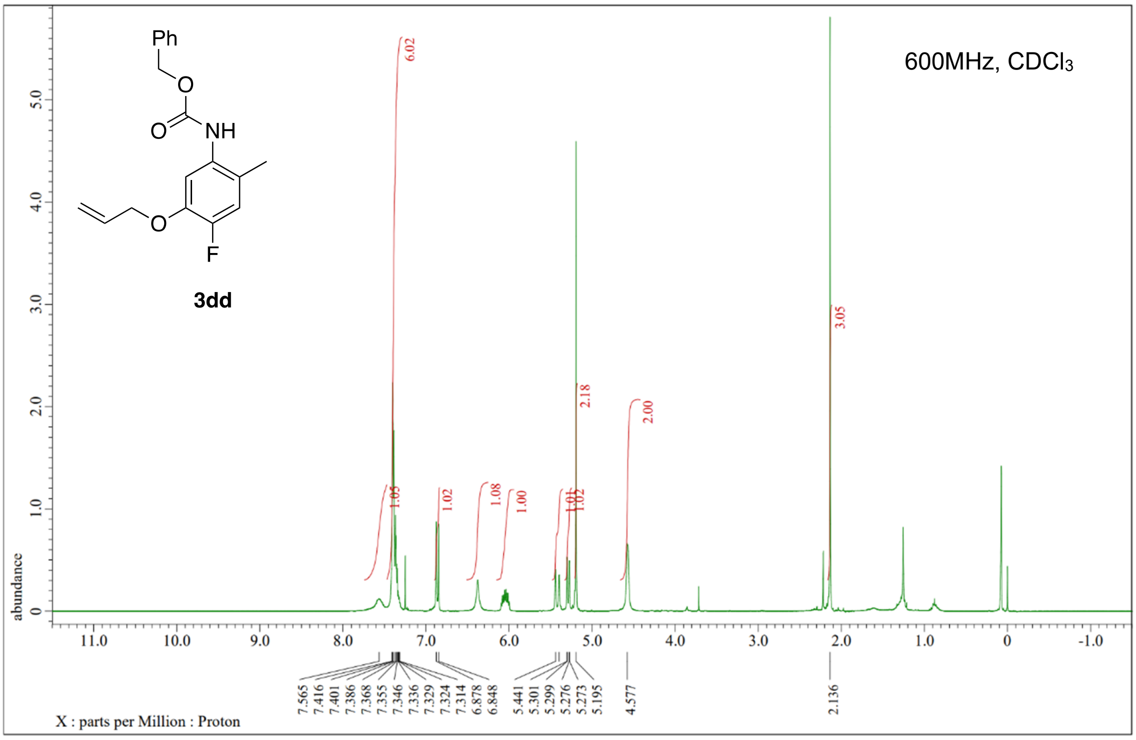
600MHz, CDCl3
3dd
Ph
O
O
NH
F
X : parts per Million : Proton
abundance
7.565
7.416
7.401
7.386
7.368
7.355
7.346
7.336
7.329
7.324
7.314
6.878
6.848
5.441
5.301
5.299
5.276
5.273
5.195
4.577
2.136

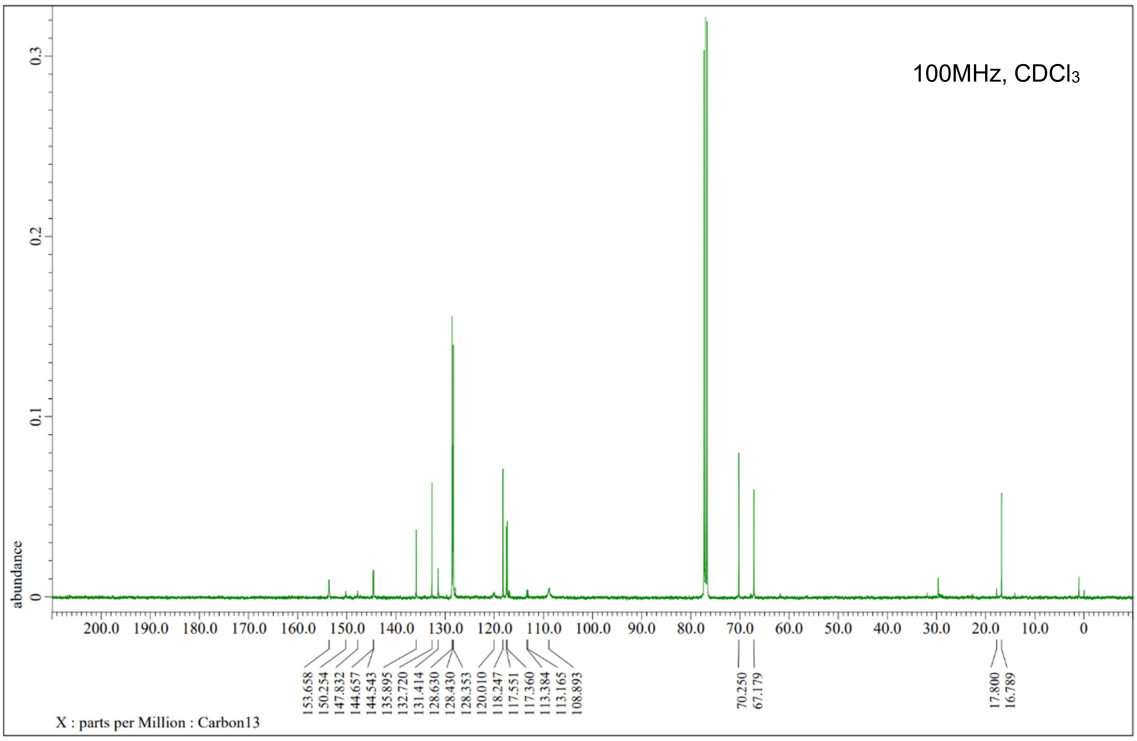
100MHz, CDCl3
X : parts per Million : Carbon13
abundance
153.658
150.254
147.832
144.657
144.543
135.895
132.720
131.414
128.630
128.430
128.353
120.010
118.247
117.551
117.360
113.384
113.165
108.893
70.250
67.179
17.800
16.789

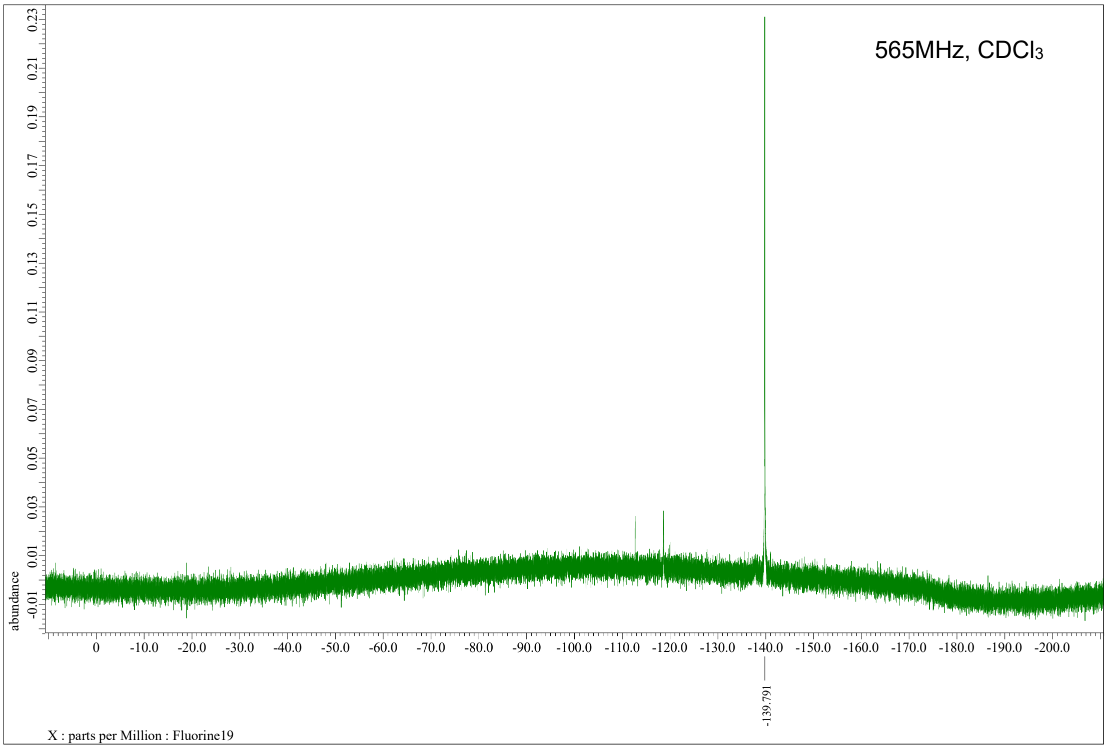
565MHz, CDCl3
abundance
X : parts per Million : Fluorine19
-139.791

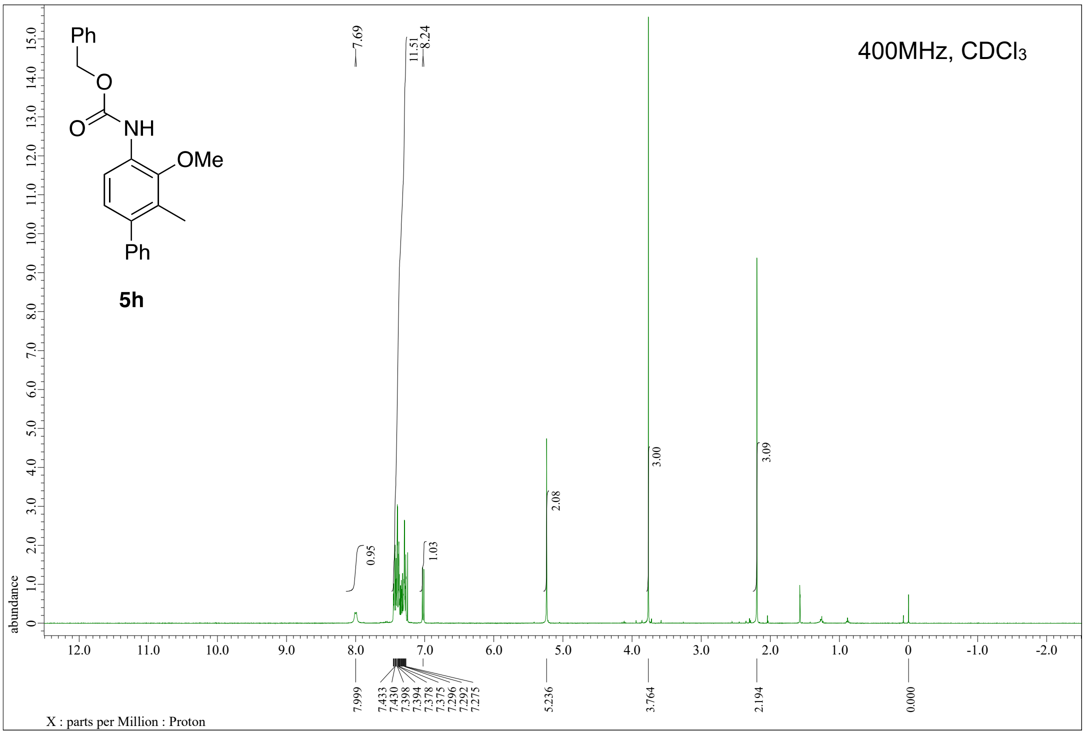
Ph
O
O
NH
OMe
Ph
5h
400MHz, CDCl3
7.69
11.51
8.24
0.95
1.03
2.08
3.00
3.09
abundance
7.999
7.433 7.430 7.398 7.394 7.378 7.375 7.296 7.292 7.275
5.236
3.764
2.194
0.000
X : parts per Million : Proton

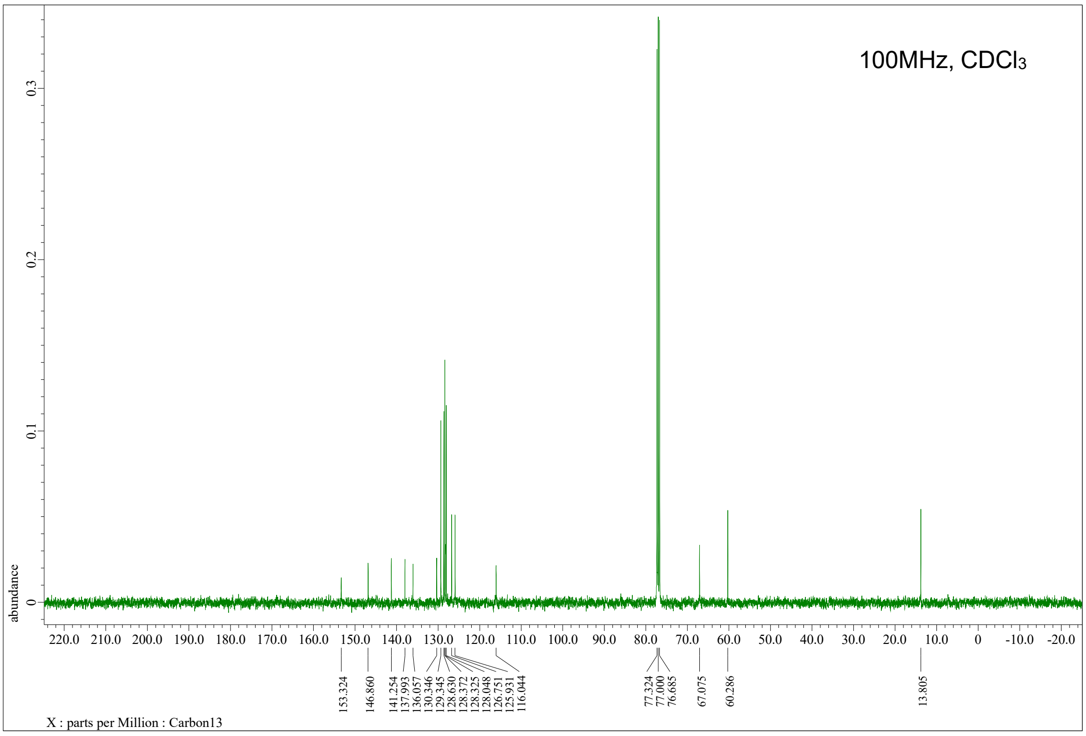
100MHz, CDCl3
abundance
153.324
146.860
141.254 137.993 136.057 130.346 129.345 128.630 128.372 128.325 128.048 126.751 125.931 116.044
77.324 77.000 76.685
67.075
60.286
13.805
X : parts per Million : Carbon13

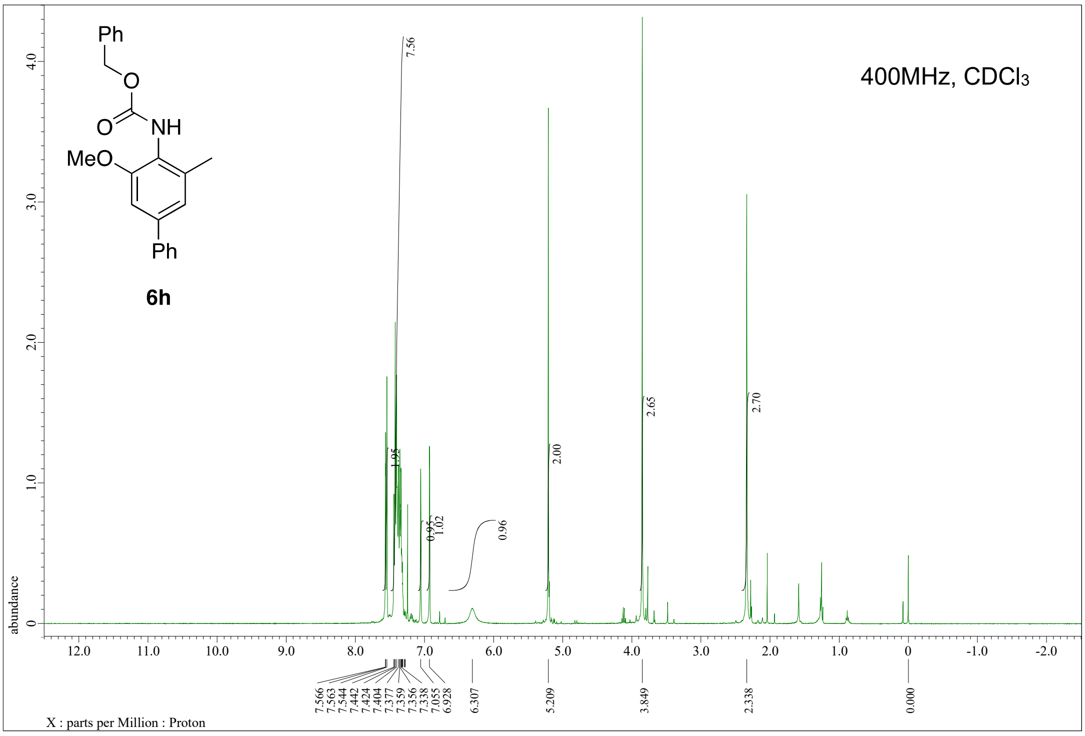
400MHz, CDCl3
6h
X : parts per Million : Proton
abundance

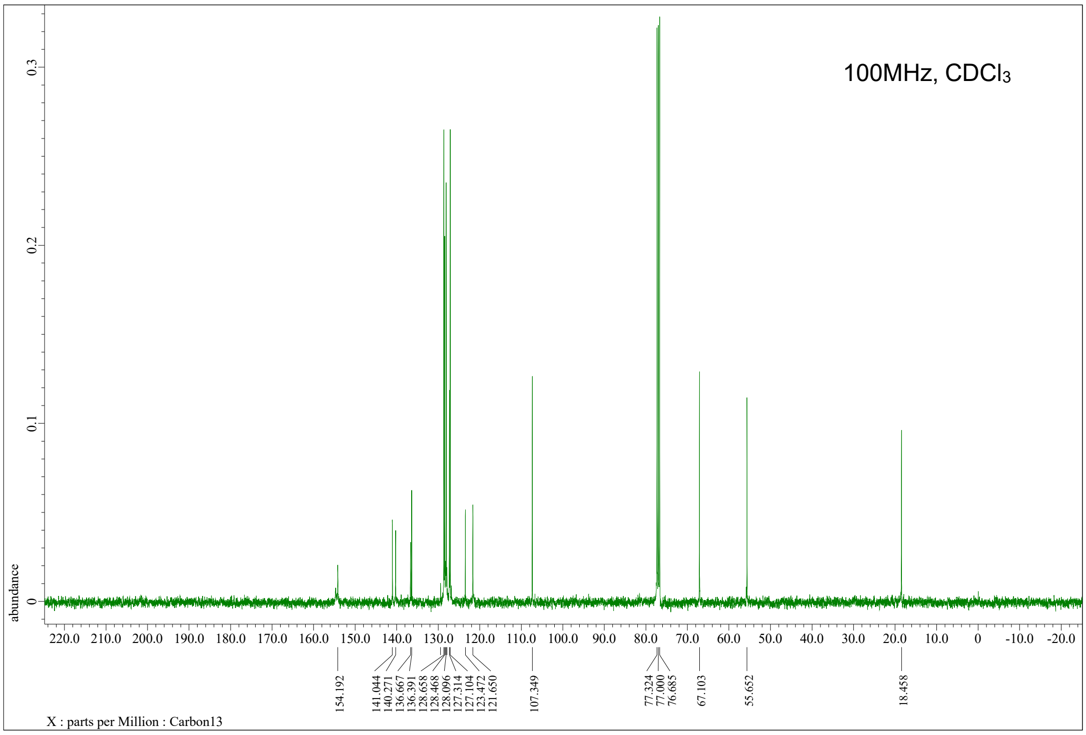
100MHz, CDCl3
X : parts per Million : Carbon13
abundance
